# The complex Maxwell stress tensor theorem: The imaginary stress tensor and the reactive strength of orbital momentum. A novel scenery underlying electromagnetic optical forces

**DOI:** 10.1038/s41377-022-00979-2

**Published:** 2022-10-12

**Authors:** Manuel Nieto-Vesperinas, Xiaohao Xu

**Affiliations:** 1 Instituto de Ciencia de Materiales de Madrid, Consejo Superior de Investigaciones Científicas. Campus de Cantoblanco, Madrid, 28049 Spain; 2 State Key Laboratory of Transient Optics and Photonics, Xi’an Institute of Optics and Precision Mechanics, Chinese Academy of Sciences, Xi’an, 710119 China; 3 Institute of Nanophotonics, Jinan University, Guangzhou, 511443 China

**Keywords:** Optics and photonics, Optical physics

## Abstract

We uncover the existence of a universal phenomenon concerning the electromagnetic optical force exerted by light or other electromagnetic waves on a distribution of charges and currents in general, and of particles in particular. This conveys the appearence of underlying reactive quantities that hinder radiation pressure and currently observed time-averaged forces. This constitutes a novel paradigm of the mechanical efficiency of light on matter, and completes the landscape of the optical, and generally electromagnetic, force in photonics and classical electrodynamics; widening our understanding in the design of both illumination and particles in optical manipulation without the need of increasing the illuminating power, and thus lowering dissipation and heating. We show that this may be accomplished through the minimization of what we establish as the reactive strength of orbital (or canonical) momentum, which plays against the optical force a role analogous to that of the reactive power versus the radiation efficiency of an antenna. This long time overlooked quantity, important for current progress of optical manipulation, and that stems from the complex Maxwell theorem of conservation of complex momentum that we put forward, as well as its alternating flow associated to the imaginary part of the complex Maxwell stress tensor, conform the imaginary Lorentz force that we introduce in this work, and that like the reactive strength of orbital momentum, is antagonistic to the well-known time-averaged force; thus making this reactive Lorentz force indirectly observable near wavelengths at which the time-averaged force is lowered. The Minkowski and Abraham momenta are also addressed.

## Abstract (web summary)

The imaginary Maxwell stress tensor IMST and the reactive strength of orbital momentum ROM are established. It is shown that they universally produce reactive electromagnetic forces ILFs that counteract all currently observed time-averaged optical forces RLFs.

## Graphical abstract

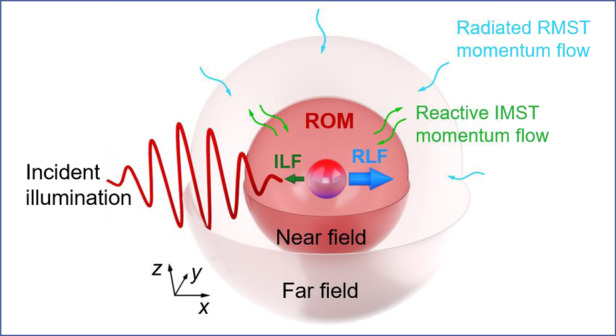

## Introduction

The Maxwell stress tensor in whose terms the conservation of linear and angular momentum is expressed^1,2^, is at the root of electromagnetic forces in general and optical manipulation in particular^3–9^. When the fields are characterized by complex functions, this conservation law is obtained from the real parts which yield time-averaged, or real, Lorentz forces (RLF) and torques. This is extensively employed, in particular, for time-harmonic (i.e., monochromatic) fields^6,8,10,11^.

In this context, it is well-known that the RLF on a volume *V*_0_ of charges and currents is given by the momentum flux whose density is the real part of the Maxwell stress tensor (RMST) across any contour ∂*V* enclosing *V*_0_. In consequence this RLF may be considered as the flow, characterized by the RMST, into the surface of a sphere in the far-field, i.e., in the radiation zone of *V*_0_ and, as such, it may be considered a “radiation force”.

In this paper, we demonstrate that this theory through the RMST describes only half the physics of the electromagnetic optical force. The other half, so far ignored and that we uncover here by establishing the complex Maxwell stress tensor theorem, is characterized by the imaginary part of the complex Maxwell stress tensor (CMST), related to the exchange of reactive (i.e., imaginary Poynting) momentum (IPM)^12^, and acquires importance as optical manipulation of matter progresses and expands its scope incorporating reactive concepts^12–14^. The imaginary Maxwell stress tensor (IMST) builds-up in and around *V*_0_ what we find and put forward here: the reactive strength of orbital (or canonical) momentum (ROM); so that this storage of ROM contributes to the imaginary Lorentz force (ILF) on *V*_0_ which, as we shall show, may also be envisaged as a reactive strength of Poynting momentum. This reactive force is not observable on time averaging since its net value is zero, but it exists instantaneously due to the transfer of the reactive momentum, which alternates with time, between the wave and the body.

Hence, the ILF is a basically fundamental dynamic phenomenon, inherent to the emergence of electromagnetic optical forces, being also associated to the appearance of reactive energy, reactive work, and reactive helicity^12–14^. The former having been for many years a well-known workhorse in the design of RF antennas^15–18^, and recently studied in nano and micro-antennas^12–14,19^. Therefore, like in RF antenna design one aims to diminish the reactive power and reactive work to increase the radiation efficiency, the theory put forward in this work constitutes a tool to act on the ROM and ILF in order to optimize a desired radiation pressure in optical manipulation.

Consequently, here we show that, as such, the ILF and ROM play an antagonic role with respect to the standard RLF, so that a strong ILF, and thus a large ROM storage, amounts to a loss of radiative force, RLF, and vice-versa. This makes the ROM and ILF indirectly observable.

It is somewhat striking that having existed for decades the complex Poynting theorem and its consequent reactive quantities: the IPM, reactive work, and reactive energy; to our knowledge, the complex Maxwell stress tensor theorem, and the reactive entities it conveys, had not been established. This might be due to the practical difficulties involved in optical manipulation. However the fast advances and present maturity of the optical handling of matter, now warrant their formulation.

In our view, this novel scenario completes an interpretative panorama of forces in the science of light and classical electrodynamics, e.g., in the design of particles and of structured beam illumination that, as done with their radiative power and emitted field helicity^12,20^, the efficiency of the time-averaged force, i.e., of the RLF acting on them, be optimized by either enhancing or weakening it.

The outline of this paper is as follows:

First, we establish the complex stress tensor theorem in an embedding vacuum or air; defining the ILF, IMST, and ROM for general time-dependent light fields, and discussing their respective physical meaning. Then we address these concepts for time-harmonic (or monochromatic) wavefields to which the rest of this work is devoted.

Secondly, we shall characterize the IMST flow in terms of the magnetic and electric spin momenta of the total (i.e., incident plus scattered) field, which we introduce from first Lagrangian principles. We then express the ILF by what we put forward as the *reactive strength of Poynting momentum*, obtained from the electric and magnetic spin and orbital momenta, while we show that the RLF may be written as the sum of the imaginary spin and orbital momenta. We also establish the imaginary field (i.e., Poynting) momentum representation with sources in terms of the reactive orbital and spin momenta^12^.

After demonstrating the near-field nature of the IMST, we consider the extensively studied case of a dipolar particle, deriving the alternating imaginary momentum flow IMST across the surface of a surrounding sphere in the near-field, along with the ROM and ILF; showing that, in contrast with the field (i.e., Poynting) momentum flow RMST, and the RLF, these quantities depend on the sphere radius.

Examples are given, comparing numerical results and theory, for three dipolar archetypical particles: a low-index dielectric, a high-index magnetoelectric one, and a plasmonic sphere. It is also shown that an heuristic direct derivation of the ILF, analogous to that employed in ref. ^5^ for the RLF, works well for low index dielectric particles, but not for resonant ones, which first require the use of the above-mentioned IMST calculation.

Finally, a recapitulation of the CMST and the reactive force is given when one considers a homogeneous, linear, isotropic dielectric as the embedding medium; establishing the time-dependent CMST theorem, reactive force, and ROM according to whether one chooses a complex value generalization of the Minkowski or Abraham field momentum. Demonstrating that in the case of time-harmonic waves, the reactive force, like the time-averaged force, is independent of the choice of a Minkowski or an Abraham complex Poynting momentum.

## Time-dependent fields: The complex Maxwell stress tensor theorem. The imaginary stress tensor and the reactive strength of orbital momentum

In our study, we use Gaussian units and assume a homogeneous medium with relative permittivity and permeability: *ϵ* = *μ* = 1, (i.e., vacuum), embedding the illuminated body. A convenient way to frame the following theory is to start with analytic signals^21,22^ as done with the complex Poynting theorem^23,24^. These are ${{\vec{\bf\mathcal{E}}}}({{{\bf{r}}}},\tau )$, ${{\vec{\bf\mathcal{H}}}}({{{\bf{r}}}},\tau )$ and $\vec{{{\bf\mathcal{J}}}}({{{\bf{r}}}},\tau )$, associated to the real vectors $\vec{\bf\mathfrak{E}}({{{\bf{r}}}},t)$, $\vec{\bf\mathfrak{H}}({{{\bf{r}}}},t)$ and $\vec{\bf\mathfrak{J}}({{{\bf{r}}}},t)$ which are analytically continued into the lower half complex plane *τ* = *t* − i*s*. Generically denoting each of these analytic functions as $\vec{{{\bf\mathcal{V}}}}({{{\bf{r}}}},t,s)$, they are expressed by the Fourier integral^21,22^:

1 $$\vec{{{\bf\mathcal{V}}}}({{{\bf{r}}}},t,s)\equiv \vec{{{\bf\mathcal{V}}}}({{{\bf{r}}}},\tau )=\int\nolimits_{0}^{\infty }d\omega \exp (-{{{\rm{i}}}}\omega \tau )\vec{{\bf\mathfrak{V}}}_{\omega }({{{\bf{r}}}},\omega )$$

$\vec{{\bf\mathfrak{V}}}_{\omega }({{{\bf{r}}}},\omega )$ being the *ω*-Fourier spectrum of the real function $\vec{\bf\mathfrak{V}}({{{\bf{r}}}},t)$ which generically denotes either $\vec{\bf\mathfrak{E}}({{{\bf{r}}}},t)$, $\vec{\bf\mathfrak{B}}({{{\bf{r}}}},t)$ or $\vec{\bf\mathfrak{J}}({{{\bf{r}}}},t)$. Then, (1) allows us to write the Hilbert transformation:

2 $$\begin{array}{lll}\vec{{{\bf\mathcal{V}}}}({{{\bf{r}}}},t,s)&\equiv& \vec{{{\bf\mathcal{V}}}}({{{\bf{r}}}},\tau )=\frac{{{{\rm{i}}}}}{2\pi }\int\nolimits_{-\infty }^{\infty }\,dt^{\prime} \,\frac{\vec{\bf\mathfrak{V}}({{{\bf{r}}}},t^{\prime} )}{t^{\prime} -\tau }\\ &=&\vec{\bf\mathfrak{V}}({{{\bf{r}}}},t)* {C}_{s}(t),\,\,{C}_{s}(t)=\frac{-{{{\rm{i}}}}}{2\pi (t-{{{\rm{i}}}}s)}\,\,\,=\frac{1}{2\pi }\,\frac{s-{{{\rm{i}}}}t}{{t}^{2}+{s}^{2}}\end{array}$$

Where the symbol * denotes convolution. Hence $\vec{{{\bf\mathcal{V}}}}({{{\bf{r}}}},t,s)$ is obtained by time-averaging the physical function $\vec{\bf\mathfrak{V}}({{{\bf{r}}}},t)$ over the low pass Cauchy filter *C*_*s*_(*t*) = − i/[2*π*(*t* − i*s*)], whose real and imaginary parts, (being a Kramers-Krőnig pair), are a Lorentzian of width Δ*t* = 2*s* and an odd function resulting from the product of this Lorentzian by −*t*/*s*. Hence 2*s* constitutes the *minimum time interval* with which the quantity $\vec{{{\mathcal{V}}}}({{{\bf{r}}}},t,s)$ can be *resolved*, and Δ*t* is a *time resolution scale* for the analytic signals $\vec{{{\mathcal{V}}}}({{{\bf{r}}}},\tau )$ associated to the real quantities $\vec{\mathfrak{V}}({{{\bf{r}}}},t)$^23,24^. In antenna and circuit theory, *s* is known as *reactive time*, (measured in second reactive, sr)^23,24^.

Introducing the complex derivatives:

3 $${\partial }_{\tau }=\frac{1}{2}({\partial }_{t}+{{{\rm{i}}}}{\partial }_{s})\quad {\partial }_{\tau }^{* }=\frac{1}{2}({\partial }_{t}-{{{\rm{i}}}}{\partial }_{s})$$

4 $${\partial }_{\tau }\vec{{{{\mathcal{V}}}}}^{* }={\partial }_{t}\vec{{{{\mathcal{V}}}}}^{* }={{{\rm{i}}}}{\partial }_{s}\vec{{{{\mathcal{V}}}}}^{* },\quad {\partial }_{\tau }^{* }\vec{{{\mathcal{V}}}}={\partial }_{t}\vec{{{\mathcal{V}}}}=-{{{\rm{i}}}}{\partial }_{s}\vec{{{\mathcal{V}}}}$$

the Maxwell equations: $\nabla \cdot \vec{\mathfrak{E}}=4\pi \rho$, $\nabla \cdot \vec{\mathfrak{H}}=0$, $\nabla \times \vec{\mathfrak{E}}=-(1/c){\partial }_{t}\vec{\mathfrak{H}}$ and $\nabla \times \vec{\mathfrak{H}}=(1/c){\partial }_{t}\vec{\mathfrak{H}}+(4\pi /c)\vec{\mathfrak{J}}$, yield for the analytic signals associated to the fields:

5 $$\begin{array}{r}\nabla \cdot \vec{{{\bf\mathcal{E}}}}=4\pi \rho ,\,\,\,\nabla \cdot \vec{{{\bf\mathcal{H}}}}=0,\,\,\,\nabla \times\vec{{{\bf\mathcal{E}}}}=-\frac{1}{c}\,{\partial }_{\tau }^{* }\vec{{{\bf\mathcal{H}}}},\\ \nabla \times \vec{{{\bf\mathcal{H}}}}=\frac{1}{c}\,{\partial }_{\tau }^{* }\vec{{{\bf\mathcal{E}}}}+\frac{4\pi }{c}\vec{{{\bf\mathcal{J}}}}\end{array}$$

In the Hilbert space of analytic signals we now introduce the *complex Lorentz force* $\vec{{{\mathcal{F}}}}$ on a system, surrounded by vacuum, with densities of charge *ρ* and current $\vec{{{\mathcal{J}}}}$ occupying a volume *V*_0_ contained in *V*. This force $\vec{{{\mathcal{F}}}}$ should be identified with the complex source in the conservation of *complex linear mechanical momentum* **P**_*mech*_ ; viz.

6 $$\vec{{{\mathcal{F}}}}({{{\bf{r}}}},t,s)\equiv {\partial}_{t}{{{{\bf{P}}}}}_{mech}\,\,\,({{{\bf{r}}}},t,s)=\frac{1}{2}\displaystyle{\int}_{V}\,{d}^{3}r\,\left({\rho }^{*}\,\vec{{{\mathcal{E}}}}+\frac{1}{c}\vec{{{{\mathcal{J}}}}}^{*}\times\vec {{{\mathcal{B}}}}\right)$$

Which substituting ρ^*^ and $\vec{{{\mathcal{J}}}}$ through the Maxwell equations (5), leads to

7 $$\begin{array}{r}\vec{{{\mathcal{F}}}}({{{\bf{r}}}},s,t)\equiv {\partial }_{t}{{{{\bf{P}}}}}_{mech}=\frac{1}{8\pi }{\int}_{V}\,{d}^{3}r\,\left[\vec{{{\mathcal{E}}}}(\nabla \cdot \vec{{{{\mathcal{E}}}}}^{* })\right.\\ \left.+\vec{{{{\mathcal{B}}}}}^{* }(\nabla \cdot \vec{{{\mathcal{B}}}})-\vec{{{\mathcal{B}}}}\,\times \,(\nabla \times \vec{{{{\mathcal{B}}}}}^{* })-\frac{1}{c}\,({\partial }_{\tau }\,\vec{{{{\mathcal{E}}}}}^{* })\times \vec{{{\mathcal{B}}}}\right]\end{array}$$

We now recall (3) and (4) using the identities: ${\partial }_{\tau }\vec{{{{\mathcal{E}}}}}^{* }={\partial }_{t}\vec{{{{\mathcal{E}}}}}^{* }$ and ${\partial }_{\tau }^{* }\vec{{{\mathcal{B}}}}={\partial }_{t}\vec{{{\mathcal{B}}}}$. Then, $({\partial }_{\tau }\vec{{{{\mathcal{E}}}}}^{* })\times \vec{{{\mathcal{B}}}}={\partial }_{t}(\vec{{{{\mathcal{E}}}}}^{* }\times \vec{{{\mathcal{B}}}})-\vec{{{{\mathcal{E}}}}}^{* }\times ({\partial }_{t}\vec{{{\mathcal{B}}}})={\partial }_{t}(\vec{{{{\mathcal{E}}}}}^{* }\times \vec{{{\mathcal{B}}}})-\vec{{{{\mathcal{E}}}}}^{* }\times ({\partial }_{\tau }^{* }\vec{{{\mathcal{B}}}})$. Therefore using the third equation (5) we obtain:

8 $$\begin{array}{lll}\vec{{{\mathcal{F}}}}({{{\bf{r}}}},s,t)\,\equiv {\partial }_{t}{{{{\bf{P}}}}}_{mech}=\frac{1}{8\pi }{\int}_{V\,}{d}^{3}r\,\left[\vec{{{\mathcal{E}}}}(\nabla \cdot \vec{{{{\mathcal{E}}}}}^{* })+\vec{{{{\mathcal{B}}}}}^{* }(\nabla \cdot \vec{{{\mathcal{B}}}})\right.\\ \qquad\qquad\quad-\vec{{{\mathcal{B}}}}\times (\nabla \times \vec{{{{\mathcal{B}}}}}^{* })-{{\vec{\mathcal{{E}}}^{* }}}\times (\nabla \times \vec{{{\mathcal{E}}}}) \left.-\frac{1}{c}{\partial }_{t}(\vec{{{{\mathcal{E}}}}}^{* }\times \vec{{{\mathcal{B}}}})\right]\end{array}$$

Now, the scaled complex Poynting momentum is: $\vec{{{\mathcal{G}}}}({{{\bf{r}}}},t,s)=(1/{c}^{2})\vec{{{\mathcal{S}}}}({{{\bf{r}}}},t,s)=(1/8\pi c)[\vec{{{\mathcal{E}}}}({{{\bf{r}}}},t,s)\times \vec{{{{\mathcal{B}}}}}^{* }({{{\bf{r}}}},t,s)]$. Therefore, operating on the analytic signals in the four first terms of the integrand of (8), and from the identity: ${{{{\bf{a}}}}}^{* }\times (\nabla \times {{{\bf{a}}}})={a}_{j}^{* }{\partial }_{i}{a}_{j}-{a}_{j}^{* }{\partial }_{j}{a}_{i}$, (*I*, *j* = 1, 2, 3), we finally obtain for the complex Lorentz force the following *conservation equation* of the *scaled complex linear momentum*

9 $$\begin{array}{l}{{{{\mathcal{F}}}}}_{i}({{{\bf{r}}}},s,t)\equiv {\partial }_{t}{{{{{P}}}}}_{mech\,i}\,\,=-\displaystyle{\int}_{V}\,{d}^{3}r\,{\partial }_{t}{{{{\mathcal{G}}}}}_{i}^{* }+{\int}_{\partial V}\,{d}^{2}r{{{{\mathcal{T}}}}}_{ij}\,{n}_{j}\\ \qquad\qquad\qquad+\,\frac{{{{\rm{i}}}}}{8\pi }\displaystyle{\int}_{V}\,{d}^{3}r{{{\rm{Im}}}}[{{{{\mathcal{B}}}}}_{j}^{* }{\partial }_{i}{{{{\mathcal{B}}}}}_{j}-{{{{\mathcal{E}}}}}_{j}^{* }{\partial }_{i}{{{{\mathcal{E}}}}}_{j}]\end{array}$$

Where ${{{\rm{Im}}}}$ denotes imaginary part, *n*_*j*_ is the *j*th Cartesian component of the unit outward normal to the surface ∂*V* of *V*, and the scaled CMST is

10 $${{{{\mathcal{T}}}}}_{ij}\,({{{\bf{r}}}},s,t)=\frac{1}{8\pi }\left[{{{{\mathcal{E}}}}}_{i}{{{{\mathcal{E}}}}}_{j}^{* }+{{{{\mathcal{B}}}}}_{i}^{* }{{{{\mathcal{B}}}}}_{j}-\frac{1}{2}{\delta }_{ij}\,(| {{{\mathcal{E}}}}{| }^{2}+| {{{\mathcal{B}}}}{| }^{2})\right]$$

There is a remarkable appearence in (9) of the orbital (or canonical) momentum densities due to the electric and magnetic fields, [in this connection, we remark that after Belinfante, the terms orbital^25^ and canonical^26^ are indistinctly employed for $\vec{{{{\bf\mathcal{P}}}}}_{e}^{O}$, $\vec{{{{\bf\mathcal{P}}}}}_{m}^{O}$, and $\vec{{{{\bf\mathcal{P}}}}}^{O}=(1/2)(\vec{{{{\bf\mathcal{P}}}}}_{e}^{O}+ \vec{{{{\bf\mathcal{P}}}}}_{m}^{O})$^12,27–35^]]:

11 $$\begin{array}{l}{({{{{\mathcal{P}}}}}_{e}^{O})}_{i}\,({{{\bf{r}}}},s,t)=\frac{1}{8\pi \omega }\,\,{{{\rm{Im}}}}[{{{{\mathcal{E}}}}}_{j}^{* }{\partial }_{i}{{{{\mathcal{E}}}}}_{j}],\\ {({{{{\mathcal{P}}}}}_{m}^{O})}_{i}\,({{{\bf{r}}}},s,t)=\frac{1}{8\pi \omega }\,\,{{{\rm{Im}}}}[{{{{\mathcal{B}}}}}_{j}^{* }{\partial }_{i}{{{{\mathcal{B}}}}}_{j}],\,\,\,\,\,\,(\omega =kc)\end{array}$$

With which the conservation law (9) reads

12 $$\begin{array}{l}{\partial }_{t}[{{{{{P}}}}}_{mech\,i}\,\,\,+\displaystyle{\int}_{V}\,{d}^{3}r\,{{{{\mathcal{G}}}}}_{i}^{* }]={\int}_{\partial V}\,{d}^{2}r\,{{{{\mathcal{T}}}}}_{ij}\,{n}_{j}\\ \qquad\qquad\,\,+\,{{{\rm{i}}}}\omega \displaystyle{\int}_{V}{d}^{3}r{[\vec{{{{\mathcal{P}}}}}_{m}^{O}-\vec{{{{\mathcal{P}}}}}_{e}^{O}\,]}_{i}\end{array}$$

Equation (12) is one of the main results of this work. Its real and imaginary parts are

13 $${{{{\mathcal{F}}}}}_{i}^{R}({{{\bf{r}}}},t,s)\equiv {\partial }_{t}{{{{{P}}}}}_{mech\,i}^{R}\,\,=-{\int}_{V}\,{d}^{3}r\,{\partial}_{t}\,{{{{\mathcal{G}}}}}_{i}^{R}+{\int}_{\partial V}\,\,{d}^{2}r{{{{\mathcal{T}}}}}_{ij}^{R\,}{n}_{j}$$

14 $${{{{\mathcal{T}}}}}_{ij}^{R}=\frac{1}{8\pi }\,{{{\rm{Re}}}}\left\{{{{{\mathcal{E}}}}}_{i}{{{{\mathcal{E}}}}}_{j}^{* }+{{{{\mathcal{B}}}}}_{i}^{* }{{{{\mathcal{B}}}}}_{j}-\frac{1}{2}{\delta }_{ij}\,(| {{{\mathcal{E}}}}{| }^{2}+| {{{\mathcal{B}}}}{| }^{2})\right\}$$

and

15 $$\begin{array}{l}{{{{\mathcal{F}}}}}_{i}^{I}({{{\bf{r}}}},t,s)\equiv {\partial }_{t}{{{{{P}}}}}_{mech\,i}^{I}\,\,\,=\displaystyle{\int}_{V}\,{d}^{3}r\,{\partial }_{t}\,{{{{\mathcal{G}}}}}_{i}^{I}+{\int}_{\partial V}{d}^{2}r\,{{{{\mathcal{T}}}}}_{ij}^{I}{n}_{j}\\ \qquad\qquad\quad\,\,\,\,\,+\,\omega \displaystyle{\int}_{V}\,{d}^{3}r\,{[\vec{{{{\mathcal{P}}}}}_{m}^{O}-\vec{{{{\mathcal{P}}}}}_{e}^{O}\,]}_{i}\end{array}$$

with the scaled *imaginary*, or *reactive, Maxwell stress tensor* (IMST) ${{{{\mathcal{T}}}}}_{ij}^{I}$:

16 $${{{{\mathcal{T}}}}}_{ij}^{I}({{{\bf{r}}}},t,s)=\frac{1}{8\pi }{{{\rm{Im}}}}[{{{{\mathcal{E}}}}}_{i}{{{{\mathcal{E}}}}}_{j}^{* }+{{{{\mathcal{B}}}}}_{i}^{* }{{{{\mathcal{B}}}}}_{j}]$$

The superscripts *R* and *I* denote real and imaginary parts. It should be reminded that, although only explicitely written in the extreme left, all quantities in the above equations are functions of **r**, *t* and *s*. However, since there are no *s*-derivatives in (12)–(15), they also hold in the limit *s* → 0 and have physical meaning even if they are not scaled, then becoming instantaneous ones. This is in contrast with the energy in the complex Poynting vector theorem^23^. While the reactive power is determined in volt-ampere reactive (var), we note that the ILF is measured in newtons. Thus when *s* → 0 Eq. (14) becomes the familiar time-dependent conservation equation, in terms of analytic signals, for the time variation of instantaneous linear momentum ${{{{\bf{P}}}}}_{mech}^{R}\,\,\,({{{\bf{r}}}},t)$.

Nonetheless, the novel Eq. (15) represents a quite different process: the interaction wave-object yields a change of an additional scaled linear momentum ${{{{\bf{P}}}}}_{mech}^{I}\,\,\,({{{\bf{r}}}},t,s)$, giving rise to an *imaginary Lorentz force* (ILF) $\vec{{{{\mathcal{F}}}}}^{I}({{{\bf{r}}}},t,s)$ on the body to which a time change of reactive momentum of the field is substracted, irrespective of whether or not the incident wave possess it (like e.g., an evanescent or a two-wave interference field); so that even if the incident wave has no IPM, like propagating plane waves and beams, it arises in the scattering process. We shall insist on this below.

The ILF also stems from the flux, given by ${{{{\mathcal{T}}}}}_{ij}^{I}$, into the volume *V* that surrounds the object, of $\omega {\int}_{V}\,{d}^{3}r\,[\vec{{{{\mathcal{P}}}}}_{m}^{O}-\vec{{{{\mathcal{P}}}}}_{e}^{O}\,]$ given by the difference between the magnetic and electric orbital (or canonical) momenta (11) of the field, and that we define as the *reactive strength of orbital (or canonical) momentum* (ROM) stored in *V*. (We wish to remark the difference of the ROM concept with that of reactive orbital momentum that we introduced in ref. ^12^, Eq. (26), as the difference between the imaginary parts of the magnetic and electric orbital momenta, to be seen later on).

Since Eq. (13) also holds for *s* → 0, it rules instantaneous quantities too. Then, as we shall show below, ${\int}_{\partial V}\,{d}^{2}r\,{{{{\mathcal{T}}}}}_{ij}^{I}{n}_{j}$ for monochromatic fields is associated to an alternating flux of ROM, flowing from the body and returning to it.

For these reasons, we call $\vec{{{{\mathcal{F}}}}}^{I}$ the *reactive force* on the object of volume *V*_0_. These quantities, and $\vec{{{{\mathcal{F}}}}}^{I}$ in particular, have zero time-average, (like the reactive Poynting vector associated to the alternating flow of reactive power), in contrast with the *active force* $\vec{{{{\mathcal{F}}}}}^{R}$ which constitutes the time-averaged force sensed by the object, and observed in most experimental observations. Like in circuit theory^23,36,37^, as *s* diminishes there is an increase of modes of the standing flow going back and forth from *V*_0_, which corresponds to the ROM. The size *s* of the Cauchy filter *C*_*s*_(*t*) in (2), goes from *t* = *∞* to *t* = *minimum* *time* *scale*^23^ which in our case is 0, at which no more modes linked to the bouncing flow associated to the IMST, appear.

The arise of the canonical momenta in the IMST law (15), defining the ROM, is quite illuminating since it highlights the prominent role of these canonical momenta, (rather than of the Poynting momentum) in the generation of optical forces^12,28,31,38^. This will further be discussed below.

Therefore we have obtained in (15) a fundamental law for the instantaneous (*s* → 0) — and also for the scaled *s* ≠ 0 — force that confers another physical property to the IPM, $\vec{{{{\mathcal{G}}}}}^{I}$, in the realm of momentum conservation in light-matter interactions, which transcends that previously found in connection with self-forces on magnetoelectric particles^12,38,39^, assigned to specific illuminating fields, like e.g., evanescent, standing, and cylindrical vector beams^28,29,40^.

This exchange of imaginary momentum produces the reactive flow IMST and ILF, (therefore it should be instantaneously observed with a time-varying incident field, like e.g., an ultrashort pulse), which gives rise to an accretion of *reactive strengtht of canonical momentum*, ROM, around the body. As such, this ROM is not associated to the scattered field radiated into the far-zone, i.e., to the “radiated” RMST, but to reactive power, and thus it remains inside and in the near-field of the object volume *V*_0_. In this regard, we remark that this field imaginary momentum exchange is opposite to that of field (i.e., Poynting) time-averaged momentum, thus substracting, rather than adding, to the variation of imaginary linear momentum. This means that although the light wave loses field momentum $\vec{{{{\mathcal{G}}}}}^{R}$ on interaction, it may gain reactive field momentum $\vec{{{{\mathcal{G}}}}}^{I}$, even when it does not exist in the illuminating field. This is the case of e.g., an incident plane wave, discussed later on.

As a consequence, we may expect the ROM, and thus the flow related to the IMST and ILF, to severely affect the flow given by the RMST into the far-zone, namely, the standard time-averaged electromagnetic optical force, RLF, sensed by the body. The ILF is, as seen in the next sections, characterized by the total ROM stored in and out the particle, and its flow; in analogy with the imaginary work in terms of the reactive energy in and around the particle and its flow: the reactive momentum IPM^12^.

In this context, while the time-averaged force, i.e., the RLF, is the flow whose density is the RMST “radiated” into the far zone, the reactive force, ILF, is the stored ROM inside and in the near-field due to the flow IMST. Thus the complex flow, CMST, into the far and near zones constitutes a novel way of understanding the significance of both the standard RLF as well as the ILF. Hence the stored ROM will constitute a hindrance to the time-averaged force, RLF, sensed by the particle. As we shall see, the external stored ROM is due to the interference of the incident and scattered fields.

**Fig. 1:**
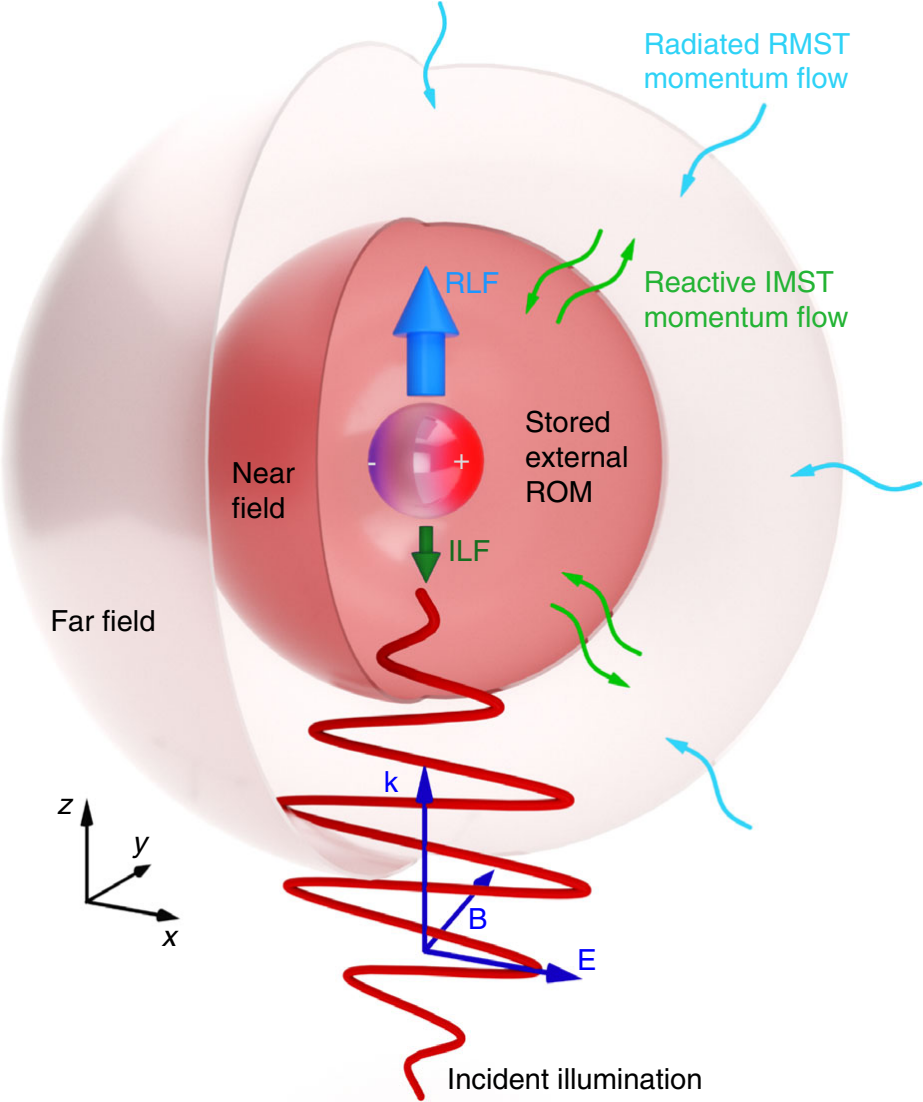
Outline of the physical process, (the near-field region is enlarged and the radiative area has been shrunk to ease reading). When an electromagnetic wave, (for example a monochromatic plane wave, as illustrated here), impinges on a body, charges are separated, thus inducing multipoles. The object feels a time-averaged Lorentz force, RLF, associated to the field (i.e., Poynting) momentum flux, RMST, flowing into a far-field surface, (e.g., spherical as shown). However, there also appears a flow, IMST, of imaginary field momentum, related to momentum flux going back and forth across a near-field surface, with zero time-average, which accumulates reactive strength of orbital momentum, ROM, stored both inside and in the near-field of the object, yielding a reactive force, ILF, on the body

Figure 1 that we shall address at the end of next section depicts this process for monochromatic fields for which it is more easily illustrated. Let us see next the CMST for these fields.

## Time-harmonic fields. The imaginary Maxwell stress tensor and the reactive force in the space-frequency domain

### Complex stress tensor and reactive strength of orbital momentum

For the spatial parts of time-harmonic fields whose analytic signals are $\vec{{{\mathcal{E}}}}({{{\bf{r}}}},\tau )={{{\bf{E}}}}({{{\bf{r}}}})\exp (-{{{\rm{i}}}}\omega t)$, $\vec{{{\mathcal{B}}}}({{{\bf{r}}}},\tau )={{{\bf{B}}}}({{{\bf{r}}}})\exp (-{{{\rm{i}}}}\omega t)$ and $\vec{{{\mathcal{J}}}}({{{\bf{r}}}},\tau )={{{\bf{J}}}}({{{\bf{r}}}})\exp (-{{{\rm{i}}}}\omega t)$, (*τ* = *t*), (*ω* = *kc*), Eq. (12) becomes

17 $${{{{\mathcal{F}}}}}_{i}={\int}_{\!\partial{V}}\,\,\,{d}^{2}r\,{T}_{ij}\,\,{n}_{j}+{{{\rm{i}}}}\omega {\int}_{\!V}{d}^{3}r\,{({{{{\bf{P}}}}}_{m}^{O}-{{{{\bf{P}}}}}_{e}^{O})}_{i}$$

which is the *complex Maxwell stress tensor theorem* in the space-frequency domain; the CMST being

18 $${T}_{ij}=\frac{1}{8\pi }\left[{E}_{i}{E}_{j}^{* }+{B}_{i}^{* }{B}_{j}-\frac{1}{2}{\delta }_{ij}\,(| {{{\bf{E}}}}{| }^{2}+| {{{\bf{B}}}}{| }^{2})\right]$$

With the electric and magnetic canonical momentum densities:

19 $${({{{{\bf{P}}}}}_{e}^{O})}_{i}=\frac{1}{8\pi \omega }{{{\rm{Im}}}}[{E}_{j}^{* }{\partial }_{i}{E}_{j}],\,\,\,{({{{{\bf{P}}}}}_{m}^{O})}_{i}=\frac{1}{8\pi \omega }{{{\rm{Im}}}}[{B}_{j}^{* }{\partial }_{i}{B}_{j}]$$

The real part of (17) is the well-known conservation law of linear momentum for monochromatic fields^6^:

20 $$\begin{array}{l} < \frac{d{{{{\bf{P}}}}}_{mech}}{dt}\,\,\, > \equiv < {{{{\mathcal{F}}}}}_{i} > \equiv {{{\rm{Re}}}}{\{{{{\mathcal{F}}}}}_{i}\} \\ \equiv\frac{1}{2}\displaystyle{\int}_{V}{{{\rm{Re}}}}{\left[{\rho }^{* }{{{\bf{E}}}}({{{\bf{r}}}})+\frac{{{{\bf{{J}}}^{* }}}}{c}\times {{{\bf{B}}}}({{{\bf{r}}}})\right]}_{i}\,{d}^{3}r =\displaystyle{\int}_{\partial V}{d}^{2}r\, < {T}_{ij} > {n}_{j}\,,\\ < {T}_{ij} > ={T}_{ij}^{R} =\frac{1}{8\pi }\,{{{\rm{Re}}}}\{{E}_{i}{E}_{j}^{* }+{B}_{i}^{* }{B}_{j}-\frac{1}{2}{\delta }_{ij\,}(| {{{\boldsymbol{E}}}}{| }^{2}+| {{{\boldsymbol{B}}}}{| }^{2})\}\end{array}$$

which expresses the total time-averaged electromagnetic optical force, < **F** > , on the system of charges and currents of the object of volume *V*_0_ contained in the integration volume *V*.

On the other hand, the imaginary part of (17) reads:

21 $$\begin{array}{lll}{{{{\mathcal{F}}}}}_{i}^{I}\,\equiv\, \frac{1}{2}\displaystyle{\int}_{V}{{{\rm{Im}}}}{\left[{\rho }^{* }{{{\bf{E}}}}({{{\bf{r}}}})+\frac{{{{\bf{{J}}}^{* }}}}{c}\times {{{\bf{B}}}}({{{\bf{r}}}})\right]}_{i}\,{d}^{3}r\\ \quad\,\,\,\,\,=\,\displaystyle{\int}_{\partial V}{d}^{2}r\,{T}_{ij}^{I}({{{\bf{r}}}}){n}_{j}+\omega {\int}_{V}{d}^{3}r\,{({{{{\bf{P}}}}}_{m}^{O}-{{{{\bf{P}}}}}_{e}^{O})}_{i}\end{array}$$

From (18) the imaginary, or reactive, Maxwell stress tensor (IMST) is:

22 $${T}_{ij}^{I}=\frac{1}{8\pi }{{{\rm{Im}}}}\{{E}_{i}{E}_{j}^{* }+{B}_{i}^{* }{B}_{j}\}$$

The first term of the right side of Eq. (21) expresses ${T}_{ij}^{I}$ as the flow density IMST into *V* across its surface ∂*V*. Because of this, ${T}_{ij}^{I}$ is a reactive quantity. Furthermore, as stated above, the second term is the ROM *stored* in *V*, namely in and around the body volume *V*_0_. Then, the *reactive Lorentz force* (ILF), $\vec{{{{\mathcal{F}}}}}^{I}$, is the here uncovered optical force, acting on the object, related to the alternating instantaneous one, as a result of the inward flow IMST ${T}_{ij}^{I}$ and accretion of reactive strength of orbital momentum, $\omega {\int}_{V}{d}^{3}r\,({{{{\bf{P}}}}}_{m}^{O}-{{{{\bf{P}}}}}_{e}^{O})$, in and around the object volume *V*_0_.

Analogously, while Eq. (20) yields the time-averaged optical torque < **Γ** > on the object, of lever arm **r**: ${{{\bf{r}}}}\times \vec{{{{\mathcal{F}}}}}^{R}$^41–43^, Eq. (21) leads to the *reactive torque*: ${{{{\mathbf{\Xi }}}}}^{I}={{{\bf{r}}}}\,\times\, {{{\vec{\mathcal{F}}}}}^{I}$, [see Appendix A of the Supp. Mat.].

### The instantaneous Maxwell stress tensor

At this point it is convenient to introduce the instantaneous Maxwell stress tensor, that gives rise to the alternating instantaneous ILF, built by the fields $\vec{\mathfrak{E}}({{{\bf{r}}}},t)={{{\rm{Re}}}}\{\vec{{{\mathcal{E}}}}({{{\bf{r}}}},t)\}$ and $\vec{\mathfrak{B}}({{{\bf{r}}}},t)={{{\rm{Re}}}}\{\vec{{{\mathcal{B}}}}({{{\bf{r}}}},t)\}$. Like for other instantaneous time-harmonic quantities^12,44,45^, it is immediate to obtain:

$$\begin{array}{ll}{{\mathfrak{T}}}_{ij}({{{\bf{r}}}},t)&=\frac{1}{4\pi }\left\{{{\mathfrak{E}}}_{i}({{{\bf{r}}}},t){{\mathfrak{E}}}_{j}({{{\bf{r}}}},t)+{{\mathfrak{B}}}_{i}({{{\bf{r}}}},t){{\mathfrak{B}}}_{j}({{{\bf{r}}}},t)\right.\\ &\quad\,\, \left.-\frac{1}{2}{\delta }_{ij}[{{\mathfrak{E}}}^{2}({{{\bf{r}}}},t)+{{\mathfrak{B}}}^{2}({{{\bf{r}}}},t)]\right\}\\&=\, < {T}_{ij}({{{\bf{r}}}}) > +\frac{1}{8\pi }{{{\rm{Re}}}}\left\{\left[{E}_{i}({{{\bf{r}}}}){E}_{j}({{{\bf{r}}}})+{B}_{i}({{{\bf{r}}}}){B}_{j}({{{\bf{r}}}})\right.\right.\\&\quad\,\, \left.-\frac{1}{2}{\delta }_{ij}[{{{\boldsymbol{E}}}}({{{\bf{r}}}})\cdot {{{\boldsymbol{E}}}}({{{\bf{r}}}})+{{{\boldsymbol{B}}}}({{{\bf{r}}}})\cdot {{{\boldsymbol{B}}}}({{{\bf{r}}}})]\exp (-2{{{\rm{i}}}}\omega t)\right\}\end{array}$$

However by a simple calculation we gain insight expressing it as:

23 $$\begin{array}{ll}{{\mathfrak{T}}}_{ij}({{{\bf{r}}}},t)&= \,< {T}_{ij}({{{\bf{r}}}}) \,>\, (1+\cos 2\omega t)+{T}_{ij}^{I}({{{\bf{r}}}})\sin 2\omega t\\ &\quad\,+\,\frac{1}{4\pi }\left\{{E}_{i}^{R}({{{\bf{r}}}}){E}_{j}^{I}({{{\bf{r}}}})+{B}_{i}^{I}({{{\bf{r}}}}){B}_{j}^{R}({{{\bf{r}}}})\right.\\ &\quad\,\left.-\,\frac{1}{2}{\delta }_{ij}[{{{{\boldsymbol{E}}}}}^{R}({{{\bf{r}}}})\cdot {{{{\boldsymbol{E}}}}}^{I}({{{\bf{r}}}})+{{{{\boldsymbol{B}}}}}^{I}({{{\bf{r}}}})\cdot {{{{\boldsymbol{B}}}}}^{R}({{{\bf{r}}}})]\right\}\sin 2\omega t\\ &\quad\,-\,\frac{1}{4\pi }\left\{{E}_{i}^{I}({{{\bf{r}}}}){E}_{j}^{I}({{{\bf{r}}}})+{B}_{i}^{I}({{{\bf{r}}}}){B}_{j}^{I}({{{\bf{r}}}})\right.\\ &\quad\,\left.-\frac{1}{2}{\delta }_{ij}[{{{{\boldsymbol{E}}}}}^{I}({{{\bf{r}}}})\cdot {{{{\boldsymbol{E}}}}}^{I}({{{\bf{r}}}})+{{{{\boldsymbol{B}}}}}^{I}({{{\bf{r}}}})\cdot {{{{\boldsymbol{B}}}}}^{I}({{{\bf{r}}}})]\right\}\cos 2\omega t\end{array}$$

While the term with <*T*_*ij*_(**r**)> does not change sign with time, as expected from the instantaneous MST part associated with the time-averaged flow of momentum, the term containing the reactive Maxwell stress tensor, ${T}_{ij}^{I}({{{\bf{r}}}})$, alternates its sign at frequency 2*ω* following the variation of $\sin 2\omega t$. This is in accordance with the interpretation of the imaginary part of (17) being related to the ROM flux, i.e., the IMST, going back and forth from the object volume *V*_0_ with zero time-average. (Note that the Fourier decomposition (1) into time-harmonic components shows this zero time-average of the ROM flow also for general time-varying fields). In addition, there is an alternating generally non-zero contribution to this instantaneous flow ${{\mathfrak{T}}}_{ij}({{{\bf{r}}}},t)$ in the terms within the curly brackets of (23). Obviously only the <*T*_*ij*_(**r**)> term remains on time-averaging in (23).

### The external reactive strength of orbital momentum

One may obtain additional discernment on the role played by the terms of the conservation law (21). Let us take the volume *V* to be *V*_*∞*_ of a large sphere of radius *r* such that *kr* → *∞*. The flow IMST across its surface ∂*V*_*∞*_ is zero since the CMST is real in the far zone^38^, (this may also be easily seen considering the far zone expression of the scattered fields. Then only the diagonal elements of the CMST contribute to the Lorentz force and, as such, this reduces to a real quantity; namely, only $<\vec {{{\mathcal{F}}}} >$ is obtained in this region). Then in the volume *V*_0_ of the scattering body within *V* we have

24 $$\begin{array}{lll}\vec{{{{\mathcal{F}}}}}^{I}&\equiv& \frac{1}{2}\displaystyle{\int}_{{V}_{0}}{{{\rm{Im}}}}\left[{\rho }^{* }{{{\bf{E}}}}({{{\bf{r}}}})+\frac{{{{\bf{{J}}}^{* }}}}{c}\times {{{\bf{B}}}}({{{\bf{r}}}})\right]\,{d}^{3}r\\&=& \omega \displaystyle{\int}_{{V}_{\infty }}{d}^{3}r\,({{{{\bf{P}}}}}_{m}^{O}-{{{{\bf{P}}}}}_{e}^{O})\end{array}$$

Equation (24) shows that the ILF, i.e., the source term in the left side of (21), is given by the overall ROM. In addition, introducing (24) into (21), we obtain

25 $${\int}_{\!\partial V}{d}^{2}r\,{T}_{ij}^{I}{n}_{j}=\omega {\int}_{\!{V}_{\infty }-V}{d}^{3}r\,{({{{{\bf{P}}}}}_{m}^{O}-{{{{\bf{P}}}}}_{e}^{O})}_{i}$$

Equation (25) is important because it illustrates that the part of the ILF due to the flow IMST into the volume *V* surrounding the body volume *V*_0_, is given by the total outside ROM stored between *V*_*∞*_ and *V*. In particular, *V* may approach the source volume *V*_0_. We also see from (25) that in the far-field (*FF*) region: ${{{{\bf{P}}}}}_{m}^{O\,FF}={{{{\bf{P}}}}}_{e}^{O\,FF}$ since the right side of (25) is zero as *V* approaches *V*_*∞*_.

Now we are in position to have the overall perspective of the CMST theorem in both the far-field and near-field regions, and to understand how the flow IMST builds up the ROM inside and outside the particle, with the ILF being generated. Figure 1 illustrates the process. It should be recalled that the illuminated object behaves like an antenna, so that the interaction also conveys a reactive work on the charges, building-up reactive power which hinders the efficiency of radiated scattered energy^1,12,19^. As we shall see in the section on dipolar objects, the ILF and stored ROM (which are the dynamical analogues of the reactive work and reactive power, respectively) counteract against the RLF, (i.e., the dynamical analogue of the radiated energy). In consequence, a large ROM, and hence a strong ILF, conveys a decrease of "radiative" RMST, i.e., of RLF. Conversely, low ROM and ILF are linked to a larger RLF.

## The canonical and spin momenta and the complex Lorentz force

In this section, we establish a connection of the real and imaginary parts of the canonical and spin momenta and of the complex Lorentz force introduced above.

### The imaginary Lorentz force and the spin momenta. The reactive strength of Poynting momentum. Implications for the reactive Maxwell stress tensor

The ILF $\vec{{{{\mathcal{F}}}}}^{I}$ may be expressed by writing the flow IMST in terms of the real electric and magnetic spin (i.e., Belinfante) momenta, which is of conceptual interest. This is obtained using the identity: ${{{{\bf{a}}}}}^{* }\times (\nabla \times {{{\bf{a}}}})={a}_{j}^{* }{\partial }_{i}{a}_{j}-{a}_{j}^{* }{\partial }_{j}{a}_{i}$, from which we have: ${{{\rm{Im}}}}\{-{{{{\bf{a}}}}}^{* }\times (\nabla \times {{{\bf{a}}}})+{{{\bf{a}}}}(\nabla \cdot {{{{\bf{a}}}}}^{* })\}={{{\rm{Im}}}}\{{\partial }_{j}({a}_{i}{a}_{j}^{* })-{a}_{j}^{* }{\partial }_{i}{a}_{j}\}$. On the other hand, since the Levi-Civita tensor holds: *ϵ*_*ijk*_*ϵ*_*klm*_ = *δ*_*il*_*δ*_*jm*_ − *δ*_*im*_*δ*_*jl*_, we have $(1/2)\nabla \times {{{\rm{Im}}}}\{{{{{\bf{a}}}}}^{* }\times {{{\bf{a}}}}\}={{{\rm{Im}}}}\{{\partial }_{j}({a}_{i}^{* }{a}_{j})\}$.

Hence, using the above identity, we may write these spin momentum densities^12,28^ as:

26 $$\begin{array}{r}{{{{\bf{P}}}}}_{e}^{S}=\frac{1}{16\pi \omega }\nabla \times {{{\rm{Im}}}}\{{{{{\bf{E}}}}}^{* }\times {{{\bf{E}}}}\}=\frac{1}{8\pi \omega }{{{\rm{Im}}}}\{{\partial }_{j}({E}_{i}^{* }{E}_{j})\},\\ {{{{\bf{P}}}}}_{m}^{S}=\frac{1}{16\pi \omega }\nabla \times {{{\rm{Im}}}}\{{{{{\bf{B}}}}}^{* }\times {{{\bf{B}}}}\}=\frac{1}{8\pi \omega }{{{\rm{Im}}}}\{{\partial }_{j}({B}_{i}^{* }{B}_{j})\}\end{array}$$

Therefore the ILF, Eq. (21), reads

27 $$\begin{array}{ll}{{{{\mathcal{F}}}}}_{i}^{I}&=\frac{1}{8\pi }\displaystyle{\int}_{V}{{{\rm{Im}}}}\left[{{{\bf{E}}}}(\nabla \cdot {{{{\bf{E}}}}}^{* })+{{{{\bf{B}}}}}^{* }(\nabla \cdot {{{\bf{B}}}})-{{{{\bf{E}}}}}^{* }\times (\nabla \times {{{\bf{E}}}})\right.\\&\quad {\left.-\,{{{\bf{B}}}}\times (\nabla \times {{{{\bf{B}}}}}^{* })\right]}_{i}\,{d}^{3}r=\frac{1}{8\pi }\displaystyle{\int}_{V}{{{\rm{Im}}}}\left[-{\partial }_{j}({E}_{i}^{* }{E}_{j})\right.\\ &\quad\left.-\,{E}_{j}^{* }{\partial }_{i}{E}_{j}+{\partial }_{j}({B}_{i}^{* }{B}_{j}^{* })+{B}_{j}^{* }{\partial }_{i}{B}_{j}\right]{d}^{3}r\end{array}$$

which finally becomes

28 $$\vec{{{{\mathcal{F}}}}}^{I}=\omega {\int}_{V}{d}^{3}r[({{{{\bf{P}}}}}_{m}^{S}-{{{{\bf{P}}}}}_{e}^{S})+({{{{\bf{P}}}}}_{m}^{O}-{{{{\bf{P}}}}}_{e}^{O})]$$

Equation (28), which is other of the main results of this work, provides a physical meaning of the ILF in terms of the spin and orbital momenta as a *reactive strength of Poynting momentum* whose density is : $\omega [({{{{\bf{P}}}}}_{m}^{O}+{{{{\bf{P}}}}}_{m}^{S})-({{{{\bf{P}}}}}_{e}^{O}+{{{{\bf{P}}}}}_{e}^{S})]$. And

29 $$\begin{array}{lll}\displaystyle{\int}_{\partial V}{d}^{2}r\,{T}_{ij}^{I}{n}_{j}\,=\,\omega \displaystyle{\int}_{V}{d}^{3}r{({{{{\bf{P}}}}}_{m}^{S}-{{{{\bf{P}}}}}_{e}^{S})}_{i}\\ {{{\rm{or}}\; :}}\,\,\,\,\nabla_j \cdot {T}_{ij}^{I}\,=\,\omega {({{{{\bf{P}}}}}_{m}^{S}-{{{{\bf{P}}}}}_{e}^{S})}_{i}\end{array}$$

I.e. the *reactive strength, of spin momentum* (RSM), $\omega {\int}_{V}{d}^{3}r({{{{\bf{P}}}}}_{m}^{S}-{{{{\bf{P}}}}}_{e}^{S})$, in *V* is equivalent to the incoming flow IMST across ∂*V*.

In addition, from Eqs. (25) and (29) we have:

30 $${\int}_{\!V}{d}^{3}r\,({{{{\bf{P}}}}}_{m}^{S}-{{{{\bf{P}}}}}_{e}^{S})={\int}_{\!{V}_{\infty }-V}{d}^{3}r\,({{{{\bf{P}}}}}_{m}^{O}-{{{{\bf{P}}}}}_{e}^{O})$$

And thus we obtain that as *V* approaches *V*_*∞*_, one has that

31 $${\int}_{\!{V}_{\infty }}{d}^{3}r\,({{{{\bf{P}}}}}_{m}^{S}-{{{{\bf{P}}}}}_{e}^{S})=0$$

We emphasize that when the emitter of volume *V*_0_ ⊆ V is a scatterer, the above equations hold for the total field given by the sum of the incident and scattered fields.

*Equation* (30) *is an important novel balance law for the formation of RSM in a finite volume V that contains the body volume* *V*_0_*, as it equals the accumulation of ROM in the whole space outside V, and so it builds the ILF*. In particular, it holds when *V* → *V*_0_. On the other hand, as regards (31) we shall later show that the overall spin momentum of the field emitted, or scattered, by a dipolar particle is zero.

In particular, in free space and for surface waves, one knows that ${\int}_{{V}_{\infty }}{d}^{3}r\,{{{{\bf{P}}}}}^{S}=(1/2){\int}_{{V}_{\infty }}{d}^{3}r\,({{{{\bf{P}}}}}_{m}^{S}+{{{{\bf{P}}}}}_{e}^{S})=0$^28,30^, therefore (31) will imply that in the whole space *V*_*∞*_ the overall electric and magnetic spin momenta of a free-field are zero, and thus according to (30) this amounts to *V* = 0. This is compatible with the evidence that in this case, no scattering object is present.

### The time-averaged Poynting momentum with sources and the imaginary Lorentz force

At this point, we must remark the compatibility of the ILF, Eq. (28), with the formulation of the (real) Poynting momentum with sources.

Let us, first, employ the third Maxwell equation to eliminate **B** in the definition of the time-averaged Poynting momentum $< {{{\bf{g}}}} > = < {{{\bf{S}}}} > /{c}^{2}=(1/8\pi c){{{\rm{Re}}}}\{{{{\bf{E}}}}\times {{{{\bf{B}}}}}^{* }\}$:

32 $$\begin{array}{ll} < {{{\bf{g}}}} > \,=\,{{{{\bf{P}}}}}_{e}^{S}+{{{{\bf{P}}}}}_{e}^{O}+\frac{1}{8\pi \omega }{{{\rm{Im}}}}\{{{{\bf{E}}}}(\nabla \cdot {{{{\bf{E}}}}}^{* })\}\\ \qquad\,\,\,\,\,= {{{{\bf{P}}}}}_{e}^{S}+{{{{\bf{P}}}}}_{e}^{O}+\frac{1}{2\omega }{{{\rm{Im}}}}\{{\rho }^{* }{{{\bf{E}}}}\}\end{array}$$

Correspondingly, by eliminating **E** in the definition of < **g** > , we obtain in terms of the magnetic momenta:

33 $$\begin{array}{ll} < {{{\bf{g}}}} > \,=\,-\,\frac{1}{2c\omega }{{{\rm{Im}}}}\{{{{{\bf{J}}}}}^{* }\times {{{\bf{B}}}}\}+{{{{\bf{P}}}}}_{m}^{S}+{{{{\bf{P}}}}}_{m}^{O}\\ \qquad\qquad\,+\,\frac{1}{8\pi \omega }{{{\rm{Im}}}}\{{{{\bf{B}}}}(\nabla \cdot {{{{\bf{B}}}}}^{* })\}\\ \qquad\,\,\,\,\,= {{{{\bf{P}}}}}_{m}^{S}+{{{{\bf{P}}}}}_{m}^{O}-\frac{1}{2c\omega }{{{\rm{Im}}}}\{{{{{\bf{J}}}}}^{* }\times {{{\bf{B}}}}\}\end{array}$$

Adding (32) and (33) we get an expression for the time-averaged field momentum valid in a space which is not source-free, but that contains charge and current distributions:

34 $$< {{{\bf{g}}}} > ={{{{\bf{P}}}}}^{S}+{{{{\bf{P}}}}}^{O}+\frac{1}{4\omega }{{{\rm{Im}}}}\left\{{\rho }^{* }{{{\bf{E}}}}-\frac{1}{c}{{{{\bf{J}}}}}^{* }\times {{{\bf{B}}}}\right\}$$

where we have employed the (real, i.e., time-averaged) canonical and spin momenta^12,28,30^:

35 $${{{{\bf{P}}}}}^{O}=\frac{1}{2}({{{{\bf{P}}}}}_{e}^{O}+{{{{\bf{P}}}}}_{m}^{O}),\,\,\,\,\,\,{{{{\bf{P}}}}}^{S}=\frac{1}{2}({{{{\bf{P}}}}}_{e}^{S}+{{{{\bf{P}}}}}_{m}^{S})$$

In free-space (34) turns into the well-known decomposition of <**g**> as the sum of the spin and canonical momentum densities. In Appendix B of Supp. Mat. we show the derivation of (32) and (33) from first Lagrangian principles.

*Note the compatibility of* (28) *with* (32) *and* (33), *since substracting* (32) *from* (33), *and integrating in V, we immediately obtain* (28).

### The imaginary field momentum with sources: The reactive orbital and spin momenta. The time-averaged force

The above time-averaged spin and canonical momenta are the real parts of complex canonical and spin momenta^12,28^: ${\tilde{{{{\bf{P}}}}}}^{O}={{{{\bf{P}}}}}^{O}+{{{\rm{i}}}}{{{{\bf{P}}}}}^{OI}$ and ${\tilde{{{{\bf{P}}}}}}^{S}={{{{\bf{P}}}}}^{S}+{{{\rm{i}}}}{{{{\bf{P}}}}}^{SI}$.

The imaginary parts, **P**^*O* *I*^ and **P**^*S* *I*^, play an important role complementary to that of the above real parts, namely in the time-averaged force. To see it, we now proceed with the reactive Poynting momentum ${{{{\bf{g}}}}}^{I}=(1/8\pi c){{{\rm{Im}}}}\{{{{\bf{E}}}}\times {{{{\bf{B}}}}}^{* }\}$ in a way analogous to that leading to Eqs. (32) and (33). It is straightforward to obtain:

36 $$\begin{array}{ll}{({{{{\bf{g}}}}}^{I})}_{i}\,=\,\frac{1}{8\pi \omega }\{-{{{\rm{Re}}}}[{\partial }_{j}({E}_{i}^{* }{E}_{j})]+\frac{1}{2}{\delta }_{ij}{\partial }_{j}| {{{\bf{E}}}}{| }^{2}\}\\ \qquad\quad\,\,+\,\frac{1}{2\omega }{{{\rm{Re}}}}{\{{\rho }^{* }{{{\bf{E}}}}\}}_{i}\end{array}$$

and

37 $$\begin{array}{ll}{({{{{\bf{g}}}}}^{I})}_{i}\,=\,\frac{1}{8\pi \omega }\{{{{\rm{Re}}}}[{\partial }_{j}({B}_{i}^{* }{B}_{j})]-\frac{1}{2}{\delta }_{ij}{\partial }_{j}| {{{\bf{B}}}}{| }^{2}\}\\ \qquad\quad\,\, -\frac{1}{2\omega }{{{\rm{Re}}}}{\{\frac{1}{c}{{{{\bf{J}}}}}^{* }\times {{{\bf{B}}}}\}}_{i}.\,\,\,\,\,(i,j=1,2,3)\end{array}$$

Substracting (36) from (37), we obtain the well-known time-averaged force density, [cf. Eq. (20)],

38 $$\begin{array}{ll}\frac{1}{2}{{{\rm{Re}}}}\{{\rho }^{\ast}{({{{\bf{E}}}})}_{i}+\frac{1}{c}{({{{{\bf{J}}}}}^{\ast}\times {{{\bf{B}}}})}_{i}\}\,=\,\frac{1}{8\pi }\left\{{\partial }_{j}{{{\rm{Re}}}}({E}_{i}{E}_{j}^{\ast }+{B}_{i}^{\ast}{B}_{j})\right.\\ \qquad\qquad\qquad\qquad\qquad\qquad\qquad\left.-\frac{1}{2}{\delta }_{ij}{\partial }_{j}(\vert {{{\bf{E}}}}{\vert}^{2}+\vert {{{\bf{B}}}}{\vert }^{2})\right\}\end{array}$$

On the other hand, adding (36) and (37), we derive a representation for the reactive Poynting momentum density:

39 $$\begin{array}{ll}{({{{{\bf{g}}}}}^{I})}_{i}\,=\,\frac{1}{8\pi \omega }{\partial }_{j}\{\frac{1}{2}{{{\rm{Re}}}}({B}_{i}^{* }{B}_{j}-{E}_{i}{E}_{j}^{* })-\frac{1}{4}{\delta }_{ij}(| {{{\bf{B}}}}{| }^{2}-| {{{\bf{E}}}}{| }^{2})\}\\ \qquad\quad\, \,+\,\frac{1}{4\omega }{{{\rm{Re}}}}{\{{\rho }^{* }{{{\bf{E}}}}-\frac{1}{c}{{{{\bf{J}}}}}^{* }\times {{{\bf{B}}}}\}}_{i}\end{array}$$

We now introduce the imaginary spin curl and orbital momenta, viz.:

40 $$\begin{array}{ll}{({{{{\bf{P}}}}}_{e}^{SI})}_{i}=\frac{1}{8\pi \omega }{{{\rm{Re}}}}\{{\partial }_{j}({E}_{i}^{* }{E}_{j})\},\,\,\\ {({{{{\bf{P}}}}}_{m}^{SI})}_{i}=\frac{1}{8\pi \omega }{{{\rm{Re}}}}\{{\partial }_{j}({B}_{i}^{* }{B}_{j})\},\\ {({{{{\bf{P}}}}}_{e}^{OI})}_{i}=-\frac{1}{8\pi \omega }{\partial }_{j}\frac{1}{2}{\delta }_{ij}| {{{\bf{E}}}}{| }^{2}=-\frac{1}{8\pi \omega }\frac{1}{2}{\partial }_{i}| {{{\bf{E}}}}{| }^{2},\\ {({{{{\bf{P}}}}}_{m}^{OI})}_{i}=-\frac{1}{8\pi \omega }\frac{1}{2}{\partial }_{i}| {{{\bf{B}}}}{| }^{2}\end{array}$$

We emphasize that the definition of the spin momenta as ∂_*j*_(⋅) in (40) [cf. also (26)] is useful as it permits a direct introduction of the CMST, as well as of the complex spin momenta, whose imaginary parts yield:

41 $${{{{\bf{P}}}}}^{SI}=\frac{1}{2}({{{{\bf{P}}}}}_{e}^{S\,I}+{{{{\bf{P}}}}}_{m}^{S\,I})\,;\,\,\,\,\,\,\,\,\,\,{{{{\bf{P}}}}}^{OI}=\frac{1}{2}({{{{\bf{P}}}}}_{e}^{O\,I}+{{{{\bf{P}}}}}_{m}^{O\,I})$$

and the *reactive spin* and *orbital momenta*^12^:

42 $${\vec{{{\mathcal{P}}}}}^{S}=\frac{1}{2}({{{{\bf{P}}}}}_{m}^{S\,I}-{{{{\bf{P}}}}}_{e}^{S\,I});\,\,\,\,\,\,\,\,\,\,\vec{{{{\mathcal{P}}}}}^{O}=\frac{1}{2}({{{{\bf{P}}}}}_{m}^{O\,I}-{{{{\bf{P}}}}}_{e}^{O\,I})$$

So that the imaginary spin and orbital momenta introduced in Eqs. (40) and (41) permit us to write the time-averaged force, (38), as

43 $$< \vec{{{\mathcal{F}}}} > =2\omega {\int}_{V}{d}^{3}r\,({{{{\bf{P}}}}}^{SI}+{{{{\bf{P}}}}}^{OI})$$

which is the desired *representation of the RLF in terms of the imaginary momenta*. While the imaginary Poynting momentum, (39), reads

44 $${{{{\bf{g}}}}}^{I}=\vec{{{{\mathcal{P}}}}}^{S}+\vec{{{{\mathcal{P}}}}}^{O}+\frac{1}{4kc}{{{\rm{Re}}}}\{{\rho }^{* }{{{\bf{E}}}}-\frac{1}{c}{{{{\bf{J}}}}}^{* }\times {{{\bf{B}}}}\}$$

Equation (44) is the *representation of* **g**^*I*^ *through the reactive spin and orbital momenta* in presence of sources; while (43) and (28) formulate the real and imaginary Lorentz forces in terms of the respective imaginary and real parts of the spin and canonical momenta. These momenta are therewith shown to be the ultimate dynamic quantities that characterize the complex optical force. We shall later make use of the fact that in a scattering configuration they correspond to the total fields; namely, incident plus scattered.

### Example 1: Evanescent wave

Let us consider the monochromatic reactive wavefield consisting of the evanescent wave created by total internal reflection at a plane interface *z* = 0 separating air in *z* ≥ 0 from a dielectric in the half-space *z* < 0. The plane of incidence being *OXZ*. The complex spatial parts of the electric and magnetic vectors in *z* ≥ 0, are expressed in a Cartesian coordinate basis $\{\hat{{{{\bf{x}}}}},\hat{{{{\bf{y}}}}},\hat{{{{\bf{z}}}}}\}$ as^12,46^:

45 $$\begin{array}{l}{{{\bf{E}}}}=\left(-\frac{{{{\rm{i}}}}q}{k}{T}_{\parallel },{T}_{\perp },\frac{K}{k}{T}_{\parallel }\right)\exp ({{{\rm{i}}}}Kx-qz),\\ {{{\bf{B}}}}=\left(-\frac{{{{\rm{i}}}}q}{k}{T}_{\perp },-{T}_{\parallel },\frac{K}{k}{T}_{\perp }\right)\exp ({{{\rm{i}}}}Kx-qz)\end{array}$$

For TE or *s* (TM or *p*) - polarization, i.e., **E** (**B**) perpendicular to the plane of incidence *OXZ*, only those components with the transmission coefficient *T*_⊥_, (*T*_∥_) are chosen. *K* denotes the component, parallel to the interface, of the wavevector **k** = (*K*, 0, i*q*), $q=\sqrt{{K}^{2}-{k}^{2}}$.

The densities of energy, *w* = *w*_*e*_ + *w*_*m*_, reactive power, *w*_*react*_ = 2*ω*(*w*_*m*_ − *w*_*e*_), Poynting momentum, spin, and canonical momentum of this wave in *Z* ≥ 0 are straightforwardly obtained from (45) and well-known^12,28^.

Since in this case^12^ **P**^*S* *I*^ = −**P**^*O* *I*^, the global time-averaged force density in *z* ≥ 0 due to the evanescent wave is, following (43), equal to zero. This is to be expected since no body exists in *z* ≥ 0; and such a force $< \vec{{{\bf\mathcal{F}}}} >$, Eq. (43), with *V* being the source-free space *z* ≥ 0, is [cf. Eq. (38)] also equal to the flow RMST on the surface ∂*V*. Since this flow is the same whatever the contour surrounding the sources is, we can take ∂*V* in the far-zone, (*kz* → *∞*), at which the evanescent wave is zero, and thus it does not contribute to this flux.

Furthermore, note that the evanescent wave CMST divergence is

46 $$\begin{array}{ll}\nabla \cdot {T}_{ij}={\partial }_{j}{T}_{ij}\,=\,{\partial }_{z}{T}_{i3}=[{{{\rm{i}}}}\frac{K{q}^{2}}{4\pi {k}^{2}}(| {T}_{\parallel }{| }^{2}-| {T}_{\perp }{| }^{2}),0,0]{e}^{-2qz}\\ \qquad\qquad\qquad\,\,\,\,=-{{{\rm{i}}}}\frac{K}{\omega }({w}_{react},0,0)\end{array}$$

The extreme right of (46) has been written in terms of *w*_*react*_^12^. The real part of (46) is zero, in agreement with the above statement. Nevertheless, the imaginary part is

47 $$\nabla \cdot {T}_{ij}^{I}={\partial }_{z}{T}_{i3}^{I}=\omega ({{{{\bf{P}}}}}_{m}^{S}-{{{{\bf{P}}}}}_{e}^{S})=-\frac{K}{\omega }({w}_{react},0,0)$$

which is associated [cf. Eq. (23)] to a back-and-forth flow of momentum in the *x*-direction of propagation of the evanescent wave, without any net transfer of momentum to any body in *z* ≥ 0. Besides, we know^12^ that

48 $$-\frac{K}{\omega }({w}_{react},0,0)=-\omega ({{{{\bf{P}}}}}_{m}^{O}-{{{{\bf{P}}}}}_{e}^{O})$$

Hence, replacing the first term of the right side of Eq. (21) by (47) and (48), we get for the reactive force density: $\frac{1}{2}{{{\rm{Im}}}}{[{\rho }^{* }{{{\bf{E}}}}({{{\bf{r}}}})+\frac{{{{\bf{{J}}}^{* }}}}{c}\times {{{\bf{B}}}}({{{\bf{r}}}})]}_{i}=0$, wich agrees with the absence of object in *z* ≥ 0.

In addition, like the canonical momentum, (cf. Eq. (35) and ref. ^12^): ${{{{\bf{P}}}}}^{O}=\frac{K}{\omega }\,(w\,,0\,,0)$, the flow $\nabla \cdot {T}_{ij}^{I}=\omega ({{{{\bf{P}}}}}_{m}^{S}-{{{{\bf{P}}}}}_{e}^{S})$ is superluminal, (*K* > *k*). However, while **P**^*O*^ has an *x*-component characterized by the electromagnetic energy density, *w*, the flow density $\nabla \cdot {T}_{ij}^{I}$ is, according to (47), governed by the reactive power, *w*_*react*_, of the evanescent wave.

## Near-field nature of the imaginary stress tensor

As discussed above, (see also Appendix D of Supp. Mat.), the flow IMST is zero in the far-zone because in that region only the diagonal part of the CMST contributes to this flow, and hence it is real. Next, we prove that only evanescent components exist in the flow IMST, thus showing its near-field nature as a reactive quantity^12^.

Using Eq. (29), we use for simplicity a framework such that the sources are in *z* < 0, and thus the integration is done on the *z* = *z*_0_ ≥ 0 plane. The electric field **E**(**r**) propagating into the half-space *z* ≥ 0 is represented by its angular spectrum of plane-wave components^12^. Using the subscripts *h* and *e* for homogeneous and evanescent components of complex amplitudes: electric, **e**(**K**), and magnetic, **b**(**K**), the integrated IMST divergence (29) per unit length on *z* is, (cf. Appendix C of Supp. Mat.):

49 $$\begin{array}{l}\omega \displaystyle\int\nolimits_{-\infty }^{\infty }{d}^{2}{{{\bf{R}}}}({{{{\bf{P}}}}}_{m}^{S}-{{{{\bf{P}}}}}_{e}^{S})\\ =\pi \displaystyle{\int}_{K \,{ > }\,k}{d}^{2}{{{\bf{K}}}}\,{q}_{e}\exp (-2{q}_{e}{z}_{0}){{{\rm{Im}}}}\left\{{b}_{e\,z}^{* }({{{\bf{K}}}}){{{{\bf{b}}}}}_{e\perp }({{{\bf{K}}}})\right.\\ \left.-{e}_{e\,z}^{* }({{{\bf{K}}}}){{{{\bf{e}}}}}_{e\perp }({{{\bf{K}}}})\right\}\end{array}$$

Where

$${{{{\bf{b}}}}}_{\perp }({{{\bf{K}}}})=({b}_{x}({{{\bf{K}}}}),{b}_{y}({{{\bf{K}}}}),0),\,\,\,\,{{{{\bf{e}}}}}_{\perp }({{{\bf{K}}}})=({e}_{x}({{{\bf{K}}}}),{e}_{y}({{{\bf{K}}}}),0)$$

Equation (49) shows a contribution from only the evanescent part of the angular spectrum of the fields and, as such, this integral is a near-field quantity. Notice the characteristic exponential *z*-decay as *z* = *z*_0_ increases, and that the transversal components, **E**_⊥_ and **B**_⊥_, contribute to this IMST flux in such a way that the component of this flow along *OZ*, $\int\nolimits_{-\infty }^{\infty }{d}^{2}{{{\bf{R}}}}({{{{\bf{P}}}}}_{m}^{S}-{{{{\bf{P}}}}}_{e}^{S})\cdot \hat{{{{\bf{z}}}}}$, is zero, as should be, in the decay direction. Hence, like the imaginary Poynting momentum^12^, although in the near-field and intermediate regions the IMST contributes locally to the ILF, beyond it conveys no force.

## Reactive forces on an electric and magnetic dipole

### The imaginary Maxwell stress tensor and the reactive force on a magnetoelectric dipolar particle

The flow CMST on a dipole is obtained in Appendix D of Supp. Mat. Calculations are done in the near-field zone of the emitter. We remark, however, that unless one makes the integration surface ∂*V*, enclosing the dipole, to strictly shrink into its center point, the flux RMST that yields the RLF is better obtained in the far-zone as done in ref. ^38^, where it also acquires a physical significance as the “radiated” MST flow, independent of the distance to the dipole, like the radiated energy^12^.

The reactive part, the IMST, stems from the near-field zone of the dipolar fields on the integration surface ∂*V*, Eq. (21). Hence there is no contribution from the far-zone. As stated above, the integration of the CMST in the far-field region is real. We address dipolar particles in the wide sense ^41,43^, namely whose polarizabilities are defined by the first electric and magnetic Mie coefficients.

The flow IMST depends on the integration surface, like the reactive Poynting vector flux^12^. Introducing the result of Appendix D (see Supp. Mat.) into Eq. (21), the reactive force on a small dipolar magnetoelectric particle is

50 $$\begin{array}{r}{{{{\mathcal{F}}}}}_{k}^{I}={{{\rm{Im}}}}\{{\int}_{\partial {V}_{0}}{d}^{2}r\,{T}_{kj}^{(mix)}{n}_{j}\}+\omega {\int}_{V\to {V}_{0}}{d}^{3}r\,[{{{{\bf{P}}}}}_{m}^{O}-{{{{\bf{P}}}}}_{e}^{O}]_k.\\ (j,k=1,2,3)\end{array}$$

where as seen in Appendix D:

51 $$\begin{array}{ll}{{{\rm{Im}}}}\{{\int}_{\partial {V}_{0}}{d}^{2}r\,{T}_{kj}^{(mix)}{n}_{j}\}&={{{\rm{Im}}}}\left\{\left[\frac{1}{10}(1-{{{\rm{i}}}}ka)\right.\right.\\&\quad \left.-\,\frac{1}{30}{k}^{2}{a}^{2}\right]\exp ({{{\rm{i}}}}ka)\,{p}_{j}\,[{\partial }_{k}{E}_{j}^{* }+{\partial }_{j}{E}_{k}^{* }]\\&\quad +\,\frac{1}{3}(1+{{{\rm{i}}}}ka)\exp (-{{{\rm{i}}}}ka)\,{p}_{j}^{* }{\partial }_{j}{E}_{k}\\& \quad\left.+\,\frac{1}{6}({k}^{2}{a}^{2}-{{{\rm{i}}}}ka)\exp (-{{{\rm{i}}}}ka){p}_{j}^{* }({\partial }_{k}{E}_{j}-{\partial }_{j}{E}_{k})\right\}\\ &\quad-\,{{{\rm{Im}}}}\left\{[\frac{1}{10}(1-{{{\rm{i}}}}ka)-\frac{1}{30}{k}^{2}{a}^{2}]\exp ({{{\rm{i}}}}ka){m}_{j}^{* }\,\left[{\partial }_{k}{B}_{j}\right.\right.\\ &\quad\left.+\,{\partial }_{j}{B}_{k}\right]+\frac{1}{3}(1+{{{\rm{i}}}}ka)\exp (-{{{\rm{i}}}}ka)\,{m}_{j}{\partial }_{j}{B}_{k}^{* }\\& \quad\left.+\,\frac{1}{6}({k}^{2}{a}^{2}-{{{\rm{i}}}}ka)\exp (-{{{\rm{i}}}}ka){m}_{j}({\partial }_{k}{B}_{j}^{* }-{\partial }_{j}{B}_{k}^{* })\right\}\end{array}$$

The superscript (*i*) has been omitted in (51), understanding that **E**, **B** represent the incident field. Equation (51) is the flow IMST across the minimum sphere ∂*V*_0_, of radius *a*, that contains the dipole. If this is a magnetoelectric spherical particle, ∂*V*_0_ is the particle surface taken from outside. In contrast with the RMST and time-averaged force, RLF, (see Appendix D), the flow IMST gives no interference between the induced electric and magnetic dipolar moments **p** and **m**. The superscript (*mix*) in Eq. (50) expresses quantities due to the interference of the incident and scattered fields, (cf. Appendiz D of Supp. Mat.).

As the exterior volume *V* tends to *V*_0_, the integral of (50) is :

52 $$\begin{array}{l}\displaystyle{\int}_{{V}_{0}}{d}^{3}r\,[{{{{\bf{P}}}}}_{m}^{O}-{{{{\bf{P}}}}}_{e}^{O}]={\int}_{\partial {V}_{0}}{d}^{2}r\,[{{{{\bf{P}}}}}_{m}^{O\,(mix)}-{{{{\bf{P}}}}}_{e}^{O\,(mix)}\,\,\,\,] \\+\displaystyle{\int}_{{V}_{0}}{d}^{3}r\,[{{{{\bf{P}}}}}_{m}^{O\,(in)}-{{{{\bf{P}}}}}_{e}^{O\,(in)}\,\,\,]\end{array}$$

The integral on ∂*V*_0_ is due to the contribution of the external ROM in *V* − *V*_0_ that as *V* → *V*_0_ shrinks into ∂*V*_0_. The superscript (*in*) denotes that the canonical momenta in (52) are those of the field inside the sphere. The interference ROM density is: ${({{{{\bf{P}}}}}_{e}^{O(mix)})}_{i}-{({{{{\bf{P}}}}}_{m}^{O(mix)})}_{i}=\frac{1}{8\pi \omega }{{{\rm{Im}}}}[{E}_{j}^{(i)\,* }{\partial }_{i}{E}_{j}^{(s)}+{E}_{j}^{(s)\,* }{\partial }_{i}{E}_{j}^{(i)}-{B}_{j}^{(i)\,* }{\partial }_{i}{B}_{j}^{(s)}-{B}_{j}^{(s)\,* }{\partial }_{i}{B}_{j}^{(i)}]$. In the next subsection [cf. Eq. (60)] we prove that for the scattered field one has that the quantity $\omega {\int}_{{V}_{0}}{d}^{3}r\,[{({{{{\bf{P}}}}}_{e}^{O(s)})}_{i}-{({{{{\bf{P}}}}}_{m}^{O(s)})}_{i}]=\frac{1}{8\pi }{\int}_{{V}_{0}}{d}^{3}r\,{{{\rm{Im}}}}[{E}_{j}^{(s)\,* }{\partial }_{i}{E}_{j}^{(s)}-{B}_{j}^{(s)\,* }{\partial }_{i}{B}_{j}^{(s)}]$, which would also appear in the right side of (52), is zero.

We next consider the simplified approximation of Eq. (51), (cf. Appendix D of Supp. Mat.):

53 $$\begin{array}{l}{{{\rm{Im}}}}\{{\int}_{\partial {V}_{0}}{d}^{2}r\,{T}_{kj}^{(mix)}{n}_{j}\}=-\frac{1}{10}{{{\rm{Im}}}}\left\{\left[{p}_{j}\,{\partial }_{k}{E}_{j}^{* }+{p}_{j}\,{\partial }_{j}{E}_{k}^{* }\right.\right.\\ \left.\left.\qquad\qquad\qquad\qquad\qquad\,\,\,-{m}_{j}\,{\partial }_{k}{B}_{j}^{* }-{m}_{j}\,{\partial }_{j}{B}_{k}^{* }\right]\right\},\,\,\,\,\,\,\,\,\,(j,k=1,2,3)\end{array}$$

[If no contribution of the radiative part of the scattered field **E**^(*s*)^ were considered in the interference terms, one would have an expression like (53) but with a factor 1/15, rather than 1/10, (see Appendix D)].

Let the electric and magnetic plarizabilities of the object be *α*_*e*_ and *α*_*m*_, so that **p** = *α*_*e*_**E**^(*i*)^, **m** = *α*_*e*_**B**^(*i*)^, (body chirality is outside our aim here), and assume $(1-{{{\rm{i}}}}ka)\exp ({{{\rm{i}}}}ka)\simeq 1$. Equation (53) may then be written as

54 $$\begin{array}{ll}{{{\rm{Im}}}}\{{\int}_{\partial {V}_{0}}{d}^{2}r\,{T}_{kj}^{(mix)}{n}_{j}\}&=-\frac{1}{20}\{{\alpha }_{e}^{I}\,\nabla | {{{\bf{E}}}}{| }^{2}-{\alpha }_{m}^{I}\,\nabla | {{{\bf{B}}}}{| }^{2}\}\\&\quad\, +\,\frac{4\pi \omega }{5}\{{\alpha }_{e}^{R}\,{{{{\bf{P}}}}}_{e}^{O}-{\alpha }_{m}^{R}\,{{{{\bf{P}}}}}_{m}^{O}\}\\&\quad\,-\frac{1}{10}{{{\rm{Im}}}}\{{\alpha }_{e}({{{\bf{E}}}}\cdot \nabla ){{{{\bf{E}}}}}^{* }-{\alpha }_{m}({{{\bf{B}}}}\cdot \nabla ){{{{\bf{B}}}}}^{* }\}\end{array}$$

The two terms of the third curly bracket of the right side in (54) come from the effect of the near field on the particle; as such, they have a resemblance with those of the time-averaged force on a quasistatic electric or magnetic dipole: $(1/2){{{\rm{Re}}}}\{{\alpha }_{e}({{{\bf{E}}}}\cdot \nabla ){{{{\bf{E}}}}}^{* }\}$ and $(1/2){{{\rm{Re}}}}\{{\alpha }_{m}({{{\bf{B}}}}\cdot \nabla ){{{{\bf{B}}}}}^{* }\}$^47^.

We note that the first and second curly brackets in (54), i.e., the gradient and canonical momentum terms, appear with the real and imaginary parts of the polarizabilities interchanged with respect to those of the time-averaged electric and magnetic forces (cf. Appendix D; see also ref. ^38^). Except when particles are strongly resonant, the imaginary parts of the polarizabilities are a factor *k*^3^*a*^3^ smaller than the real parts^38^. Thus the conservative gradient part of the reactive force would appear with a much smaller weight than its non-conservative component. By contrast, as we shall illustrate, in resonance such gradient reactive forces would be the strongest if the particle is not highly absorbing.

Introducing the Belinfante spin momentum and using the vector identities for divergenceless fields: ${{{\rm{Re}}}}[({{{\bf{E}}}}\cdot \nabla ){{{{\bf{E}}}}}^{* }]=\frac{1}{2}\nabla | {{{\bf{E}}}}{| }^{2}-k\,{{{\rm{Im}}}}[{{{\bf{E}}}}\times {{{{\bf{B}}}}}^{* }]$; ${{{\rm{Im}}}}[({{{\bf{E}}}}\cdot \nabla ){{{{\bf{E}}}}}^{* }]=\frac{1}{2}\nabla \times {{{\rm{Im}}}}({{{{\bf{E}}}}}^{* }\times {{{\bf{E}}}})$; with analogous relations for the **B**-vector, then the simplified approximation to the IMST, Eq. (54), may be expressed in terms of the conservative gradient components, the real and imaginary field (Poynting) momenta and the canonical momenta, as

55 $$\begin{array}{ll}{{{\rm{Im}}}}\{{\int}_{\partial {V}_{0}}{d}^{2}r\,{T}_{kj}^{(mix)}{n}_{j}\}&=-\frac{1}{10}\{{\alpha }_{e}^{I}\nabla | {{{\bf{E}}}}{| }^{2}-{\alpha }_{m}^{I}\nabla | {{{\bf{B}}}}{| }^{2}\}\\&\quad\, +\,\frac{4\pi \omega }{5}\left\{({\alpha }_{m}^{R}-{\alpha }_{e}^{R}) \,< {{{\bf{g}}}} >\, +({\alpha }_{e}^{I}-{\alpha }_{m}^{I}){{{{\bf{g}}}}}^{I}\right.\\ &\quad\,\left.+\,2({\alpha }_{e}^{R}{{{{\bf{P}}}}}_{e}^{O}-{\alpha }_{m}^{R}{{{{\bf{P}}}}}_{m}^{O})\right\}\end{array}$$

The sign of the gradient component of the IMST depends on that of the first curly bracket. In the next examples we shall show that the accretion of ROM in and around the particle, influences the “radiated” (i.e., time-averaged) RMST into the far-zone, and hence the RLF.

In Appendix E of Supp. Mat. we heuristically intend, as an addenda to the above equations, to derive the ILF on an electric and a magnetic dipole following the procedure employed in ref. ^5^ for the RLF. It is intriguing that, for example on a purely electric dipolar particle hit by a linearly polarized plane wave, that ILF is five times the expression given by Eq. (53) or its equivalent (55). In the Example 2, below, we shall analyze this latter case, showing the correctness of the ILF obtained in Appendix E.

However, the ILF on a purely magnetic dipolar particle obtained in Appendix E presents a sign opposite to that of Eq. (53). In fact, the polarization current density holds: $\nabla \cdot \vec{{{\mathcal{J}}}}=\nabla \cdot d \vec{{{\mathcal{P}}}}/dt$ (see e.g.,^48^, Section 14.2.2), and thus one may add to the relationship: $\vec{{{\mathcal{J}}}}=d\vec{{{\mathcal{P}}}}/dt$ any term $\nabla \times \vec{{{\mathcal{M}}}}$. Hence, given the lack of experimental evidence yet, we have an indeterminacy of such a term. Further research may clarify whether this ambiguity causes the failure of the equations of Appendix E to describe the reactive force on resonant dipolar particles, (see Examples 4 and 5 below); while the term $\nabla \times \vec{{{\mathcal{M}}}}$, if relevant, appears to be zero for non-resonant dipoles, as seen in Example 2. Hence, taking into account that the complex Maxwell stress tensor theorem and its consequences is an unexplored territory, the formulation of Appendix E on dipolar particles deserves further experimental and theoretical research.

### Further physical consequences. The overall spin momentum. The interior and external reactive strength of orbital momentum

As stated in Appendix D of the Sup. Mat., the far-zone flow IMST on a dipole, is zero term by term since only the diagonal elements of the CMST, which are real, contribute to it, yielding the time-averaged force, (see also ref. ^38^). So we infer that, in addition to (31), we have

56 $${\int}_{{V}_{\infty }}{d}^{3}r\,{{{{\bf{P}}}}}_{m}^{S}={\int}_{{V}_{\infty }}{d}^{3}r\,{{{{\bf{P}}}}}_{e}^{S}=0$$

Equation (56) states *the vanishing of the overall electric and magnetic spin momenta of the total field, (i.e., incident plus scattered) from a dipole*. It includes the object volume *V*_0_ of charges and currents. This is a generalization of a previous result^28^ for the spin momentum of a free field or of an evanescent wave^28^. As seen from Appendix D, Eq. (31), but not Eq. (56), also holds for the scattered field.

Since, as we have seen in connection with Eq. (25), as *V* = *V*_*∞*_ one has that ${{{{\bf{P}}}}}_{m}^{O\,FF}={{{{\bf{P}}}}}_{e}^{O\,FF}$, it is likely that (56) holds for the total field from any arbitrary body. However, we have not proven it here. Note, nonetheless, that although the spin momentum of the total field is zero in the whole space with sources, this momentum is not a virtual quantity locally. Indeed the Belinfante spin momentum gives rise to forces near surfaces, like it happens with the transversal force, which depends on the reactive helicity of evanescent waves created on ∂*V*_0_^12,28^.

The fields **E**^(*in*)^, **B**^(*in*)^ inside the object volume *V*_0_ cannot produce any net force on the body. Therefore using Eq. (17) for these fields one has: ${\int}_{\partial {V}_{0}}{d}^{2}r\,{T}_{ij}^{(in)}{n}_{j}=-{{{\rm{i}}}}\omega {\int}_{{V}_{0}}{d}^{3}r\,[{{{{\bf{P}}}}}_{m}^{O\,(in)}-{{{{\bf{P}}}}}_{e}^{O\,(in)}]$.

On the other hand, we may write an equation like (21) for the scattered field in an arbitrary volume *V* enclosing the dipole

57 $${{{\rm{Im}}}}\{{{{{\mathcal{F}}}}}_{i}^{(s)}\}= {\int}_{\partial V}{d}^{2}r\,{{{\rm{Im}}}}\{{T}_{ij}^{(s)}\}{n}_{j}+{{{\rm{i}}}}\omega {\int}_{V}{d}^{3}r\,{[{{{{\bf{P}}}}}_{m}^{O(s)}-{{{{\bf{P}}}}}_{e}^{O(s)}]}_{i}$$

where ${{{\rm{Im}}}}\{{{{{\mathcal{F}}}}}_{i}^{(s)}\}$ is a force on the particle due to the scattered field only, certainly different from the actual reactive force ${{{{\mathcal{F}}}}}_{i}^{I}$ stemmed from the total field.

Taking into account that, as seen in Eq. (57), if *V* = *V*_*∞*_ one has ${\int}_{\partial {V}_{\infty }}{d}^{2}r\,{{{\rm{Im}}}}\{{T}_{ij}^{(s)}\}{n}_{j}=0$ since ${\int}_{\partial {V}_{\infty }}{d}^{2}r\,{T}_{ij}^{(s)}{n}_{j}$ is real, (see also^38^), the above equation (57) becomes

58 $${{{\rm{Im}}}}\{{{{{\mathcal{F}}}}}_{i}^{(s)}\}={{{\rm{i}}}}\omega {\int}_{\!{V}_{\infty }}{d}^{3}r\,[{{{{\bf{P}}}}}_{m}^{O\,(s)}-{{{{\bf{P}}}}}_{e}^{O\,(s)}]_i$$

which is similar to (24). Then introducing (58) into (57) we obtain in terms of the ROM of the scattered field

59 $${\int}_{\!\partial V}{d}^{2}r\,{{{\rm{Im}}}}\{{T}_{ij}^{(s)}\}{n}_{j}={{{\rm{i}}}}\omega {\int}_{\!{V}_{\infty }-V}{d}^{3}r\,{[{{{{\bf{P}}}}}_{m}^{O(s)}-{{{{\bf{P}}}}}_{e}^{O(s)}]}_{i}$$

which for the scattered field is analogous to (25). Now, according to Appendix D, on a dipolar particle: ${\int}_{\partial {V}_{0}}{d}^{2}r\,{{{\rm{Im}}}}\{{T}_{ij}^{(s)}\}{n}_{j}=0$, where *V*_0_ is the smallest sphere enclosing the dipole, which for such a particle we take as its volume. Then (59) reduces to

60 $${\int}_{\!{V}_{\infty }-{V}_{0}}{d}^{3}r\,[{{{{\bf{P}}}}}_{m}^{O\,(s)}-{{{{\bf{P}}}}}_{e}^{O\,(s)}]=0$$

Therefore we conclude that the ROM of the field scattered from a dipolar particle is zero, independently of whether it is a Rayleigh one or dipolar in the wide sense^38,39^. Hence, *the ILF on a dipolar particle, Eq*. (50)*, comes exclusively from those terms of both the IMST and ROM that contain interference of the incident and scattered fields*. We think that this likely occurs with an arbitrary body, although we have not proven it here.

### Example 2: Linearly polarized plane wave impinging on a low refractive index small dielectric particle

Consider a low refractive index dielectric particle, like a polystyrene sphere, (refractive index *n*_*PS*_ = 1.59, radius *a* = 50 nm), illuminated by a linearly polarized propagating plane wave:

61 $${{{\bf{E}}}}={E}_{0}(1,0,0){e}^{{{{\rm{i}}}}kz},\,\,\,\,\,\,\,\,{{{\bf{B}}}}={E}_{0}(0,1,0){e}^{{{{\rm{i}}}}kz}$$

*E*_0_ being a constant. The incident momenta are:

62 $${{{{\bf{P}}}}}_{e}^{O}={{{{\bf{P}}}}}_{m}^{O}= < {{{\bf{g}}}} > =\frac{1}{8\pi c}{E}_{0}^{2}\,\hat{{{{\boldsymbol{z}}}}}$$

For the time-averaged and the reactive forces we test Eqs. (E3) and (E4) of Appendix E in the Supp. Mat., which yield

63 $$< \vec{{{{\bf\mathcal{F}}}}}_{e} > =\frac{1}{2}{\alpha }_{e}^{I}\,k\,{E}_{0}^{2}\,\hat{{{{\boldsymbol{z}}}}},\,\,\,\,\,\,\,\,\vec{{{{\bf\mathcal{F}}}}}_{e}^{I}=\frac{1}{2}{\alpha }_{e}^{R}\,k\,{E}_{0}^{2}\,\hat{{{{\boldsymbol{z}}}}}$$

Note that, as mentioned above, under plane wave illumination on a purely electric dipolar particle, the ILF $\vec{{{{\bf\mathcal{F}}}}}_{e}^{I}$, Eq. (63), is five times its IMST component given by the approximate equation (55). The numerical computation methods in this and following examples is the same, and indicated in Appendix G of the Supp. Mat..

**Fig. 2:**
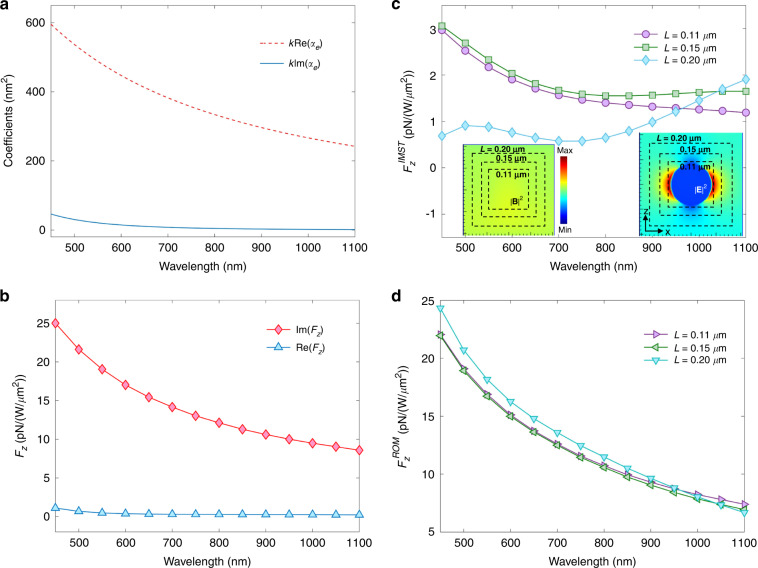
Linearly polarized propagating plane wave incident on a polystyrene sphere of radius *a* = 50 nm and refractive index *n*_*PS*_ = 1.59. **a** Polarizability calculated via Mie theory. **b** Numerical results, per unit of incident power density, *E*_0_ = 1, for the ILF ${F}_{z}^{I}$, and < *F*_*z*_>, on the sphere. **c** IMST component, ${F}_{z}^{IMST}$, of ${F}_{z}^{I}$, calculated with different cube integration contours. Insets show the spatial distribution on the *x* = 0 plane of the electric and magnetic field intensities in and around the sphere excited at *λ* = 800 nm. White broken line squares illustrate the integration contours. **d** ROM component, ${F}_{z}^{ROM}$, of the ILF, calculated by subtracting ${F}_{z}^{IMST}$ from ${F}_{z}^{I}$

The real and imaginary parts of the electric polarizability, ${\alpha }_{e}^{R}$ and ${\alpha }_{e}^{I}$, as well as $<\vec {{{{\bf\mathcal{F}}}}}_{e} >$ and $\vec{{{{\bf\mathcal{F}}}}}_{e}^{I}$, are plotted versus *λ* in the visible and near-infrared in Fig. 2(a) and (b). The ILF, $\vec{{{{\bf\mathcal{F}}}}}_{e}^{I}$, of (63) agrees well with its numerical calculation through the charge and current volume integral of the left side of Eq. (21) and following the procedure described in Appendix G^49^. For example for *λ* = 450 nm Fig. 2(a) reads $k{\alpha }_{e}^{R}=600\times 1{0}^{-18}\,{{{\rm{{m}}}^{2}}}$ per unit of incident power density. This yields an ILF per unit power: 25 pN/(W/*μ*m^2^), which coincides with its value in Fig. 2(b). In the same way, there is agreement between the RLF of (63) according to Fig. 2(a) and its numerical computation of Fig. 2(b), as it should.

As seen, the ILF is totally dominant upon the time-averaged force, specially at higher frequencies which lie in the visible region. This illustrates the physics described in this work; namely, underneath a weaker time-averaged force, there is a relatively large build-up of ROM and thus of reactive force. This is shown in Fig. 2(c) of the IMST component, ${F}_{z}^{IMST}$, numerically obtained from the IMST, Eq. (22), evaluated from the total field through an FDTD computation via the Mie series, and Fig. 2(d) of the ROM component, ${F}_{z}^{ROM}$, obtained by substracting ${F}_{z}^{IMST}$ from ${F}_{z}^{I}$. We observe that ${F}_{z}^{IMST}\ll {F}_{z}^{ROM}$, so that the ILF is largely due to its stronger ROM component.

The aforementioned counteraction of both the ILF and ROM on the RLF, evident in these figures, indicates the observability of the ILF and ROM, as well as the reactive nature of this near-field radiation pressure versus the “far-field” flow RMST that yields the time-averaged force. Thus, underlying the weak RLF, $< \vec{{{{\bf\mathcal{F}}}}}_{e} >$, exerted by an incident wave on a low refractive index dielectric particle, there are a dominant ROM and reactive force. This has an analogy with the detrimental effect of the reactive work, associated to the reactive power, over the time-averaged work and power radiated by an emitter into the far-zone^12,16,45^.

Although the incident optical angular momentum gives rise to a reactive optical torque, as shown in Appendix B of Supp. Mat., and twisted structured light may have an effect on the ILF, the angular momentum of a beam similar to a circularly polarized (CP) plane wave does not alter the reactive force, $\vec{{{{\bf\mathcal{F}}}}}^{I}$, of Eq. (63), which therefore also applies for CP incidence. The same as it happens with the time-averaged force. As a matter of fact, it is straightforward to see that the incident canonical momentum, Eq. (62), is insensitive to the spin in this case.

This is illustrated in Fig. S1 of Appendix F of the Supp. Mat., which compares the electric field spatial distribution and the reactive force from a linearly polarized (LP) plane wave with those pertaining to circular polarization (CP); both impinging on the above discussed PS particle. The angular momenta of more complex structured light fields may, however, have an effect on the ILF. This question is left open for future research.

### Example 3: Linearly polarized Gaussian beam incident on a small particle, electrically dipolar

We address a beam with Gaussian transversal profile, incident on an electrically dipolar dielectric particle

64 $${{{\bf{E}}}}={E}_{0}\,{\exp}{\left(-\frac{{R}^{2}}{{\sigma }^{2}}\right)}(1,0,0){\exp}({{{\rm{i}}}}kz),\,\,\,\,\,\,\,(R=\sqrt{{x}^{2}+{y}^{2}})$$

Now the gradient component of the RLF and ILF appear. We have

65 $${{{{\bf{P}}}}}_{e}^{O}={{{{\bf{P}}}}}_{m}^{O}=\frac{1}{8\pi c}{E}_{0}^{2}\,{\exp}{\left(-\frac{{R}^{2}}{{\sigma }^{2}}\right)}\,\hat{{{{\boldsymbol{z}}}}}$$

and

66 $$\nabla | {{{\bf{E}}}}{| }^{2}=-\frac{4}{{\sigma }^{2}}{E}_{0}^{2}\,{\exp}{\left(-\frac{{R}^{2}}{{\sigma }^{2}}\right)}(x,y,0)$$

So that

67 $$\begin{array}{*{20}{l}}{ < {{\vec {\cal F}}_e} > }&{ = E_0^2 \, \exp \left( - \frac{{{R^2}}}{{{\sigma ^2}}}\right)\left[ - \frac{{\alpha _e^R}}{{{\sigma ^2}}}(x,y,0) + \frac{{\alpha _e^I}}{2}\, k \, (0,0,1)\right],}\\{{\rm{Im}}\{ {{\vec {\cal F}}_e}\} }&{ = E_0^2 \, \exp \left( - \frac{{{R^2}}}{{{\sigma ^2}}}\right)\, \left[\frac{{4\alpha _e^I}}{{{\sigma ^2}}}(x,y,0) + \alpha _e^R\, k\, (0,0,1)\right]}\end{array}$$

which shows the Hooke behavior of the conservative gradient component as expected in optical tweezer set-ups, along with the pushing nature of the ILF gradient component, as well as of the RLF at wavelengths where ${\alpha }_{e}^{R}\, < \,0$, case in which the scattering ILF becomes pulling. Therefore, this example illustrates the exchange of ${\alpha }_{e}^{R}$ and ${\alpha }_{e}^{I}$ between the RLF and ILF in an optical manipulation set-up through their gradient and scattering components.

### Example 4: Linearly polarized plane wave incident on a magnetoelectric high refractive index dipolar particle

Magnetoelectric particles are of great importance in nanophotonics^50–58^. Given the well-known scaling property of high index spheres, results in a certain range of wavelengths are transposable to another spectrum in particles with a different refractive index. It is, therefore, relevant to study this case as regards the build up of ROM and reactive forces.

**Fig. 3:**
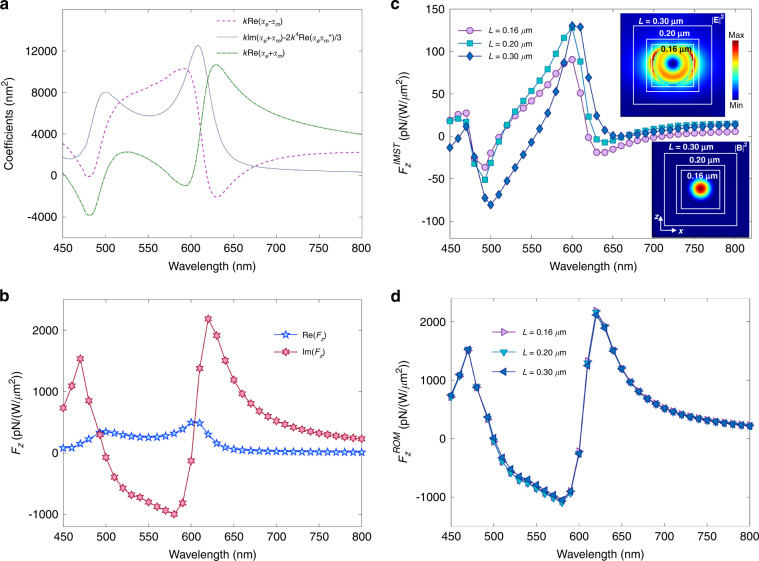
Linearly polarized propagating plane wave incident on a Si sphere of radius *a* = 75 nm. *E*_0_ = 1. **a** Combined polarizabilities calculated via Mie theory. The expression of < *F*_*z*_ > given by these polarizabilities according to ref. ^38^ is shown in the full line, while the broken one depicts the theoretical flow IMST, ${F}_{z}^{IMST}$, component of the reactive force, ${F}_{z}^{I}$, according to Eq. (55). **b** Numerical results for the time-averaged force < *F*_*z*_ > and reactive force ${F}_{z}^{I}$ on the sphere. **c** IMST component, ${F}_{z}^{IMST}$, of the ILF numerically calculated with different cube integration contours. Insets show maps on the *x* = 0 plane of ∣**E**∣^2^ and ∣**B**∣^2^ at *λ* = 610 nm which corresponds to the magnetic dipole resonance. White broken line squares illustrate the integration contours. **d** ROM component, ${F}_{z}^{ROM}$, of the ILF calculated by subtracting ${F}_{z}^{IMST}$ from ${F}_{z}^{I}$

We consider the plane wave of Example 2 incident on a Si sphere of radius 75 nm. Figure 3(a) depicts the RLF, the predicted IMST component, ${{{\rm{Im}}}}\{{\int}_{\partial {V}_{0}}{d}^{2}r\,{T}_{kj}^{(mix)}{n}_{j}\}=(4\pi \omega /5)({\alpha }_{e}^{R}-{\alpha }_{m}^{R})$, according to the approximation: (55), and the factor $k({\alpha }_{e}^{R}+{\alpha }_{m}^{R})$ that yields the ILF according to Eq. (E21) of the Supp. Mat.

The RLF, ${F}_{z}^{R}$, and ILF, ${F}_{z}^{I}$, numerically computed from the charge and current integral of the left side of Eqs. (20) and (21) as indicated in Appendix G, are shown in Figure 3(b). Now the effect of the electric and magnetic dipole resonances of the particle in the proximities of 500 and 610 nm, respectively, is observed. Comparing Fig. 3(a) and (b) we note that Eq. (E21) does not yield the correct ILF; neither Eq. (E20). In contrast with Example 2 of a non-resonant particle.

The shape of the computed spectrum of the flow IMST component ${F}_{z}^{IMST}$, shown in Fig. 3(c), is similar to the theoretical ILF (63) which follows the broken line of Fig. 3(a) according to Eq. (55). The IMST force most similar to that of (55) is that obtained on the cube contour ∂*V* with *L* = 0.16 μ*m*, almost tangent to the particle. On the other hand, a comparison of Fig. 3(b) and (d) shows an almost total contribution of the ROM to the ILF, manifesting the small weight of ${F}_{z}^{IMST}$ in the reactive force on this resonant particle. Figure 3(b) and (c) indicate that ${F}_{z}^{IMST}$ is of the same order of magnitude as the time-averaged force in the whole range of wavelengths, while the ILF, ${F}_{z}^{I}$, is much stronger than <*F*_*z*_>, and enhanced near the electric and magnetic resonances, although its sharp variation makes it to be near zero at the resonant wavelengths where the “radiated” force, <*F*_*z*_>, is maximum. This latter feature keeps an analogy with the reactive power and helicity^12,19^ which are near-zero at resonant wavelengths at which there is maximum radiated power. As a noted illustration, a photoinduced force microscopy experiment^59^ detects the resonant force signal, on excitation of the magnetic dipole of a Si resonator, associated with the enhancement of its quality factor and external stored reactive power.

Actually, we observe that ${F}_{z}^{I}$ is almost entirely due to the large ROM contribution, ${F}_{z}^{ROM}$, and its enhancements, as depicted in Fig. 3(d). This once again illustrates the detrimental effect of this dominant “reactance” ILF on the “dynamic radiative efficiency” constituted by the time-averaged radiation force RLF. Now, according to (24) the ILF equals the overall ROM, while we see from (25) that ${F}_{z}^{IMST}$ is given by the external ROM, (which as remarked above is due to interference of the incident and scattered fields).

Therefore in a high index magnetoelectric particle, most of the ROM is built inside it; the reactive ILF therewith mainly being due to this internal ROM and its contribution, ${F}_{z}^{ROM}$, is completely dominant upon the time-averaged force.

### Example 5: Linearly polarized plane propagating wave incident on a plasmonic particle

**Fig. 4:**
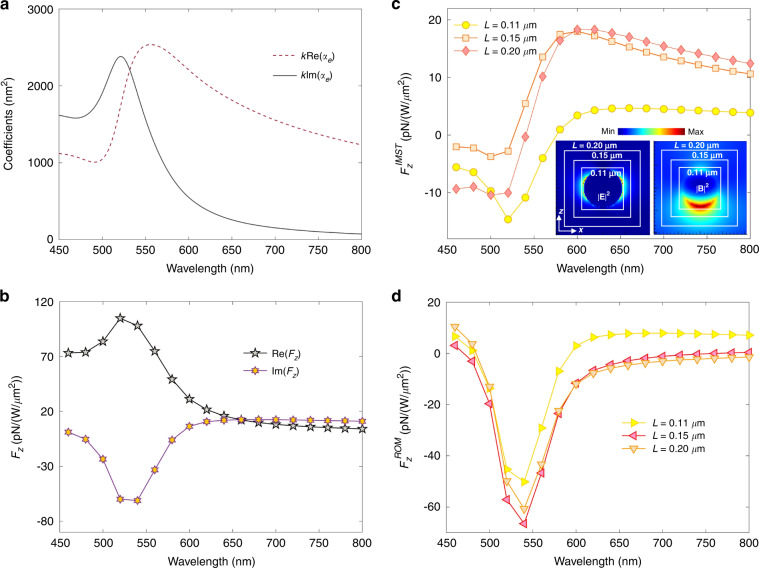
Linearly polarized propagating plane wave incident on an Au sphere of radius *a* = 50 nm. *E*_0_ = 1. **a** Polarizability calculated via Mie theory. **b** Numerical results for the time-averaged force < *F*_*z*_ > and reactive force ${F}_{z}^{I}$ on the sphere. *E*_0_ = 1. **c** IMST component ${F}_{z}^{IMST}$ of the ILF calculated with different cube integration contours. Insets show the spatial distribution on the *x* = 0 plane of the electric and magnetic field intensities at the wavelength of 540 nm. White broken line squares illustrate the integration contours. **d** ROM component, ${F}_{z}^{ROM}$, of the ILF calculated by subtracting ${F}_{z}^{IMST}$ from ${F}_{z}^{I}$

As seen in the previous examples, almost all contribution to the ILF is due to the internal ROM. Absorption in the volume distribution of charges and currents changes this; it may extract energy from the reactive power^44,60,61^. A decrease of reactive quantities would happen even in situations where both the RLF and ILF are resonant. To illustrate it, we now consider the above plane wave incident on an plasmonic Au spherical particle of radius *a* = 50 nm, whose polarizability is shown in Fig. 4(a). A computation, as described in Appendix G of Supp. Mat., yields the time-averaged force, <*F*_*z*_> and reactive force, ${F}_{z}^{I}$, on this particle, [cf. Fig. 4(b)]. On the other hand, Fig. 4(c) and (d) depict ${F}_{z}^{IMST}$ and ${F}_{z}^{ROM}$, respectively.

We see that while <*F*_*z*_> follows the theoretical expression (63) obtained from Appendix E, ${F}_{z}^{I}$ does not, specially in the region of the plasmon resonance peak where in spite of the pulling ILF enhancement, the RLF is stronger. Moreover, at *λ* < 450 nm the RLF is totally dominant versus a very small ILF, while the opposite occurs at *λ* > 650 nm, in accordance with the expected antagonistic role of both kind of forces. Also, as the internal electric field is practically zero, although the inner **B** does not vanish, the weight of the internal ROM, i.e., of ${F}_{z}^{ROM}$ [cf. Fig. 4(d)] is now much smaller than in the non-lossy particles of the previous examples. The contribution of ${F}_{z}^{IMST}$ [cf. Fig. 4(c)] to the ILF is now non-negligible versus that of the internal ROM plus the ROM in the four corners between the integration cube and the particle surface. So we see that field losses in Au are responsible of diminishing the internal ROM contribution to the ILF versus that of the IMST.

**Fig. 5:**
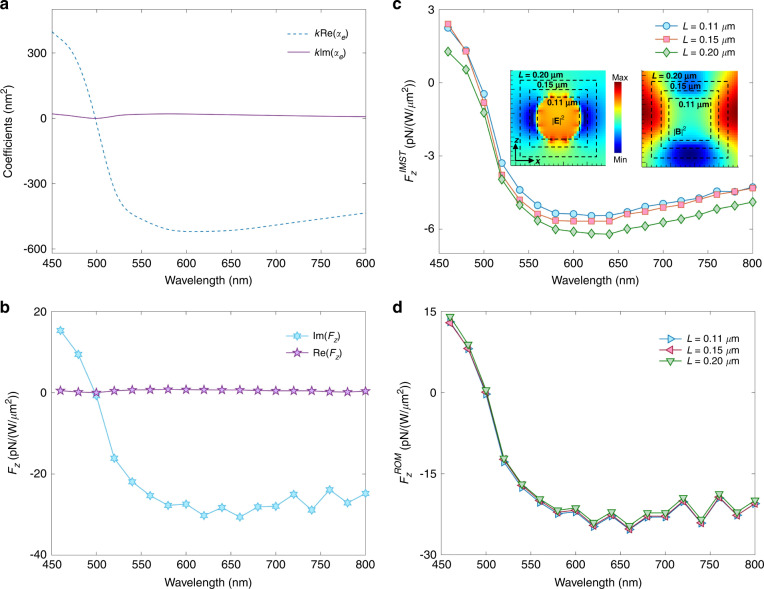
Same as Fig. 4 for a sphere of a hypothetical material, that we call Au_3_, whose refractive index has a real part identical to that of Au, but its imaginary part is artificially set to zero. **a** Calculated polarizibility. **b** Real and imaginary parts of complex Lorentz force. **c** IMST and **d** ROM components of of the ILF

To get a closer look to the effect of absorption, Fig. 5(a)–(d) depict the same as before for a hypothetical particle (Au_3_) whose refractive index ${\hat{n}}_{Au3}$ has a real part *n*_*Au*3_ identical to that of Au, *n*_*Au*_, but whose imaginary part *κ*_*Au*3_ has artificially been set to zero. We see that now, as no resonance is present, all quantities behave as in a dielectric sphere, [compare with Fig. 2(a)–(d)]. The ILF follows the heuristic theoretical equation (63) and the antagonic role of both forces is now retrieved. It is interesting, nevertheless, that close to the wavelength where the ILF changes sign, both forces coincide being practically zero.

**Fig. 6:**
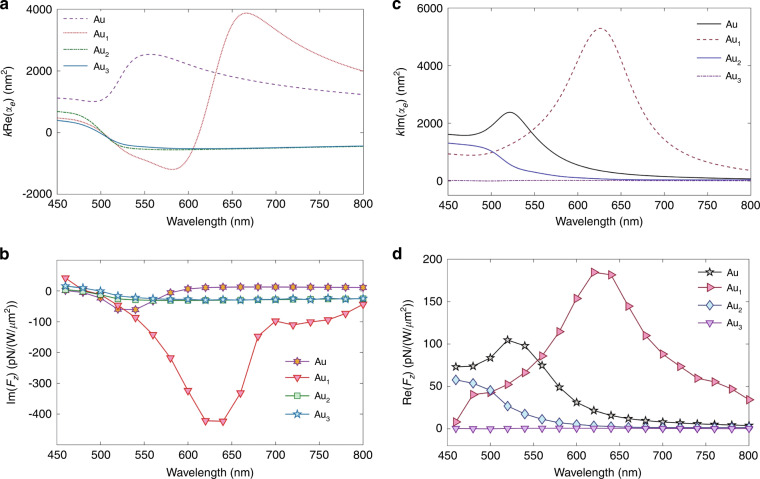
Linearly polarized propagating plane wave incident on a sphere with radius a = 50 nm of gold and of three hypothetical materials. **a** Real part of polarizability of a sphere of radius *a* = 50 nm, from Mie theory of: Au and three hypothetical materials denoted as Au_1_, Au_2_ and Au_3_, whose refractive index real parts are equal to those of Au, but their imaginary parts are artificially set to: A half of Au imaginary part (Au_1_), the same as the real part (Au_2_), and zero (Au_3_). **b** Numerical results of ILF, ${F}_{z}^{I}$, on each of these spheres illuminated by a linearly polarized plane wave with amplitude *E*_0_ = 1. **c** Imaginary part of polarizability of the four spheres. **d** Numerical calculation of RLF, <  *F*_*z*_>, on each of these spheres under the same incident wave as in **b**

In this connection, Fig. 6(a)–(d) show a direct comparison of the polarizability, RLF, and ILF for the Au sphere and three hypothetical particles whose refractive index real part is the same as that of Au, ${\hat{n}}_{Au}$ but with the imaginary part artificially chosen as: a half that of Au (Au_1_), the same as the real part (Au_2_), and zero (Au_3_). In the Au_1_ case the plasmon resonance is much stronger than in Au, but although the RLF is accordingly stronger, the pulling ILF more than doubles the RLF. The antagonistic role between both forces is observed in the Au_2_ and Au_3_ cases.

## Recapitulation: Discussion on the complex stress tensor theorem in a dielectric medium

If the medium surrounding the illuminated body is not vacuum, but has a non-unity refractive index *n*, there is an ongoing debate on the form of the field momentum. Thus one may ask how this will affect the above formulation of the CMST theorem and the ILF.

Among all proposals for the Poynting momentum and Maxwell stress tensor, we make here a discussion of this question by focusing on the Abraham and Minkowski forms, which are those whose respective ranges of validity have been more countersigned by experiments^8,62^. Further study on other formulations is outside the aims of this paper.

The Minkowski, $\vec{{\bf\mathfrak{G}}}^{M}$, and Abraham, $\vec{{\bf\mathfrak{G}}}^{A}$, field momentum densities of an electromagnetic wave in an isotropic, linear, homogeneous nonabsorbing and nondispersive medium of time-independent refractive index $n=\sqrt{\epsilon \mu }\,\ne\, 1$ are, in terms of the real electric and magnetic vectors, $\vec{\bf\mathfrak{E}}$ and $\vec{\bf\mathfrak{H}}=(1/\mu )\vec{\bf\mathfrak{B}}$, in Gaussian units employed in this work^62^:

68 $$\vec{{\bf\mathfrak{G}}}^{M}=\frac{\epsilon \mu }{4\pi c}\vec{\bf\mathfrak{E}}({{{\bf{r}}}},t)\times \vec{\bf\mathfrak{H}}({{{\bf{r}}}},t)=\epsilon \mu \,\vec{{\bf\mathfrak{G}}}^{A}$$

For the fields represented by the analytic signals we thus introduce the Minkowski and Abraham complex Poynting momentum densities:

69 $$\vec{{{{\bf\mathcal{G}}}}}^{M}=\frac{\epsilon \mu }{8\pi c}\vec{{{\bf\mathcal{E}}}}({{{\bf{r}}}},t)\times {{\vec{\bf\mathcal{{H}}}^{* }}}({{{\bf{r}}}},t)=\epsilon \mu \,\vec{{{{\bf\mathcal{G}}}}}^{A}$$

whose real and imaginary parts define the time-averaged and imaginary Minkowski and Abraham Poynting momentum densities, respectively.

Then the complex Maxwell stress tensor theorem reads according to Minkowski:

70 $$\begin{array}{l}{{{{\mathcal{F}}}}}_{i}=-\displaystyle{\int}_{V}{d}^{3}r\,{\partial }_{t}{{{{\mathcal{G}}}}}_{i}^{M\,* }+{\int}_{\partial V}{d}^{2}r\,{{{{\mathcal{T}}}}}_{ij}^{M}{n}_{j}\\ \quad\quad\,\,+\,{{{\rm{i}}}}\omega \displaystyle{\int}_{V}{d}^{3}r{[\vec{{{{\mathcal{P}}}}}_{m}^{OM}-\vec{{{{\mathcal{P}}}}}_{e}^{OM}]}_{i}\end{array}$$

With the Minkowski complex stress tensor:

71 $${{{{\mathcal{T}}}}}_{ij}^{M}=\frac{1}{8\pi }\left[\epsilon {{{{\mathcal{E}}}}}_{i}{{{{\mathcal{E}}}}}_{j}^{* }+\frac{1}{\mu }{{{{\mathcal{B}}}}}_{i}^{* }{{{{\mathcal{B}}}}}_{j}-\frac{1}{2}{\delta }_{ij}(\epsilon | {{{\mathcal{E}}}}{| }^{2}+\frac{1}{\mu }| {{{\mathcal{B}}}}{| }^{2})\right]$$

and Minkowski orbital momenta:

72 $$\begin{array}{r}{({{{{\mathcal{P}}}}}_{e}^{OM})}_{i}=\frac{\epsilon }{8\pi \omega }{{{\rm{Im}}}}[{{{{\mathcal{E}}}}}_{j}^{* }{\partial }_{i}{{{{\mathcal{E}}}}}_{j}],\\ {({{{{\mathcal{P}}}}}_{m}^{OM})}_{i}=\frac{1}{8\pi \mu \omega }{{{\rm{Im}}}}[{{{{\mathcal{B}}}}}_{j}^{* }{\partial }_{i}{{{{\mathcal{B}}}}}_{j}]\end{array}$$

Whereas according to Abraham it should be:

73 $$\begin{array}{l}{{{{\mathcal{F}}}}}_{i}+{{{{\mathcal{F}}}}}_{i}^{A}=-\displaystyle{\int}_{V}{d}^{3}r\,{\partial }_{t}{{{{\mathcal{G}}}}}_{i}^{A\,* }+\displaystyle{\int}_{\partial V}{d}^{2}r\,{{{{\mathcal{T}}}}}_{ij}^{M}{n}_{j}\\\qquad\qquad\quad +\,{{{\rm{i}}}}\omega \displaystyle{\int}_{V}{d}^{3}r{[{{{{\mathcal{P}}}}}_{m}^{OM}-{{{{\mathcal{P}}}}}_{e}^{OM}]}_{i}\end{array}$$

where ${{{{\mathcal{F}}}}}_{i}^{A}=(\epsilon \mu -1){\int}_{V}{d}^{3}r\,{\partial }_{t}{{{{\mathcal{G}}}}}_{i}^{A\,* }$, and we recall that in Eqs. (70) and (73) ${{{{\mathcal{F}}}}}_{i}=\frac{1}{2}{\int}_{V}{d}^{3}r\,({\rho }^{* }\vec{{{\bf\mathcal{E}}}}+\frac{1}{c}\vec{{{{\bf\mathcal{J}}}}}^{* }\times \vec{{{\bf\mathcal{B}}}})_i$.

Although on using (69) in (73) one inmediately retrieves (70), Eq. (73) exhibits a first term on the right side that, in analogy with the first term in the right-hand side of (70), suggests that $\vec{{{{\mathcal{G}}}}}^{A\,* }$ should be the complex momentum density of the field, producing a complex Abraham force on the object given by the sum of the complex Lorentz force $\vec{{{{\mathcal{F}}}}}$ and $\vec{{{{\mathcal{F}}}}}^{A}$.

In consequence, the reactive force produced by a general time-dependent field on a body in a medium is given by the imaginary part of either Eq. (70) or (73), depending on the choice of Poynting momentum. The reactive force that the Abraham momentum predicts does not coincide with the ILF, but contains the additional component ${{{\rm{Im}}}}\{\vec{{{{\mathcal{F}}}}}^{A}\}$.

For time-harmonic fields, both $\vec{{{{\mathcal{G}}}}}^{M\,* }$ and $\vec{{{{\mathcal{G}}}}}^{A\,* }$ become time-independent, and therefore (70) and (73) yield the same CMST equation, identical to (17) with the Minkowski CMST pertaining to the embedding medium:

74 $${T}_{ij}^{M}=\frac{1}{8\pi }\left[\epsilon {E}_{i}{E}_{j}^{* }+\frac{1}{\mu }{B}_{i}^{* }{B}_{j}-\frac{1}{2}{\delta }_{ij}(\epsilon | {{{\bf{E}}}}{| }^{2}+\frac{1}{\mu }| {{{\bf{B}}}}{| }^{2})\right]$$

and the orbital momentum densities:

75 $$\begin{array}{r}{({{{{\boldsymbol{P}}}}}_{e}^{OM})}_{i}=\frac{\epsilon }{8\pi \omega }{{{\rm{Im}}}}[{E}_{j}^{* }{\partial }_{i}{E}_{j}],\\ {({{{{\boldsymbol{P}}}}}_{m}^{OM})}_{i}=\frac{1}{8\pi \mu \omega }{{{\rm{Im}}}}[{B}_{j}^{* }{\partial }_{i}{{{{\mathcal{B}}}}}_{j}]\end{array}$$

Then the reactive force and the ILF coincide, being given by an equation identical to (21) with the Minkowski IMST: ${T}_{ij}^{M\,I}=\frac{1}{8\pi }{{{\rm{Im}}}}\{\epsilon {E}_{i}{E}_{j}^{* }+\frac{1}{\mu }{B}_{i}^{* }{B}_{j}\}$ and ROM expressed by the momenta (75).

Hence, the Abraham-Minkowski debate influences the CMST theorem for arbitrary time-dependent fields, but not for time-harmonic (or monochromatic) electromagnetic waves. All our conclusions on these latter wavefields remain valid and unaffected by this debate.

## Conclusions

In summary, we have formulated the existence of a complex force in light-matter interactions which splits into two, either scaled or instantaneous. The real part is the standard time-averaged force, RLF, due to transfer of Poynting momentum. The imaginary part, ILF, established here, stems from the exchange of reactive (i.e., imaginary Poynting) momentum and is linked to the accretion of what we find as the reactive strength of canonical (or orbital) momentum; and that, like the reactive energy, unavoidably appears as the incident wevefield hits the object.

Within the area of nanophotonics, near-field effects and reactive quantities at the nanoscale are shown here to be of importance in connection with optical manipulation. Thus we highlight the main conclusions of this work:

1. Since the Maxwell stress tensor is the basis of electromagnetic optical forces and binding, with most current detection on time averaging, the imaginary Maxwell stress tensor, its associated reactive stress of orbital momentum, and the reactive Lorentz force established here, constitute the other side of the dynamical effects in light-matter interactions.
2. The emergence of ROM and ILF is associated to the appearance of reactive energy and reactive work, recently remarked in nanoantennas. The ILF and ROM play a hindrance role versus the standard RLF, so that a large ROM storage conveys a loss of radiative force, i.e., of RLF, and vice-versa. This is illustrated with examples and makes the ROM and ILF indirectly observable. It is quite interesting that the electric and magnetic canonical momenta are the quantities characterizing the force "reactance" constituted by the ROM, in analogy with the electric and magnetic energies defining the reactive energy. This fact emphasizes the capital role^28^ of the canonical momentum in the radiation pressure.
3. Our results show that, in absence of body losses, the internal ROM contribution to the ILF is dominant versus that of the IMST (i.e., external ROM). However, absorption dissipates the interior field energy and hence the internal ROM, so that although near resonant (e.g., plasmon) wavelengths, all reactive quantities are enhanced, the resulting resonant near-field ILF becomes not so much a hurdle to the resonant “radiated” force RLF.
4. The picture illustrated in this work is paradigmatic to the complementary roles of ROM (and ILF) and the time-averaged force, completing the whole picture of the optical force emergence. As the RLF is characterized by the flow RMST across a far-zone surface sphere, and it is independent of its radius, (in analogy with the efficiency of power radiated into the far-field), the stored ROM, and its associated ILF, are characterized by the flow IMST across a near-field sphere that surrounds the distribution of charges and currents. Therefore, the ROM acts as a force “reactance” on the body.
5. Associated with these dynamic concepts, and with the role of sources in the definition of the real and imaginary field (i.e., Poynting) momenta, is the characterization of the RLF by the imaginary orbital and spin momenta, and of the ILF by what we introduce as the reactive strength of Poynting momentum.
6. While the CMST theorem, and hence the reactive force, of general arbitrary time-dependent light waves is affected by the choice of field momentum in the context of the Minkowski-Abraham debate, we have demonstrated that in the case of time-harmonic fields, this choice has no effect neither in the reactive force nor in the ROM, and like the RMST, the IMST is that of Minkowski.
  Given the recent advances in optical manipulation at the nanoscale, increasing knowledge on the details of the generation and control of electromagnetic optical forces is continuously required. Hence these long time uncovered reactive dynamic quantities should be relevant in practice.
7. Like in the design of RF antennas and nanoantennas the radiation efficiency is increased by diminishing the reactive power and reactive work, in the context of optical manipulation one may act on the ROM and ILF in order to optimize the time-averaged optical force. So the scenario established in this study contains a novel tool to handle the mechanical action of light on matter.

Given the zero time-average of the ROM flow related to the instantaneous reactive force, their direct detection may enter the domain of femtosecond and attosecond optics on using subcycle pulse illumination^63–65^.

Although we have emphasized the nanophotonics domain, the fundamental physics of the complex Lorentz force highlights its presence in the general electrodynamics realm, and suggests its existence in the interaction of sound, fluids^66^, and other matter waves, thus opening a new landscape of possible dynamic phenomena of waves on matter.

## Supplementary information

The complex Maxwell stress tensor theorem: The imaginary stress tensor and the reactive strength of orbital momentum. A novel scenery underlying electromagnetic optical forces
