# Supplementary material for: The complex Maxwell stress tensor theorem: The imaginary stress tensor and the reactive strength of orbital momentum. A novel scenery underlying electromagnetic optical forces
Source: Light Sci Appl. 2022 Oct 12;11:297. doi: 10.1038/s41377-022-00979-2 (PMC9556612; doi:10.1038/s41377-022-00979-2)
Supplement: Supplementary file 1 — The complex Maxwell stress tensor theorem: The imaginary stress tensor and the reactive strength of orbital momentum. A novel scenery underlying electromagnetic optical forces [file 41377_2022_979_MOESM1_ESM.pdf]

Supplementary Material for "The complex Maxwell stress tensor theorem: The imaginary stress tensor and the reactive strength of orbital momentum. A novel scenery underlying electromagnetic optical forces"

Manuel Nieto-Vesperinas[1,*] and Xiaohao Xu[2,3,†]

[1]*Instituto de Ciencia de Materiales de Madrid,*
Consejo Superior de Investigaciones Científicas.
Campus de Cantoblanco, Madrid 28049, Spain. www.icmm.csic.es/mnv
[2]*State Key Laboratory of Transient Optics and Photonics,*
Xi'an Institute of Optics and Precision Mechanics,
Chinese Academy of Sciences, Xi'an 710119, China
[3] *Institute of Nanophotonics, Jinan University, Guangzhou 511443, China*

[*]Electronic address: mnieto@icmm.csic.es
[†]Electronic address: xuxhao_dakuren@163.com

APPENDIX A: REACTIVE TORQUE. A DIPOLAR PARTICLE

The time-averaged optical torque $< \mathbf{\Gamma} >= \mathbf{r} \times \mathrm{Re}\{\boldsymbol{\mathcal{F}}\}$ on the object, of lever arm $\mathbf{r}$ is:

$$< \mathbf{\Gamma} >= \int_S d^2r \, \mathbf{r} \times \mathrm{Re}\{T_{ij}\} n_j. \tag{A1}$$

In turn, the *reactive torque*: $\mathbf{\Xi} = \mathbf{r} \times \mathrm{Im}\{\boldsymbol{\mathcal{F}}\}$ reads:

$$\mathbf{\Xi} = \int_S d^2r \, \mathbf{r} \times \mathrm{Im}\{T_{ij}\} n_j + \omega \int_V d^3r \, (\mathbf{L}_m^O - \mathbf{L}_e^O). \tag{A2}$$

With the electric and magnetic time-averaged orbital angular momenta:

$$\mathbf{L}_e^O = \mathbf{r} \times \mathbf{P}_e^O \quad \mathbf{L}_m^O = \mathbf{r} \times \mathbf{P}_m^O. \tag{A3}$$

We shall call *reactive strength of orbital angular momentum* density to the quantity $\omega(\mathbf{L}_m^O - \mathbf{L}_e^O)$.

APPENDIX B: THE CANONICAL AND SPIN MOMENTA WITH SOURCES. LAGRANGIAN DERIVATION

1. The electric canonical and spin momenta

It is well-known that the electric and magnetic classical fields governed by Maxwell's equations hold dual symmetry in free-space [1]. Their lack of duality in presence of sources, i.e. electric charges and currents, has been studied by many authors who, following P.A.M. Dirac [8], postulate the existence of (so far unobserved) magnetic charges and currents that restore such symmetry, see e.g. [6, 9–12].

Without recurring to magnetic sources, deriving a Lagrangian in dual space that leads to an energy-momentum tensor from which electromagnetic quantities fulfill all conservation laws, and that yields a consistent decomposition of the energy flow (Poynting vector) into a canonical (or orbital) momentum and a spin momentum, like for electromagnetic fields in free-space [6], is problematic.

Here we introduce, notwithstanding, potentials and an energy-momentum tensor, that lead to such a possible decomposition and, hence, a characterization of the canonical and spin momenta in presence of sources.

We write the Lagrangian for the electromagnetic field $F_{\alpha\beta}$=($\mathbf{E}$,$\mathbf{H}$) with sources [13]:

$$\mathcal{L} = -\frac{1}{16\pi} F_{\alpha\beta}F^{\alpha\beta} - \frac{1}{c} j_\gamma A^\gamma. \tag{B1}$$

Greek indices run as: $0, 1, 2, 3$. Covariant and contravariant tensor indices are related by [1]: $T^{\cdots\alpha}_{\cdots} = g^{\alpha\beta}T^{\cdots}_{\cdots\beta}$; where $g^{\alpha\beta} = g_{\alpha\beta}$ is the Euclidean space metric tensor: $g^{\alpha\beta} = 0$ when $\alpha \neq \beta$, $g^{00} = 1$, $g^{11} = g^{22} = g^{33} = -1$; $g^{\alpha\beta}g_{\beta\gamma} = \delta^\alpha_\gamma$. The coordinate vector is denoted as either $x^\alpha = (ct, \mathbf{r})$, or $x_\alpha = g_{\alpha\gamma}x^\gamma = (ct, -\mathbf{r})$, so that the scalar product of two 4-vectors is $A_\gamma B^\gamma = A^0B^0 - \mathbf{A}\cdot\mathbf{B}$; and $\partial_\alpha = \frac{\partial}{\partial x^\alpha} = (\frac{\partial}{c\,\partial t}, \nabla)$, $\partial^\alpha = \frac{\partial}{\partial x_\alpha} = (\frac{\partial}{c\,\partial t}, -\nabla)$.

The current and potential 4-vectors are [13]: $j_\gamma = (c\rho, -\mathbf{J})$ and $A^\gamma = (\phi, \mathbf{A})$. Also $F^{\alpha\beta} = (-\mathbf{E}, \mathbf{H})$, $F_{\alpha\beta} = (\mathbf{E}, \mathbf{H})$. $F_{\alpha\beta} = g_{\alpha\sigma}g_{\beta\tau}F^{\sigma\tau}$. $F^{\alpha\beta} = \partial^\alpha A^\beta - \partial^\beta A^\alpha$ and $F_{\alpha\beta} = \partial_\alpha A_\beta - \partial_\beta A_\alpha$ convey $\mathbf{E} = -\frac{1}{c}\frac{\partial\mathbf{A}}{\partial t} - \nabla\phi$, $\mathbf{H} = \nabla\times\mathbf{A}$. It is well-known that the Lagrange equations associated to (B1) lead to the second pair of Maxwell equations: $\partial_\gamma F^{\alpha\gamma} = -\frac{4\pi}{c}j^\gamma$, namely: $\nabla\cdot\mathbf{E} = 4\pi\rho$ and $\nabla\times\mathbf{H} = \frac{\partial\mathbf{E}}{\partial t} + \frac{4\pi}{c}\mathbf{J}$.

As is known, the Lagrangian (B1) gives rise to the canonical energy-momentum tensor:

$$\tilde{T}^{\alpha\beta} = \partial^\alpha A^\gamma \frac{\partial\mathcal{L}}{\partial(\partial_\beta A^\gamma)} - g^{\alpha\beta}\mathcal{L} = -\frac{1}{4\pi}(\partial^\alpha A^\gamma)F^\beta_\gamma + g^{\alpha\beta}(\frac{1}{16\pi}F_{\gamma\sigma}F^{\gamma\sigma} + \frac{1}{c}j_\gamma A^\gamma). \tag{B2}$$

The electric canonical (or orbital) momentum $P_e^{O\alpha}$ of the electromagnetic field is given by the component $\tilde{T}^{\alpha 0}/c$ [6], with $\alpha \neq 0$

$$P_e^{O\alpha} = \frac{1}{c}\tilde{T}^{\alpha 0} = -\frac{1}{4\pi c}(\partial^\alpha A^\gamma)F^0_\gamma + g^{\alpha 0}(\frac{1}{16\pi c}F_{\gamma\sigma}F^{\gamma 0} + \frac{1}{c^2}j_\gamma A^\gamma) = -\frac{1}{4\pi c}(\partial^\alpha A^\gamma)F^0_\gamma,\ (\alpha \neq 0). \tag{B3}$$

I.e, since $F^0_0 = 0$ and $\alpha \neq 0$, the *ith* component of the cananonical momentum is:

$$\begin{aligned} P_e^{Oi} &= \frac{1}{c}\tilde{T}^{i0} = -\frac{1}{4\pi c}(\partial^i A^j)F^0_j = -\frac{1}{4\pi c}(\partial^i A^j)g_{jk}F^{0k} \\ &= \frac{1}{4\pi c}(\partial^i A^j)F^{0j} = \frac{1}{4\pi c}E^j\partial_i A^j,\ (i, j, = 1, 2, 3). \end{aligned} \tag{B4}$$

Henceforth being understood that latin indices run as $1, 2, 3$.

Concerning the electric spin momentum $P_e^{S\,i}$ associated to the tensor $\Delta T^{\alpha\beta}$ that added to the canonical energy-momentum tensor with sources (B2) symmetrizes it [6], we choose

$$\begin{aligned} \Delta T^{\alpha\beta} &= \partial_\gamma\psi^{\alpha\beta\gamma} + \frac{1}{c}A^\alpha j^\beta - \frac{1}{16\pi c}g^{\alpha\beta}j_\gamma A^\gamma = \frac{1}{4\pi}\partial_\gamma(A^\alpha F^{\beta\gamma}) + \frac{1}{c}A^\alpha j^\beta - \frac{1}{c}g^{\alpha\beta}j_\gamma A^\gamma. \\ \psi^{\alpha\beta\gamma} &= \frac{1}{4\pi}A^\alpha F^{\beta\gamma}, \quad \psi^{\alpha\beta\gamma} = -\psi^{\alpha\gamma\beta},\ \partial_\beta\partial_\gamma\psi^{\alpha\beta\gamma} = 0. \end{aligned} \tag{B5}$$

So that, since $\alpha \neq 0$ and $F^{00} = 0$, $P_e^{S\,i}$ would be

$$P_e^{Si} = \frac{1}{c}\Delta T^{\alpha 0} = \frac{1}{c}\Delta T^{i0} = \frac{1}{4\pi c}\partial_j(A^iF^{0j}) + \frac{1}{c}A^ij^0 = -\frac{1}{4\pi c}\partial_j(E^jA^i) + \frac{1}{c}A^ij^0,\ (i, j = 1, 2, 3). \tag{B6}$$

The sum of the canonical and spin momenta, P_e^{Oi} and P_e^{Si}, Eqs. (B4) and (B6), is that part $\mathbf{g}_e$ of the field momentum due to the electric field, i.e.

$$g_e^i = \frac{1}{4\pi c}[E^j \partial_i A^j - \partial_j(E^j A^i)] + \frac{1}{c}A^i j^0, \ (i,j=1,2,3) \tag{B7}$$

In order to make the link of (B7) with the expression (32), we consider time-harmonic fields. Then the spatial parts hold

$$\mathbf{A} = -\frac{i}{k}(\mathbf{E} + \nabla\phi), \quad \nabla\cdot\mathbf{E} = 4\pi\rho, \tag{B8}$$

After introducing the time average on the $\mathcal{O}$-operation as: $< A\mathcal{O}B >= (1/2)Re(A^*\mathcal{O}B)$, straightforward operations lead to the time-average of the electric field (i.e. Poynting) momentum

$$< \mathbf{g} >= \mathbf{P}_e^S + \mathbf{P}_e^O + \frac{1}{2\omega}\mathrm{Im}\{\rho^*\mathbf{E}\}. \tag{B9}$$

Equation (B9) is identical to Eq. (32).

Note that we did not need to introduce any choice of gauge in the 4-potential A^α. This is due to the fact that our selected $\Delta T^{\alpha\beta}$, Eq. (B5), automatically symmetrizes the energy-momentum tensor with sources, (B2), as the term $\frac{1}{c}A^\alpha j^\beta$ in (B5) cancels an identical term obtained from $\frac{1}{4\pi}\partial_\gamma(A^\alpha F^{\beta\gamma})$ and the second Maxwell equation: $\partial_\gamma F^{\beta\gamma} = -\frac{4\pi}{c}j^\beta$. Consequently, the symmetrized energy-momentum tensor results

$$T^{\alpha\beta} = \tilde{T}^{\alpha\beta} + \Delta T^{\alpha\beta} = -\frac{1}{4\pi}F^{\alpha\gamma}F_\gamma^\beta + \frac{1}{16\pi}g^{\alpha\beta}F^{\delta\sigma}F_{\delta\sigma} \ , \tag{B10}$$

which has the same functional form as the symmetric free-space energy-momentum tensor [1, 13] and, as such, it fulfils the conservation equation with sources

$$\partial_\alpha T^{\alpha\beta} = -\frac{1}{c}F^{\beta\gamma}j_\gamma. \tag{B11}$$

2. The magnetic canonical and spin momenta with sources

Within the aforementoned limitations from the lack of duality between the electric and magnetic field in presence of (non-magnetic) sources, we now pass on to addressing dual quantities. First, we quote the dual field pseudotensor: $G^{\alpha\beta} = \frac{1}{2}\epsilon^{\alpha\beta\gamma\delta}F_{\gamma\delta}$, where $\epsilon^{\alpha\beta\gamma\delta}$ denotes the fourth-order Levi-Civita completely antisymmetric tensor. This (free-field) dual pseudotensor holds: $G_{\alpha\beta} = \partial_\alpha C_\beta - \partial_\beta C_\alpha$, $\quad G^{\alpha\beta} = \partial^\alpha C^\beta - \partial^\beta C^\alpha$.

We introduce the dual fields with sources

$$G^{(s)\,\alpha\beta} = -G^{(s)\,\beta\alpha} = (-\mathbf{H}, \mathbf{E}) = (\partial^\alpha C^\beta - \partial^\beta C^\alpha) + 4\pi R^{\alpha\beta}. \tag{B12}$$

So that

$$G^{(s)}_{\alpha\beta} = (\mathbf{H}, \mathbf{E}) = (\partial_\alpha C_\beta - \partial_\beta C_\alpha) + 4\pi R_{\alpha\beta} = g_{\beta\tau} G^{(s)\,\sigma\tau}. \tag{B13}$$

The superscript (s) denotes fields in presence of sources. The dual potential 4-vector is $C^\gamma = (\theta, \mathbf{C})$, and the tensor

$$R^{\alpha\beta} = -R^{\beta\alpha} = R_{\alpha\beta} = -R_{\beta\alpha} = (\mathbf{0}, \boldsymbol{\Upsilon}), \qquad \mathbf{0} = (0, 0, 0), \qquad \frac{\partial \boldsymbol{\Upsilon}}{\partial t} = -\mathbf{J}. \tag{B14}$$

The ordering of $\mathbf{0}$ and $\boldsymbol{\Upsilon}$ in $R^{\alpha\beta}$ (or in $R_{\alpha\beta}$), Eq. (B14), is the same as that of $\mathbf{H}$ and $\mathbf{E}$ in $G^{(s)\,\alpha\beta}$, respectively; (or in $G^{(s)}_{\alpha\beta}$). The 4-vector potential is $C^\gamma = (\theta, \mathbf{C})$. Equations (B12) and (B13) mean

$$\mathbf{E} = -\nabla \times \mathbf{C} + 4\pi \boldsymbol{\Upsilon}, \qquad \mathbf{H} = -\frac{1}{c}\frac{\partial \mathbf{C}}{\partial t} - \nabla\theta \ . \tag{B15}$$

Note that introducing the Maxwell equation: $\nabla \cdot \mathbf{E} = 4\pi\rho$ into the first of Eqs. (B15) one obtains the well-known continuity equation: $\nabla \cdot \mathbf{J} + \frac{\partial \rho}{\partial t} = 0$.

It should be remarked that we introduced the tensor $R_{\alpha\beta}$ to make the fields $G_{\alpha\beta}$ (or $G^{\alpha\beta}$), written in vectorial form: $(\mathbf{H}, \mathbf{E})$ [or $(-\mathbf{H}, \mathbf{E})$] as Eqs. (B15), to fulfil the second pair of Maxwell equations with sources: $\nabla \cdot \mathbf{E} = 4\pi\rho$ and $\nabla \times \mathbf{H} = \frac{1}{c}\partial_t \mathbf{E} + \frac{4\pi}{c}\mathbf{J}$. Likewise, it is known that the vectorial form of the first pair of Maxwell equations: $\nabla \times \mathbf{E} = \frac{1}{c}\partial_t \mathbf{H}$ and $\nabla \cdot \mathbf{H} = 0$ is obtained from $F_{\alpha\beta} = \partial_\alpha A_\beta - \partial_\beta A_\alpha = (\mathbf{E}, \mathbf{H})$ (or from $F^{\alpha\beta}$) , expressed as $\mathbf{E} = -\frac{1}{c}\frac{\partial \mathbf{A}}{\partial t} - \nabla\phi$, $\mathbf{H} = \nabla \times \mathbf{A}$.

However, we note that the Lagrange equations of the Lagrangian built from $F_{\alpha\beta}$, Eq. (B1), yield the second pair of Maxwell's equations written in tensor notation: $\partial_\gamma F^{\alpha\gamma} = -\frac{4\pi}{c} j^\gamma$, but not the first pair: $\partial_\gamma G^{\alpha\gamma} = 0$. Such first pair of Maxwell equations is obtained from the dual Lagrangian without sources [1, 13]

$$\hat{\mathcal{L}} = -\frac{1}{16\pi} G_{\alpha\beta} G^{\alpha\beta}. \tag{B16}$$

This asymmetry reflects the breackdown of electromagnetic duality in absence of magnetic sources. On these grounds, even in presence of sources, we propose the source-free dual

Lagrangian (B16) rather than the one built from the pseudotensor $G^{(s)}_{\alpha\beta}$ and $G^{(s)\,\alpha\beta}$ introduced above. This Lagrangian also yields the correct energy.

In this connection it is worth observing that had one employed in (B16) $G^{(s)}_{\alpha\beta}$ and $G^{(s)\,\alpha\beta}$ given by (B12) and (B13), rather than their free-space expressions, the first pair of Maxwell equations would be obtained from such a Lagrangian if the following extra condition holds:

$$\partial_\beta R^{\alpha\beta} = 0, \tag{B17}$$

which according to (B14) means that $\nabla \times \boldsymbol{\Upsilon} = 0$, and hence $\nabla \times \mathbf{J} = \mathbf{0}$. Thus the current density $\mathbf{J}$ would be longitudinal. We believe that this is an unnecessary and little realistic restriction.

Note that (B17) was obtained from the Lagrange equations using in (B16) Eqs. (B12) and (B13) instead of $G_{\alpha\beta}$ and $G^{\alpha\beta}$, as well as the equalities: $\frac{\partial[(\partial^\alpha C^\beta - \partial^\beta C^\alpha)R_{\alpha\beta}]}{\partial(\partial^\alpha C^\beta)} = 2R_{\alpha\beta} = 2R^{\alpha\beta} = \frac{\partial[(\partial_\alpha C_\beta - \partial_\beta C_\alpha)R^{\alpha\beta}]}{\partial(\partial_\alpha C_\beta)}$ since $R^{\alpha\beta} = R_{\alpha\beta}$ and $R_{\alpha\beta} = -R_{\beta\alpha}$.

The canonical energy-momentum tensor then is

$$\tilde{\hat{T}}^{\alpha\beta} = \partial^\alpha C^\gamma \frac{\partial \mathcal{L}}{\partial(\partial_\beta C^\gamma)} - g^{\alpha\beta}\hat{\mathcal{L}} = -\frac{1}{4\pi}(\partial^\alpha C^\gamma)G^\beta_\gamma + \frac{1}{16\pi}g^{\alpha\beta}G_{\gamma\sigma}G^{\gamma\sigma}. \tag{B18}$$

The magnetic canonical momentum $\hat{P}^{O\alpha}_m$ is given by $\tilde{T}^{\alpha 0}/c$, with $\alpha \neq 0$

$$\hat{P}^{O\alpha}_m = \frac{1}{c}\tilde{\hat{T}}^{\alpha 0} = -\frac{1}{4\pi c}(\partial^\alpha C^\gamma)G^0_\gamma + \frac{1}{16\pi c}g^{\alpha 0}G_{\gamma\sigma}G^{\gamma 0} = -\frac{1}{4\pi c}(\partial^\alpha C^\gamma)G^0_\gamma, \ (\alpha \neq 0). \tag{B19}$$

And since $G^0_0 = 0$ and $\alpha \neq 0$, the *ith* component of the cananonical momentum finally is:

$$\begin{aligned}\hat{P}^{Oi}_m &= \frac{1}{c}\tilde{\hat{T}}^{i0} = -\frac{1}{4\pi c}(\partial^i C^j)G^0_j = -\frac{1}{4\pi c}(\partial^i G^j)g_{jk}G^{0k} \\ &= \frac{1}{4\pi c}(\partial^i C^j)G^{0j} = \frac{1}{4\pi c}H^j\partial_i C^j, \ (i, j, = 1, 2, 3).\end{aligned} \tag{B20}$$

As for the magnetic spin momentum $\hat{P}^{S\,i}_m$ associated to the tensor $\Delta\hat{T}^{\alpha\beta}$, we choose

$$\begin{aligned}\Delta\hat{T}^{\alpha\beta} &= \partial_\gamma\chi^{\alpha\beta\gamma} + \frac{1}{c^2}\epsilon_{\alpha\beta\gamma\delta}C^\gamma j^\delta = \frac{1}{4\pi}\partial_\gamma(C^\alpha F^{\beta\gamma}) + \frac{1}{c^2}\epsilon_{\alpha\beta\gamma\delta}C^\gamma j^\delta. \\ \chi^{\alpha\beta\gamma} &= \frac{1}{4\pi}C^\alpha F^{\beta\gamma}, \qquad \chi^{\alpha\beta\gamma} = -\chi^{\alpha\gamma\beta}, \quad \partial_\beta\partial_\gamma\chi^{\alpha\beta\gamma} = 0.\end{aligned} \tag{B21}$$

As $\alpha \neq 0$ and $G^{00} = 0$, we get

$$\hat{P}^{S\,i}_m = \frac{1}{c}\Delta\hat{T}^{\alpha 0} = \frac{1}{c}\Delta\hat{T}^{i0} = \frac{1}{4\pi c}\partial_j(C^i G^{0j}) + \frac{1}{c^2}\epsilon_{i0kl}C^k j^l = -\frac{1}{4\pi c}\partial_j(H^j C^i) + \frac{1}{c^2}\epsilon_{ikl}C^k j^l, \ ) \tag{B22}$$

The sum of the canonical and spin magnetic momenta, $\hat{P}_m^{Oi}$ and $\hat{P}_m^{Si}$, Eqs. (B20) and (B22), is that part $\mathbf{g}_m$ of the Poynting momentum due to the magnetic field, namely

$$g_m^i = \frac{1}{4\pi c}[H^j \partial_i C^j - \partial_j (H^j C^i)] + \frac{1}{c^2}\epsilon_{ikl} C^k j^l, \quad (i, j, k, l = 1, 2, 3). \tag{B23}$$

Equation (B23) coincides with (33) for time-harmonic fields. Note that then the spatial parts hold

$$\mathbf{C} = -\frac{i}{k}(\mathbf{H} + \nabla\theta), \quad \nabla \cdot \mathbf{H} = 0, \tag{B24}$$

As before, after introducing the time average: $< A\mathcal{O}B >= (1/2)Re(A^*\mathcal{O}B)$, it is straightforward to obtain

$$< \mathbf{g} >= \mathbf{P}_m^S + \mathbf{P}_m^O - \frac{1}{2kc^2}\text{Im}\{\mathbf{J}^* \times \mathbf{H}\}. \tag{B25}$$

It should be remarked, however, that the electromagnetic duality breakdown requires that the fields $G^{\alpha\beta}$ in the tensor $\Delta\hat{T}^{\alpha\beta}$, and its corresponding magnetic spin momentum $\hat{P}_m^{S\,i}$, meet some conditions. One is that $C^\alpha = (0, \mathbf{C})$. I.e. $\theta = 0$. Then:

$$\mathbf{H} = -\frac{1}{c}\frac{\partial \mathbf{C}}{\partial t}. \tag{B26}$$

So that $\nabla \cdot \partial_t \mathbf{C} = 0$; and if we chose the Lorenz condition $\partial_\gamma C^\gamma = 0$, one would also have $\nabla \cdot \mathbf{C} = 0$. This is equivalent to a Coulomb gauge in the space of dual quantities. However in this space $\nabla\theta = 0$ does not mean $\rho = 0$ since $\nabla \cdot \mathbf{E} = 4\pi\nabla \cdot \mathbf{\Upsilon} = 4\pi\rho$, and hence $\nabla \cdot \mathbf{J} = -\partial_t \rho$. Moreover, using (B15) and the first Maxwell equation: $\nabla \times \mathbf{E} = -\frac{1}{c}\partial_t \mathbf{H}$ we get

$$\nabla^2 \mathbf{C} - \frac{1}{c^2}\frac{\partial^2 \mathbf{C}}{\partial t^2} = -4\pi\nabla \times \mathbf{\Upsilon}, \tag{B27}$$

which indicates that the source function of the wave equation for $\mathbf{C}$ is transversal. In the $F_{\alpha\beta}$-space, Coulomb's gauge involves the source in Eq. (B27) for $\mathbf{A}$ being a transversal current, and the charge is associated to a longitudinal current which acts as a source of static (i.e. near) field. This does not happen in the dual space, however, where nothing indicates that the current density associated to the charge be longitudinal.

On the other hand, although the source vector of the wave equation of $\mathbf{C}$ is transversal, it does not coincide with a transversal current density. These features of the potential C^γ in the space of dual fields $G_{\alpha\gamma}$ are in clear contrast with that of the potential A^γ in the space of $F_{\alpha\gamma}$.

Another aspect of the tensor $\Delta\hat{T}^{\alpha\beta}$ is that, added to the canonical dual tensor $\tilde{\hat{T}}^{\alpha\beta}$, it does not yield a symmetrized energy momentum tensor, in contrast with $\Delta T^{\alpha\beta}$, nor its divergence is zero. (Notice in this connection that neither the tensor $\Delta T^{\alpha\beta}$ of Eq. (B5) posesses a zero divergence as it should unless $\partial_\gamma(j_\beta A^\beta) = 0$). These again are other symptoms of the electromagnetic duality breackdown in the presence of sources, and indicate that concerning symmetry of $\tilde{\hat{T}}^{\alpha\beta}$ and null divergence of $\Delta T^{\alpha\beta}$ and $\Delta\tilde{T}^{\alpha\beta}$, there might be different choices for these tensors, and even for the dual Lagrangian. We have selected here those that we were more strightforwardly able to find among those leading to the canonical and spin momenta of the electromagnetic field within a covariant formulation.

APPENDIX C: THE IMAGINARY MAXWELL STRESS TENSOR AND THE ANGULAR SPECTRUM OF THE FIELDS

First, we expand the fields into their angular spectra of plane waves [3, 14]:

$$\mathbf{E}(\mathbf{r}) = \int_{-\infty}^{\infty} d^2\mathbf{K}\; \mathbf{e}(\mathbf{K}) \exp[i(\mathbf{K}\cdot\mathbf{R} + k_z z)]. \tag{C1}$$

Where $\mathbf{r} = (\mathbf{R}, z)$, $\mathbf{R} = (x, y)$, $\mathbf{k} = (\mathbf{K}, k_z)$, $\mathbf{K} = (K_x, K_y)$, $|\mathbf{k}|^2 = k^2$.

$$k_z = \sqrt{k^2 - K^2} = q_h\,,\ K \leq k,\ (\textit{propagating waves}).$$
$$k_z = i\sqrt{K^2 - k^2} = iq_e\,,\ K > k,\ (\textit{evanescent waves}). \tag{C2}$$

And an analogous expansion for $\mathbf{B}(\mathbf{r})$. Therefore, by splitting the field angular spectrum integral into homogeneous and evanescent components, (with susbscript h and e, respectively),

$$\begin{aligned}\int_{-\infty}^{\infty} d^2\mathbf{R}(\mathbf{P}_m^S - \mathbf{P}_e^S) = {}& \frac{1}{16\pi\omega}\int_{-\infty}^{\infty} d^2\mathbf{R}\int_{K\leq k} d^2\mathbf{K}\int_{K'\leq k} d^2\mathbf{K}'\,\mathrm{Im}\{\exp[-i(\mathbf{K}-\mathbf{K}')\cdot\mathbf{R} + (q_h - q_h')z)]\\ &\times[-i(\mathbf{K}-\mathbf{K}', q_h - q_h')] \times [\mathbf{b}_h^*(\mathbf{K}) \times \mathbf{b}_h(\mathbf{K}') - \mathbf{e}_h^*(\mathbf{K}) \times \mathbf{e}_h(\mathbf{K}')]\}\\ &+\frac{1}{16\pi\omega}\int_{-\infty}^{\infty} d^2\mathbf{R}\int_{K>k} d^2\mathbf{K}\int_{K'>k} d^2\mathbf{K}'\,\mathrm{Im}\{\exp[-i(\mathbf{K}-\mathbf{K}')\cdot\mathbf{R} + (q_e + q_e')z)]\\ &\times[-i(\mathbf{K}-\mathbf{K}'), -(q_e + q_e')] \times [\mathbf{b}_e^*(\mathbf{K}) \times \mathbf{b}_e(\mathbf{K}') - \mathbf{e}_e^*(\mathbf{K}) \times \mathbf{e}_e(\mathbf{K}')]\}. \end{aligned} \tag{C3}$$

Making $z = z_0 \geq 0$, the $\mathbf{R}$-integration yields $(2\pi)^2\delta(\mathbf{K}' - \mathbf{K})$. Then, after performing the $\mathbf{K}'$-integral and expressing $\mathbf{b}_\perp(\mathbf{K}) = (b_x(\mathbf{K}), b_y(\mathbf{K}), 0)$, $\mathbf{e}_\perp(\mathbf{K}) = (e_x(\mathbf{K}), e_y(\mathbf{K}), 0)$, we inmediately see that the the homogeneous (propagating) part of (C3) is zero. Therefore the

above becomes

$$\int_{-\infty}^{\infty} d^2\mathbf{R}(\mathbf{P}_m^S - \mathbf{P}_e^S) = \frac{\pi}{2\omega}\int_{K>k} d^2\mathbf{K}\,\exp(-2q_e z_0)\mathrm{Im}\{[0,0,-q_e]\times[\mathbf{b}_e^*(\mathbf{K})\times\mathbf{b}_e(\mathbf{K}) - \mathbf{e}_e^*(\mathbf{K})\times\mathbf{e}_e(\mathbf{K})]\}$$

$$= \frac{\pi}{\omega}\int_{K>k} d^2\mathbf{K}\, q_e \exp(-2q_e z_0)\mathrm{Im}\{b_{e\,z}^*(\mathbf{K})\mathbf{b}_{e\perp}(\mathbf{K}) - e_{e\,z}^*(\mathbf{K})\mathbf{e}_{e\perp}(\mathbf{K})\}. \quad \text{(C4)}$$

In particular, we may take $z_0 = 0$.

APPENDIX D: THE COMPLEX MAXWELL STRESS TENSOR ON A DIPOLE

Addressing the complex Maxwell stress tensor theorem, Eq. (17), in the scattering from a magnetoelectric dipole or particle, we split the fields into incident $\mathbf{E}^{(i)}$, $\mathbf{B}^{(i)}$ and scattered $\mathbf{E}^{(s)}$, $\mathbf{B}^{(s)}$: $\mathbf{E} = \mathbf{E}^{(i)} + \mathbf{E}^{(s)}$, $\mathbf{B} = \mathbf{B}^{(i)} + \mathbf{B}^{(s)}$. The dipole scattered fields are

$$\mathbf{E}^{(s)}(\mathbf{r}) = \frac{k^2}{\epsilon}[\mathbf{n}\times(\mathbf{p}\times\mathbf{n})]\frac{e^{ikr}}{r} + \frac{1}{\epsilon}[3\mathbf{n}(\mathbf{n}\cdot\mathbf{p}) - \mathbf{p}](\frac{1}{r^3} - \frac{ik}{r^2})e^{ikr}$$
$$-\sqrt{\frac{\mu}{\epsilon}}(\mathbf{n}\times\mathbf{m})(\frac{k^2}{r} + \frac{ik}{r^2})e^{ikr}. \qquad (\mathbf{r} = r\mathbf{n}). \quad \text{(D1)}$$

$$\mathbf{B}^{(s)}(\mathbf{r}) = \mu k^2[\mathbf{n}\times(\mathbf{m}\times\mathbf{n})]\frac{e^{ikr}}{r} + \mu[3\mathbf{n}(\mathbf{n}\cdot\mathbf{m}) - \mathbf{m}](\frac{1}{r^3} - \frac{ik}{r^2})e^{ikr}$$
$$+\sqrt{\frac{\mu}{\epsilon}}(\mathbf{n}\times\mathbf{p})(\frac{k^2}{r} + \frac{ik}{r^2})e^{ikr}. \qquad (\epsilon = \mu = 1). \quad \text{(D2)}$$

We assume the surrounding medium to be vacuum or air, so that $\epsilon = \mu = 1$. Introducing the above splitting into the first term of (17), one obtains for the non-zero terms

$$\int_{\partial V} d^2r\, T_{ij}n_j = \frac{1}{8\pi}\int_{\partial V} d^2r\{(\mathbf{E}^{(i)*}\cdot\mathbf{n})\mathbf{E}^{(i)} + (\mathbf{B}^{(i)}\cdot\mathbf{n})\mathbf{B}^{(i)*}$$
$$+(\mathbf{E}^{(i)*}\cdot\mathbf{n})\mathbf{E}^{(s)} + (\mathbf{E}^{(s)*}\cdot\mathbf{n})\mathbf{E}^{(i)} + (\mathbf{B}^{(i)}\cdot\mathbf{n})\mathbf{B}^{(s)*} + (\mathbf{B}^{(s)}\cdot\mathbf{n})\mathbf{B}^{(i)*}$$
$$+[(\mathbf{E}^{(s)*}\cdot\mathbf{n})\mathbf{E}^{(s)} + (\mathbf{B}^{(s)}\cdot\mathbf{n})\mathbf{B}^{(s)*}] - \mathrm{Re}\{\mathbf{E}^{(i)*}\cdot\mathbf{E}^{(s)} + \mathbf{B}^{(i)}\cdot\mathbf{B}^{(s)*}\} + \frac{1}{2}(|\mathbf{E}^{(s)}|^2 + |\mathbf{B}^{(s)}|^2)]\mathbf{n}\}. \quad \text{(D3)}$$

When one takes the real part of (D3) which yields the time-averaged force $<\boldsymbol{\mathcal{F}}>$, the integral (D3) of the first two terms with the incident field only, is zero; while the last two terms of this real part of (D3) are well-known [4, 16] to yield the electric-magnetic dipole interference force $<\mathbf{F}_{em}> = -\frac{k^4}{3}\mathrm{Re}\{\mathbf{p}\times\mathbf{m}^*\}$. Furthermore, since this real part is independent of the integration contour, by choosing ∂V to be a sphere of large radius r such that $kr\to\infty$, there is no contribution of the fourth, sixth, seventh and eight terms since the scattered field in this region of S is known to be transversal to $\mathbf{n}$. In addition, on this large surface ∂V one inmediately sees by applying Jones' lemma based on the principle of

the stationary phase [2, 4], that the contribution of the mixed incident-scattered field third and fifth terms is also zero since $\mathbf{n}$ becomes equal to the incident wavevector, with respect to which the incident field is transversal.

Therefore, only the diagonal last four terms, which belong to the real part of the CMST, contribute to $<\boldsymbol{\mathcal{F}}>$. This is the reason by which we emphasize through the text that the far-field flux IMST is zero. In fact, taking the imaginary part of (D3) in the far-zone one obtains that all terms, one by one, are zero. This has important consequences for the total spin momentum, as shown in in the section on dipoles of the main text.

Taking the imaginary part of (D3), which depends on the choice of the integration surface ∂V, the first integral involving only $\mathbf{E}^{(i)}$ and $\mathbf{B}^{(i)}$ is cancelled out by the incident reactive orbital momentum, even if the body is illuminated by an evanescent wave [cf. Eq. (48)]. We take as the dipole volume and its boundary: V_0 and ∂V_0, respectively. They correspond to the smallest sphere of radius a that encloses the dipole; or if this is a particle, ∂V_0 is the limiting outside sphere circunscribed to its physical surface. The contribution of the non-diagonal terms involving the scattered field yields

$$\frac{1}{8\pi}\mathrm{Im}\{\int_{\partial V_0} d^2r[(\mathbf{E}^{(s)*}\cdot\mathbf{n})\mathbf{E}^{(s)}+(\mathbf{B}^{(s)}\cdot\mathbf{n})\mathbf{B}^{(s)*}]\}=0. \tag{D4}$$

We note that the left side of (D4) is: $-\frac{2k}{3a^3}\mathrm{Re}\{\mathbf{p}^*\times\mathbf{m}\}$ when the r^{-1} dependent far-zone terms are excluded from $\mathbf{E}^{(s)}$ and $\mathbf{B}^{(s)}$ in (D1) and (D2).

Since the dipole is small versus the wavelength, we work with $\mathbf{E}^{(i)}(\mathbf{r})$ and $\mathbf{B}^{(i)}(\mathbf{r})$ expanded into a Taylor series around the origin of coordinates which coincides with the dipole center:

$$\mathbf{E}^{(i)}(\mathbf{r})=\mathbf{E}^{(i)}(0)+r[(\mathbf{n}\cdot\nabla)\mathbf{E}^{(i)}(\mathbf{r})]_{r=0}\,,\quad \mathbf{B}^{(i)}(\mathbf{r})=\mathbf{B}^{(i)}(0)+r[(\mathbf{n}\cdot\nabla)\mathbf{B}^{(i)}(\mathbf{r})]_{r=0}\,. \tag{D5}$$

On surface integration of (D3) there appear terms with factors $k^2a^2\exp(\mp ika)$, $(1\pm ika)\exp(\mp ika)$ and $(k^2a^2\mp ika)\exp(\mp ika)$. They stem from those $(k^2/r)\exp(\mp ikr)$, $(1/r^3\pm ik/r^2)\exp(\mp ikr)$ and $(k^2/r\mp ik/r^2)\exp\mp(ikr)$, respectively, in the scattered fields (D1) and (D2). In compatibility with (D5) and in order to obtain for the real part of (D3) the correct well-known expression [4] which is a quantity independent of a, (since we know that this real part should not depend on the integration contour), we should take these three factors as 0, 1 and 1, respectively. Further studies should confirm, however, whether this always holds for the imaginary part of (D3), or whether one should include the full above a-factors in evaluating this imaginary part.

In order to illustrate how the terms of (D3) involving incident and scattered fields are evaluated, we show as an example the calculation of the term

$$-\frac{1}{8\pi}\mathrm{Re}\int_{\partial V_0} d^2r \mathrm{Re}(\mathbf{E}^{(i)\,*}\cdot\mathbf{E}^{(s)})\mathbf{n} = -\frac{1}{8\pi}\mathrm{Re}\int_{\partial V_0} d^2r(\mathbf{E}^{(i)\,*}(0)+r[(\mathbf{n}\cdot\nabla)\mathbf{E}^{(i)\,*}(\mathbf{r})]_{\mathbf{r}=0}\cdot\mathbf{E}^{(s)})\mathbf{n}. \quad \text{(D6)}$$

Using spherical coordinates, $d^2r = r^2 d\phi\sin\theta d\theta$, $\mathbf{n} = (\sin\theta\cos\phi, \sin\theta\sin\phi, \cos\theta)$. The part of $\mathbf{E}^{(s)}$ that contributes to the force on the electric dipole and yields a non-zero integral in (D6) reduces on using a framework such that $\mathbf{p} = (0,0,p)$ in (D1), to

$$\begin{aligned}
-\frac{1}{8\pi}\mathrm{Re}\int_{\partial V_0} d^2r\mathrm{Re}(\mathbf{E}^{(i)\,*}\cdot\mathbf{E}^{(s)})\mathbf{n} &= -\frac{1}{8\pi}\mathrm{Re}\int_0^{2\pi} a^2(\frac{1}{a^3}-\frac{ik}{a^2})\exp(ika)d\phi\sin\theta \\
\times\int_0^{\pi} d\theta(\sin\theta\cos\phi, &\sin\theta\sin\phi,\cos\theta)\{[3p\cos\theta(\sin\theta\cos\phi,\sin\theta\sin\phi,\cos\theta) \\
-(0,0,p)]\cdot[1+a(\sin\theta\cos\phi\,\partial_x &+\sin\theta\sin\phi\,\partial_y+\cos\theta\,\partial_z)](E^*_{i\,x}(\mathbf{r}),E^*_{i\,y}(\mathbf{r}),E^*_{i\,z}(\mathbf{r})\}_{r=a} \\
&= -\frac{1}{8\pi}\mathrm{Re}\{(1-ika)\exp(ika)\frac{4\pi p}{5}\,[\partial_z E^*_{i\,x}(0)+\partial_x E^*_{i\,z}(0)-\frac{5}{3}\partial_x E^*_{i\,z}(0)\,, \\
\partial_z E^*_{i\,y}(0)\ +\partial_y E^*_{i\,z}(0) &-\frac{5}{3}\partial_y E^*_{i\,z}(0),\,\partial_x E^*_{i\,x}(0)+\partial_y E^*_{i\,y}(0)+(3-\frac{5}{3})\partial_z E^*_{i\,z}(0)]\} \\
=\frac{1}{5}\mathrm{Re}\{\,p[\frac{1}{3}\partial_x E^*_{i\,z}(0) &-\frac{1}{2}\partial_z E^*_{i\,x}(0)\,,\frac{1}{3}\partial_y E^*_{i\,z}(0)-\frac{1}{2}\partial_z E^*_{i\,y}(0)\,,-\frac{1}{6}\partial_z E^*_{i\,z}(0)]\}. \qquad \text{(D7)}
\end{aligned}$$

In all above expressions, and in subsequent calculations, we employ the shortened notation: $\partial_k E_{il}(0)$ for $[\partial_k E^{(i)}_l(\mathbf{r})]_{\mathbf{r}=0}$, $(k,l = x,y,z)$. Notice that we considered $ka = 0$ in obtaining the last line of (D7) by shrinking V_0 to its center point. This is justified for the RMST only, since its integration is independent of the sphere size.

By analogous calculations with the other terms of (D3) contributing to the field scattered by the electric dipole, one gets

$$\begin{aligned}
\frac{1}{8\pi}\int_{\partial V_0} d^2r(\mathbf{E}^{(i)\,*}\cdot\mathbf{n})\mathbf{E}^{(s)} &= \\
\frac{p}{10}(1-ika)\exp(ika)[\partial_x E^*_{i\,z}(0)\ +\partial_z E^*_{i\,x}(0)\,,&\partial_y E^*_{i\,z}(0)\ +\partial_z E^*_{i\,y}(0)\,,2\partial_z E^*_{i\,z}(0)\,] \\
-\frac{p}{30}k^2a^2\exp(ika)[\partial_z E^*_{ix}+\partial_x E^*_{iz}\,,&\partial_z E^*_{iy}+\partial_y E^*_{iz}\,,2\partial_z E^*_{iz}\,]\,, \qquad \text{(D8)}
\end{aligned}$$

The second term of (D8), with the factor k^2a^2, is the contribution of the radiative part of $\mathbf{E}^{(s)}$. Also,

$$\frac{1}{8\pi}\int_{\partial V_0} d^2r(\mathbf{E}^{(s)\,*}\cdot\mathbf{n})\mathbf{E}^{(i)} = \frac{p^*}{3}(1+ika)\exp(-ika)\partial_z[E_{i\,x}(0)\,,E_{i\,y}(0)\,,E_{i\,z}(0)\,]. \quad \text{(D9)}$$

In addition, in the magnetic part of the complex MST there is the term of (D2) contributing to the magnetic field scattered by the induced electric dipole $\mathbf{p}$, which is that of the

intermediate-field region: $\frac{ike^{ikr}}{r^2}\mathbf{n}\times\mathbf{p}$. By using the Maxwell equation $\nabla\times\mathbf{E}=ik\mathbf{B}$ it yields

$$-\frac{1}{8\pi}\int_{\partial V_0} d^2r\mathrm{Re}\{\mathbf{B}^{(i)}\cdot\mathbf{B}^{(s)\,*}\}\mathbf{n}=\mathrm{Re}\{\frac{p}{6}(\partial_x E^*_{i\,z}(0)-\partial_z E^*_{i\,x}(0)\,,\partial_y E^*_{i\,z}(0)-\partial_z E^*_{i\,y}(0),0)\},\tag{D10}$$

and

$$\frac{1}{8\pi}\int_{\partial V_0} d^2r(\mathbf{B}^{(i)}\cdot\mathbf{n})\mathbf{B}^{(s)\,*}=$$

$$\frac{p^*}{6}(k^2a^2-ika)\exp(-ika)(\partial_x E_{i\,z}(0)-\partial_z E_{i\,x}(0)\,,\partial_y E_{i\,z}(0)-\partial_z E_{i\,y}(0),0).\tag{D11}$$

Adding (D7) - (D11), after taking their real part, expressing $\mathbf{p}$ with arbitrary Cartesian components p_j, $(j=1,2,3)$, one gets making $ka=0$ in (D8) and (D9), as well as the ka-factor equal to one in (D11), and dropping the subscript i of the incident field

$$<\boldsymbol{\mathcal{F}}_k>=\frac{1}{2}\mathrm{Re}\{p_j\,\partial_k E^*_j\},\qquad (j,k=1,2,3),\tag{D12}$$

Equation (D12) is the well-known time-averaged force on an electric dipole [4, 15]. The corresponding force on the magnetic dipole is obtained in an analogous way. Then the above near-field calculation yields the expression for the time-averaged force on a magnetoelectric dipole, which is well-known [4, 16],

$$<\boldsymbol{\mathcal{F}}_k>=\frac{1}{2}\mathrm{Re}\{p_j\,\partial_k E^*_j+m_j\,\partial_k B^*_j\}-\frac{k^4}{3}\mathrm{Re}[\mathbf{p}\times\mathbf{m}^*]_k\;.\qquad (j,k=1,2,3),\tag{D13}$$

On the other hand, taking the imaginary part in (D3), and using (D8) - (D11), one obtains on the electric dipole

$$\mathrm{Im}\{\int_{\partial V_0} d^2r\,T^{(mix)}_{kj}(\mathbf{p})n_j\}=\mathrm{Im}\{[\frac{1}{10}(1-ika)-\frac{1}{30}k^2a^2]\exp(ika)\,p_j\,[\partial_k E^*_j+\partial_j E^*_k]$$
$$+\frac{1}{3}(1+ika)\exp(-ika)\,p^*_j\partial_j E_k+\frac{1}{6}(k^2a^2-ika)\exp(-ika)p^*_j(\partial_k E_j-\partial_j E_k)\}\,.\;(j,k=1,2,3).\tag{D14}$$

Where we have denoted as $T^{(mix)}_{kj}(\mathbf{p})$ that part of the CMST, Eq. (D3), that uniquely involves the electric dipole moment $\mathbf{p}$. The superscript (mix) indicates that only interferences incident-scattered field contribute to the IMST

Equation (D14) is the flow IMST on an electric dipole. One approximation that simplifies (D14) is to consider all parenthesis factors equal to 1 and the term, which comes from the far-field, with the $-(1/30)k^2a^2$ factor as $-1/30$; so that one gets an expression independent of a.

$$\mathrm{Im}\{\int_{\partial V_0} d^2r\,T^{(mix)}_{kj}(\mathbf{p})n_j\}=-\frac{1}{10}\mathrm{Im}\{[p_j\,\partial_k E^*_j+p_j\,\partial_j E^*_k]\}.\qquad (j,k=1,2,3).\tag{D15}$$

Alternatively, one would obtain an expression akin to (D15), but with a factor $-1/15$ instead of $-1/10$, by neglecting in (D14) the term with the $-(1/30)k^2a^2$. This is a plausible choice after making all parenthesis of (D14) equal to one, which amounts to take $ka \simeq 0$; and hence there would be no clear justification of (D15). However we should say that a similar question appears in the derivation of (D12) and (D13) for the RLF that we know are correct.

The imaginary part of the surface integral $\mathrm{Im}\{\int_{\partial V_0} d^2r\, T_{kj}^{(mix)}(\mathbf{m})n_j\}$ is derived in an analogous way, yielding an expression like (D14) and (D15) with $\mathbf{m}^*$ and $\mathbf{B}$ replacing $\mathbf{p}$ and $\mathbf{E}^*$, respectively. Then the simplified version according to (D15) of the sum $\mathrm{Im}\{\int_{\partial V_0} d^2r\, T_{kj}^{(mix)}(\mathbf{p})n_j\} + \mathrm{Im}\{\int_{\partial V_0} d^2r\, T_{kj}^{(mix)}(\mathbf{m})n_j\}$ is

$$\mathrm{Im}\{\int_S d^2r\, T_{kj}^{(mix)} n_j\} = \frac{1}{8\pi}\int_V d^3r\, \nabla\cdot \mathrm{Im}\{E_k E_j^* + B_k^* B_j\} =$$
$$-\frac{1}{10}\mathrm{Im}[p_j\,\partial_k E_j^* + p_j\,\partial_j E_k^* - m_j\,\partial_k B_j^* - m_j\,\partial_j B_k^*]\}, \qquad (j,k=1,2,3). \tag{D16}$$

The sum of (D14) and the analogous for $\{T_{kj}^{(mix)}(\mathbf{m})n_j\}$, or its simplified version (D16) (with a factor 1/15 rather than 1/10 if the field radiative part of r^{-1} dependence is excluded), constitutes the proof of the IMST of Eq. (51).

APPENDIX E: A HEURISTIC OBTENTION OF THE COMPLEX FORCE FROM A TIME-HARMONIC FIELD ON AN ELECTRIC AND A MAGNETIC DIPOLE

1. Electric dipole

We address the complex force from a time-harmonic electromagnetic field whose analytic signals are $\boldsymbol{\mathcal{E}}(\mathbf{r},t) = \mathbf{E}(\mathbf{r})\exp(-i\omega t)$, $\boldsymbol{\mathcal{B}}(\mathbf{r},t) = \mathbf{B}(\mathbf{r})\exp(-i\omega t)$ on an electric dipole at $\mathbf{r}=\mathbf{0}$ of moment $\boldsymbol{\mathcal{P}}(\mathbf{0},t) = \mathbf{p}\exp(-i\omega t)$, $\mathbf{p} = \alpha_e \mathbf{E}(\mathbf{0})$, $[\boldsymbol{\mathcal{J}}(\mathbf{r},t) = \mathbf{J}(\mathbf{r})\exp(-i\omega t) = d\boldsymbol{\mathcal{P}}/dt$, $\mathbf{J}(\mathbf{r}) = -i\omega\mathbf{p}\delta(\mathbf{r})]$. In absence of magnetic charges, following [15] we tentatively write:

$$\boldsymbol{F}_i = \frac{1}{2}[(\mathbf{p}^*\cdot\nabla)\mathbf{E} + ik\mathbf{p}^*\times\mathbf{B}]_i = \frac{1}{2}[\alpha_e E_j{}^*\partial_j E_i + ik\alpha_e^*\epsilon_{ijk}E_j{}^* B_k], \quad (i,j,k=1,2,3). \tag{E1}$$

Since $B_k = -\frac{i}{k}\epsilon_{klm}\partial_l E_m$ and $\epsilon_{ijk}\epsilon_{klm} = \delta_{il}\delta_{jm} - \delta_{im}\delta_{jl}$, Eq. (E1) of the complex force becomes

$$\boldsymbol{F}_i = \frac{1}{2}\alpha_e^* E_j{}^*\partial_i E_j, \qquad (i,j=1,2,3). \tag{E2}$$

Whose real and imaginary parts are: the well-known expression of the time-averaged force on an electric dipolar particle [15]:

$$< \boldsymbol{F}_i >= \frac{1}{2}\text{Re}\{\alpha_e E_j \partial_i E_j^*\}, \tag{E3}$$

and

$$\text{Im}\{\boldsymbol{F}_i\} = -\frac{1}{2}\text{Im}\{\alpha_e E_j \partial_i E_j^*\}, \tag{E4}$$

that we suggest might rule the imaginary force.

2. Magnetic dipole

There exist two possible expressions to be obtained in the study of the RLF on a magnetic dipole of moment $\mathbf{m}(\mathbf{r}, t)$, depending on whether one models it as a close loop of electric current (*cld*) or as a Gilbert dipole (*mcd*) due to positive and negative magnetic charges [7, 17, 18].

In the first case the time-dependent complex force exerted by a wavefield of analytic signals $\boldsymbol{\mathcal{E}}(\mathbf{r}, t)$, $\boldsymbol{\mathcal{B}}(\mathbf{r}, t)$ on a magnetic dipole at $\mathbf{r} = \mathbf{0}$ of moment $\boldsymbol{\mathcal{M}}(\mathbf{r}, t) = \mathbf{m}(\mathbf{r}) \exp(-i\omega t) = \alpha_m \boldsymbol{\mathcal{B}}(\mathbf{r})$, that we suggest following [17] for the RLF, is:

$$\boldsymbol{\mathcal{F}}_{cld}(\mathbf{r}, t) = \frac{1}{2}[\nabla(\boldsymbol{\mathcal{M}}^* \cdot \boldsymbol{\mathcal{B}}) - \frac{1}{c}\frac{\partial}{\partial t}(\boldsymbol{\mathcal{M}}^* \times \boldsymbol{\mathcal{E}})], \tag{E5}$$

where $\frac{1}{c}(\boldsymbol{\mathcal{M}}^* \times \boldsymbol{\mathcal{E}})$ is the analogous, in terms of the analytic signals associated to the fields, of.Shockley's *hidden momentum* [19]

Expanding the first term and using Maxwell's equation: $\nabla \times \boldsymbol{\mathcal{B}} = \partial\boldsymbol{\mathcal{E}}/\partial t + \frac{c}{4\pi}\boldsymbol{\mathcal{J}}$, Eq. (E5) acquires the form

$$\boldsymbol{\mathcal{F}}_{cld}(\mathbf{r}, t) = \boldsymbol{\mathcal{F}}_{mcd} + \frac{2\pi}{c}\boldsymbol{\mathcal{M}}^* \times \boldsymbol{\mathcal{J}}, \tag{E6}$$

where

$$\boldsymbol{\mathcal{F}}_{mcd}(\mathbf{r}, t) = \frac{1}{2}[(\boldsymbol{\mathcal{M}}^* \cdot \nabla)\boldsymbol{\mathcal{B}} - \frac{1}{c}\frac{\partial \boldsymbol{\mathcal{M}}^*}{\partial t} \times \boldsymbol{\mathcal{E}}] \tag{E7}$$

is the complex force on a Gilbert dipole in terms of the analytic signals. Therefore Eq.(E6) is the relationship between the forces in the two models, *cld* and *mcd*, of the magnetic dipole.

Now, the complex force $\boldsymbol{\mathcal{F}}_{cld}$ from a *time-harmonic* field $\boldsymbol{\mathcal{E}}(\mathbf{r},t) = \mathbf{E}(\mathbf{r})\exp(-i\omega t)$, $\boldsymbol{\mathcal{B}}(\mathbf{r},t) = \mathbf{E}(\mathbf{r})\exp(-i\omega t)$ on a *cld* magnetic dipole of moment $\boldsymbol{\mathcal{M}}(\mathbf{0},t)$ is, since then the second term of (E5) is zero,

$$\boldsymbol{\mathcal{F}}_{cld} = \frac{1}{2}[\nabla(\mathbf{m}^* \cdot \mathbf{B}) + \mathbf{m}^* \times (\nabla \times \mathbf{B})]. \tag{E8}$$

Where the $\mathbf{r}$-dependence of all quantities in (E8) is implicit. Expanding the double vector product of the second term and proceeding as in Appendix E.1, we obtain

$$(\boldsymbol{\mathcal{F}}_{cld})_i = \frac{1}{2} m_j^* \partial_i B_j, \quad (i,j = 1,2,3). \tag{E9}$$

Whose real part is the well-known time-averaged force on a purely magnetic dipole [5, 16]:

$$< \boldsymbol{\mathcal{F}}_{cld} >_i = \frac{1}{2}\mathrm{Re}\{m_j \partial_i {B_j}^*\}. \tag{E10}$$

And whose imaginary part is our tentatively proposed reactive force on a magnetic dipole,

$$\mathrm{Im}\{\boldsymbol{\mathcal{F}}_{cld}\}_i = -\frac{1}{2}\mathrm{Im}\{m_j \partial_i {B_j}^*\}, \quad m_j(\mathbf{0}) = \alpha_m B_j(\mathbf{0}), \quad (i,j = 1,2,3). \tag{E11}$$

On the other hand, the complex force of this time-harmonic wavefield on a Gilbert dipole of magnetic charge current density $\boldsymbol{\mathcal{J}}(\mathbf{r},t) = \mathbf{J}_{mc}(\mathbf{r})\exp(-i\omega t) = d\boldsymbol{\mathcal{M}}/dt$, $\mathbf{J}_{mc}(\mathbf{r}) = -i\omega \mathbf{m}\delta(\mathbf{r})$, is from (E7):

$$\boldsymbol{\mathcal{F}}_{mcd} = \frac{1}{2}[(\mathbf{m}^* \cdot \nabla)\mathbf{B} - ik\mathbf{m}^* \times \mathbf{E}] = \frac{1}{2}[(\mathbf{m}^* \cdot \nabla)\mathbf{B} + \mathbf{m}^* \times (\nabla \times \mathbf{B}) - \frac{4\pi}{c}\mathbf{m}^* \times \mathbf{J}_{mc}]. \tag{E12}$$

Where the Maxwell equation for $\nabla \times \mathbf{B}$ has been used to eliminate $\mathbf{E}$. Then, expressing the electric current density as: $\mathbf{J}_{mc} = -i\omega \mathbf{p}\delta(\mathbf{r})$ and proceeding as before, we obtain:

$$(\boldsymbol{\mathcal{F}}_{mcd})_i = \frac{1}{2} m_j^* \partial_i B_j + 2i\pi k \delta(\mathbf{r})(\mathbf{m}^* \times \mathbf{p})_i. \tag{E13}$$

Notice that since $\int_{-\infty}^{\infty} d^3\mathbf{r}\delta(\mathbf{r}) = 1$, the second term of (E13) has, like the first term, spatial dimension L^{-2}. Also we note that (E9) and (E13) hold (E6) with the 1/2 factor since $\frac{1}{2}\frac{4\pi}{c}\mathbf{m}^* \times \mathbf{J} = -2i\pi k \delta(\mathbf{r})\,\mathbf{m}^* \times \mathbf{p}$.

Therefore, the real and imaginary parts of the force on the Gilbert dipole are respectively:

$$< \boldsymbol{\mathcal{F}}_{mcd} >_i = \frac{1}{2}\mathrm{Re}\{m_j \partial_i B_j^*\} + 2\pi k \delta(\mathbf{r})\mathrm{Im}\{\mathbf{p} \times \mathbf{m}^*\}_i, \tag{E14}$$

and

$$\mathrm{Im}\{\boldsymbol{\mathcal{F}}_{mcd}\}_i = -\frac{1}{2}\mathrm{Im}\{m_j \partial_i B_j^*\} - 2\pi k \delta(\mathbf{r})\mathrm{Re}\{\mathbf{p} \times \mathbf{m}^*\}_i, \quad (i,j = 1,2,3) \tag{E15}$$

At this stage we should remark that for a Gilbert dipole model the Maxwell equation is no longer $\nabla \times \mathbf{E} = ik\mathbf{B}$, but $\nabla \times \mathbf{E} = ik\mathbf{B} - \frac{c}{4\pi}\mathbf{J}_{mc}$, where $\mathbf{J}_{mc} = -i\omega\mathbf{m}\delta(\mathbf{r})$, as seen above. So instead of Eq. (E2), valid for an electric dipole without magnetic charges, the equation for the complex force on the electric dipole if one assumes the existence of magnetic charges is

$$(\boldsymbol{\mathcal{F}})_i = \frac{1}{2}p_j^*\partial_i E_j + 2i\pi k\delta(\mathbf{r})(\mathbf{m}\times\mathbf{p}^*)_i. \tag{E16}$$

Whose real and imaginary parts are

$$< \boldsymbol{\mathcal{F}} >_i = \frac{1}{2}\mathrm{Re}\{p_j\partial_i E_j^*\} - 2\pi k\delta(\mathbf{r})\mathrm{Im}\{\mathbf{p}\times\mathbf{m}^*\}_i. \tag{E17}$$

and

$$\mathrm{Im}\{\boldsymbol{\mathcal{F}}\}_i = -\frac{1}{2}\mathrm{Im}\{p_j\partial_i E_j^*\} - 2\pi k\delta(\mathbf{r})\mathrm{Re}\{\mathbf{p}\times\mathbf{m}^*\}_i, \quad (i,j=1,2,3). \tag{E18}$$

3. ELECTRIC AND MAGNETIC DIPOLE

Therefore, we conclude that if magnetic charges are assumed, the real and imaginary parts of the resulting electric and magnetic dipole forces are:

$$< \boldsymbol{\mathcal{F}} >_i + < \boldsymbol{\mathcal{F}}_{mcd} >_i = < \boldsymbol{F} >_i + < \boldsymbol{\mathcal{F}}_{cld} >_i = \frac{1}{2}\mathrm{Re}\{p_j\partial_i E_j^*\} + \frac{1}{2}\mathrm{Re}\{m_j\partial_i B_j^*\}, \tag{E19}$$

and our proposed expression:

$$\mathrm{Im}\{\boldsymbol{\mathcal{F}}\}_i + \mathrm{Im}\{\boldsymbol{\mathcal{F}}_{mcd}\}_i = -\frac{1}{2}\mathrm{Im}\{p_j\partial_i E_j^*\} - \frac{1}{2}\mathrm{Im}\{m_j\partial_i B_j^*\} - 4\pi k\delta(\mathbf{r})\mathrm{Re}\{\mathbf{p}\times\mathbf{m}^*\}_i. \tag{E20}$$

While in the model in which no magnetic charges are present, we obtain for the resulting reactive force

$$\mathrm{Im}\{\boldsymbol{F}\}_i + \mathrm{Im}\{\boldsymbol{\mathcal{F}}_{cld}\}_i = -\frac{1}{2}\mathrm{Im}\{p_j\partial_i E_j^*\} - \frac{1}{2}\mathrm{Im}\{m_j\partial_i B_j^*\}. \tag{E21}$$

Which in contrast with the time-averaged force which is the same for the *cld* amd *mcd* models, (E21) differs from (E20) by $4\pi k\delta(\mathbf{r})\mathrm{Re}\{\mathbf{p}\times\mathbf{m}^*\}_i$.

This procedure does not yield for the RLF (E19) the interference term [cf. Eq. (D13)]: $-\frac{k^4}{3}\mathrm{Re}\{\mathbf{p}\times\mathbf{m}^*\}$ [4, 16] of the electric and magnetic dipoles. Therefore it is likely that the ILF so obtained, although valid for pure electric or magnetic dipoles, be not valid for magnetoelectric dipolar particles.

From Eq. (A2) we guess for the reactive spin torque:

$$\Xi^{spin} = -\frac{1}{5}\text{Im}[\mathbf{p} \times \mathbf{E}^* + \mathbf{m} \times \mathbf{B}^*] + \frac{k}{c}\int_V d^3r\,(\mathbf{L}_e^O - \mathbf{L}_m^O). \tag{E22}$$

Notice that, again, the diagonal terms of the complex MST do not contribute to the recoil, or scattering, component [4] of Ξ^{spin}.

Or in terms of the above quoted the electric and magnetic angular momenta: $\mathscr{F}_e$ and $\mathscr{F}_m$, we write

$$\Xi^{spin} = -\frac{k}{c}\{\frac{1}{5}(\alpha_e^I \mathscr{F}_e + \alpha_m^I \mathscr{F}_m) - \int_V d^3r\,(\mathbf{L}_e^O - \mathbf{L}_m^O)\}. \tag{E23}$$

Of course the angular orbital momenta, like we saw above for the orbital momenta, store power of the propagating plane wave components of the wavefields.

APPENDIX F: THE REACTIVE FORCE FROM AN INCIDENT PLANE WAVE WITH SPIN ANGULAR MOMENTUM

Figure S1 compares results from a linearly polarized (LP) plane wave with those of circular polarization (CP), both incident on the PS particle of Example 2, and propagating along OZ. As an example, Fig. S1(a) depicts the field intensity in and around the PS particle for CP and LP illumination. Under LP light the field has a butterfly-shape intensity distribution, expected from a dipolar particle, while the intensity excited by CP light is uniform along the azimuthal direction, because of the rotational symmetry of the system. On the other hand, the interaction of particle with plane waves yields a longitudinal component of the field, whose phase remarkably discriminates LP and CP illumination. As shown in Fig. S1(b), the LP light leads to a phase jump at the $x = 0$ plane; while CP illumination yields a phase distribution with a singular point, or vortex, which is known as the result of the spin-to-orbit coupling. However, despite these significant differences in the characteristics of the fields, the ILF produced by the CP illumination is identical to that from LP light [cf. Fig. 2(b)], as illustrated in Fig. S1(c).

APPENDIX G: ON THE CALCULATION OF THE *ROM*

Both $< \boldsymbol{\mathcal{F}} >$ and $\text{Im}\{\boldsymbol{\mathcal{F}}\}$ are calculated from the complex Lorentz integral in V_0, Eq.(6). The induced densities ρ and $\mathbf{J}$ are obtained from the constitutive relations on the total field

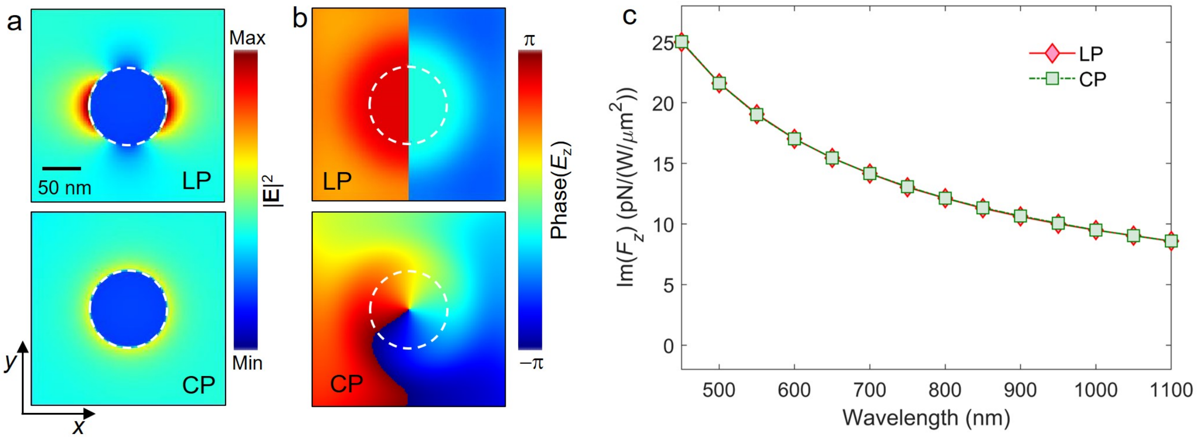

FIG. S 1: Comparison of field distribution and reactive force for the PS particle of Fig.2 when the incident plane wave is linearly polarized (LP), [amplitude $E_0(1,0,0)$, $E_0 = 1$], and circularly polarized (CP) [amplitude $E_0(1,\mathrm{i},0)$, $E_0 = 1/\sqrt{2}$], at the wavelength of 800 nm. (a) Field intensity distribution. (b) Phase profile of the z-component of the electric field in and around the particle after scattering, for LP and CP illumination. Dashed lines outline the particle contour. The sharp vertical line in the upper right figure indicates that for LP illumination, after the light-particle interaction, the resulting electric field z-component has opposite signs on the left and right side of the $x = 0$ plane. (Notice that the space shown in the figures is the XY-plane where the particle center is located). (c) ILF spectrum for LP and CP light.

$\mathbf{E} = \mathbf{E}^{(i)} + \mathbf{E}^{(s)}$, $\mathbf{B} = \mathbf{B}^{(i)} + \mathbf{B}^{(s)}$ via the first and fourth Maxwell equations, respectively.

In our numerical method, the total field is obtained using the commercial software package "FDTD Solutions" (Lumerical, Inc.). The simulation region is $0.22 \times 0.22 \times 0.22$ μm^3, and a uniform mesh with grid size of 5 nm was used. We compute the complex Lorentz force by the expression: $\boldsymbol{\mathcal{F}} = \frac{1}{2}\int_V [\rho^* \mathbf{E}(\mathbf{r}) + \frac{\mathbf{J}^*}{c} \times \mathbf{B}(\mathbf{r})]\, d^3r$, determining ρ and $\mathbf{J}$ via the procedure described in [20]. Then we evaluate the IMST across the surface of a cube that encloses the spherical dipolar particle of volume V_0. Let the volume of this cube be denoted V_q. The volume of the four corners between V_q and V_0 is V_{4c}, its surface being ∂V_{4c}

Obviously since $\rho = 0$ and $\mathbf{J} = 0$ outside V_0, we have from the total field $\mathbf{E} = \mathbf{E}^{(i)} + \mathbf{E}^{(s)}$, $\mathbf{B} = \mathbf{B}^{(i)} + \mathbf{B}^{(s)}$ the following:

$$0 = \mathrm{Im}\{\boldsymbol{\mathcal{F}}\}_{4c} = \int_{\partial V_{4c}} d^2r\, \mathrm{Im}\{T_{ij}\} n_j + i\omega \int_{V_{4c}} d^3r\, [\mathbf{P}_m^O - \mathbf{P}_e^O]_i\,, \tag{G1}$$

I.e.

$$\int_{\partial V_{4c}} d^2r\, \mathrm{Im}\{T_{ij}\} n_j = -i\omega \int_{V_{4c}} d^3r\, [\mathbf{P}_m^O - \mathbf{P}_e^O]_i\,. \tag{G2}$$

But

$$\int_{\partial V_{4c}} d^2r\, \mathrm{Im}\{T_{ij}\} n_j = \int_{\partial V_q - \partial V_0} d^2r\, T_{ij} n_j\,. \tag{G3}$$

Then from (G2):

$$i\omega \int_{V_q} d^3r\, [\mathbf{P}_m^O - \mathbf{P}_e^O]_i = i\omega \int_{V_0} d^3r\, [\mathbf{P}_m^O - \mathbf{P}_e^O]_i - \int_{\partial V_{4c}} d^2r\, \mathrm{Im}\{T_{ij}\} n_j\,. \tag{G4}$$

And from (G3) and (G4) one derives:

$$i\omega \int_{V_q} d^3r\, [\mathbf{P}_m^O - \mathbf{P}_e^O]_i = i\omega \int_{V_0} d^3r\, [\mathbf{P}_m^O - \mathbf{P}_e^O]_i + \int_{\partial V_0 - \partial V_q} d^2r\, \mathrm{Im}\{T_{ij}\} n_j\,. \tag{G5}$$

Which taking into account (57) for $V = V_q$, leads to

$$i\omega \int_{V_q} d^3r\, [\mathbf{P}_m^O - \mathbf{P}_e^O]_i = \mathrm{Im}\{\boldsymbol{\mathcal{F}}_i\} - \int_{\partial V_q} d^2r\, \mathrm{Im}\{T_{ij}\} n_j\,. \tag{G6}$$

Equation (G6) is the procedure we use to calculate the ROM integrated in the volume V_q from the two terms of its right side previously computed as described above.

---

[1] J.D. Jackson, *Classical Electrodynamics*, 2nd edn. J. Wiley (New York, 1975).

[2] M. Born and E. Wolf, *Principles of Optics*, Cambridge University Press, Cambridge (1995).

[3] L. Mandel and E.Wolf, *Optical Coherence and Quantum Optics*, Cambridge University Press, Cambridge, 1995).

[4] M. Nieto-Vesperinas, J. J. Saenz, R. Gomez-Medina and L. Chantada, Optical forces on small magnetodielectric particles, Opt. Express **18**, 11428 (2010).

[5] M. Nieto-Vesperinas, R. Gomez-Medina and J. J. Saenz, Angle-suppressed scattering and optical forces on submicrometer dielectric particles, J. Opt. Soc. Am. A **28**, 54 (2011).

[6] K. Y. Bliokh, A. Y. Bekshaev and F. Nori, Dual electromagnetism: helicity, spin, momentum and angular momentum, New J. Phys. **15**, 033026 (2013).

[7] T. H. Boyer, The force on a magnetic dipole, Am. J. Phys. **56**, 688 (1988).

[8] P. A. M. Dirac, Quantised singularities in the electromagnetic field, Proc. R. Soc. Lond. A **133**, 60 (1931); P. A. M. Dirac, The theory of magnetic poles, Phys. Rev. **74**, 817 (1948).

[9] N. Cabibbo and E. Ferrari, Quantum electrodynamics with Dirac monopoles, Nuovo Cimento **23** 1147 (1962).

[10] J. Schwinger, Magnetic charge and quantum field theory, Phys. Rev. **144**, 1087 (1966).

[11] R. A. Brandt, F. Neri and D. Zwanziger, Lorentz invariance from classical particle paths in quantum field theory of electric and magnetic charge, Phys. Rev. D **19**, 1153 (1979).

[12] K. Li, W-.J. Chen and C. Naon, Classical Electromagnetic Field Theory in the Presence of Magnetic Sources, Chin. Phys. Lett. **20**, 321 (2003).

[13] L. D. Landau and E.M. Lifshitz, The Classical Theory of Fields (Pergamon Press, N.Y. 1994).

[14] M. Nieto-Vesperinas, *Scattering and Diffraction in Physical Optics*, 2nd ed. (World Scientific, Singapore, 2006).

[15] P. C. Chaumet and M. Nieto-Vesperinas, Time-averaged total force on a dipolar sphere in an electromagnetic field, Opt. Lett. **25**, 1065 (2000).

[16] P. C. Chaumet and A. Rahmani, Electromagnetic force and torque on magnetic and negative index scatterers. Opt Express **17** 2224 (2009).

[17] L. Vaidman, Torque and force on a magnetic dipole, Am. J. Phys. **56** 978 (1990).

[18] K. T. McDonald, Forces on Magnetic Dipoles (2018). https://www.physics.princeton.edu/ mcdonald/examples/neutron.pdf.

[19] W. Shockley and R. P. James, “Try Simplest Cases” Discovery of “Hidden Momentum” Forces on “Magnetic Currents”, Phys. Rev. Lett. **18**, 876 (1967).

[20] Ansys/Lumerical, Inc., https://support.lumerical.com/hc/en-us/articles/360042214594-Methodology-for-optical-force-calculations.
